# Rice apoplastic CBM1-interacting protein counters blast pathogen invasion by binding conserved carbohydrate binding module 1 motif of fungal proteins

**DOI:** 10.1371/journal.ppat.1010792

**Published:** 2022-09-29

**Authors:** Takumi Takeda, Machiko Takahashi, Motoki Shimizu, Yu Sugihara, Tetsuro Yamashita, Hiromasa Saitoh, Koki Fujisaki, Kazuya Ishikawa, Hiroe Utsushi, Eiko Kanzaki, Yuichi Sakamoto, Akira Abe, Ryohei Terauchi

**Affiliations:** 1 Iwate Biotechnology Research Center, Kitakami, Iwate, Japan; 2 Laboratory of Crop Evolution, Graduate School of Agriculture, Kyoto University, Mozume, Muko, Kyoto, Japan; 3 Faculty of Agriculture, Iwate University, Iwate, Japan; 4 Department of Molecular Microbiology, Tokyo University of Agriculture, Setagaya-ku, Tokyo, Japan; Nanjing Agricultural University, CHINA

## Abstract

When infecting plants, fungal pathogens secrete cell wall-degrading enzymes (CWDEs) that break down cellulose and hemicellulose, the primary components of plant cell walls. Some fungal CWDEs contain a unique domain, named the carbohydrate binding module (CBM), that facilitates their access to polysaccharides. However, little is known about how plants counteract pathogen degradation of their cell walls. Here, we show that the rice cysteine-rich repeat secretion protein OsRMC binds to and inhibits xylanase MoCel10A of the blast fungus pathogen *Magnaporthe oryzae*, interfering with its access to the rice cell wall and degradation of rice xylan. We found binding of OsRMC to various CBM1-containing enzymes, suggesting that it has a general role in inhibiting the action of CBM1. OsRMC is localized to the apoplast, and its expression is strongly induced in leaves infected with *M*. *oryzae*. Remarkably, knockdown and overexpression of *OsRMC* reduced and enhanced rice defense against *M*. *oryzae*, respectively, demonstrating that inhibition of CBM1-containing fungal enzymes by OsRMC is crucial for rice defense. We also identified additional CBM-interacting proteins (CBMIPs) from *Arabidopsis thaliana* and *Setaria italica*, indicating that a wide range of plants counteract pathogens through this mechanism.

## Author summary

Plants have evolved various activity-inhibiting proteins as a defense against fungal cell wall-degrading enzymes (CWDEs), but how plants counteract the function of fungal enzymes containing carbohydrate binding modules (CBMs) remains unknown. Here, we demonstrate that OsRMC, a member of the cysteine-rich repeat secretion protein family, interacts with fungal CBM1. OsRMC binding to CBM1 of a blast fungal xylanase blocks access to cellulose, resulting in the inhibition of xylanase enzymatic activity. Our study provides significant insights into plant countermeasures against CWDEs in the apoplastic space during plant-fungal pathogen interactions. It also reveals a molecular function of the DUF26 domain widely distributed in plant proteins.

## Introduction

Plant pathogens have developed various strategies to infect and exploit host plants. In response, plants have evolved a complex and multi-layered immune system to overcome invading pathogens [1,2]. Many plant pathogens invade via the apoplast, or extracellular space, of host plants. To this end, fungal pathogens secrete various effector molecules into the host apoplastic space, including hydrolytic enzymes that degrade plant cell wall polysaccharides [3–5]. Plants counter the pathogens by secreting proteases, hydrolytic enzymes, and activity-inhibiting proteins, and also by inducing immune responses after recognition of pathogen-derived molecules [6–9]. Therefore, study of apoplastic molecules that play key roles in plant pathogen invasion and host immunity is essential for understanding host-pathogen interactions.

The plant apoplastic space is filled with the primary cell wall, mainly composed of the polysaccharides cellulose, hemicellulose, and pectin. Hemicellulosic polysaccharides play an important role in controlling the physical properties of the cell wall. Xyloglucan in dicotyledonous and xylan in monocotyledonous plants are the major hemicellulosic polysaccharides by quantity and strengthen the cell wall by forming cross-bridges between cellulose microfibrils [10,11]. A cell wall composed of heteropolysaccharides also provides a physical barrier against plant pathogen invasion [12].

Plant pathogenic fungi secrete a battery of cell wall-degrading enzymes (CWDEs) that catalyze hydrolytic and oxidative degradation of plant cell wall polysaccharides, assisting fungal penetration and colonization. Sugars released from degraded cell walls serve as a carbon source for the pathogens. Notably, some CWDEs possess a carbohydrate binding module (CBM) connected to the catalytic core domain by a linker peptide. CBMs are classified into 88 subgroups (CAZy, http://www.cazy.org), of which CBM family 1 (CBM1) specifically binds to cellulose and is found only in fungal enzymes [13–15]. Critical roles of CBMs in the CWDE-mediated hydrolysis of water-insoluble substrates have been demonstrated. CBM-truncated versions of CWDEs show reduced hydrolytic activities, whereas addition of an extra CBM enhances hydrolytic activities compared with those of wild-type proteins [16–18]. Direct application of a CBM1-containing xylanase (MoCel10A) from *Magnaporthe oryzae* to wheat coleoptile segments reduced cell wall strength to a greater degree than xylanase lacking CBM1 [11]. These observations highlight the role of CBM-containing CWDEs in plant cell wall degradation. Plants counter this enzymatic hydrolysis by pathogens by producing proteins that inhibit enzyme activities of xyloglucan-specific endoglucanase, xylanase, and polygalacturonase [1,19–22]. However, plant proteins that inhibit CBM function have not yet been reported.

Cysteine-rich repeat secretion proteins (CRRSPs) are widespread in land plants and consist of a secretion signal sequence and one or more repeats of the domain of unknown function 26 (DUF26, PF01657) containing a cysteine-rich repeat motif (CRR motif, C-X8-C-X2-C). Ginkbilobin-2 (Gnk2), found in the endosperm of *Ginkgo biloba* seed, is an extracellular CRRSP containing a single DUF26 domain with antifungal activity [23]. Biochemical and structural analyses of Gnk2 revealed a mannose-binding protein composed of two α-helices and a five-stranded β-sheet [24,25], while mutant studies showed a correlation between Gnk2 mannose-binding and antifungal activity [25]. The jasmonic acid (JA)-induced rice protein OsRMC with two DUF26 domains is reported to be highly expressed in root and leaves, and involved in root meander curling and salt stress responses; however, its biochemical function has not been determined [26,27]. Ma et al. [28] reported that the apoplastic effector Rsp3 of the smut fungus *Ustilago maydis* binds maize (*Zea mays*) extracellular DUF26-containing proteins AFP1 and AFP2. AFP1 binds mannose and exhibits antifungal activity against *U*. *maydis*, which is blocked by the Rsp3 effector. Virus-mediated gene silencing of *AFP1* and *AFP2* in maize enhances the virulence of *U*. *maydis*, suggesting that AFP1 and AFP2 may function in host defense. However, the molecular mechanism of AFP1 and AFP2 antifungal activity has not been elucidated. Cysteine-rich repeat kinases (CRKs), members of the receptor-like kinase (RLK) family, are composed of an extracellular domain with two DUF26 repeats, a transmembrane domain, and a C-terminal cytoplasmic Ser/Thr kinase domain. Forty-four CRK genes are known in the *Arabidopsis thaliana* genome, representing one of the largest groups of RLKs [29]. CRKs are induced by reactive oxygen species (ROS) [30] and salicylic acid [30,31] and are involved in defense responses against pathogens [32–37]. CRKs are hypothesized to be involved in ROS/redox signaling and sensing, mediated by the conserved cysteine residues in the DUF26 domains [36,38]. Plasmodesmata-localized proteins (PDLPs), composed of two DUF26 domains in their extracellular region and a transmembrane domain, are involved in cytoplasmic signaling [39,40], pathogen response, and control of callose deposition [41,42]. Despite an increasing number of studies of CRKs and PDLPs, the molecular function of their DUF26 domains remains elusive. A recent amino acid sequence comparison of DUF26-containing proteins revealed the possible evolutionary history of this protein family [43]. CRKs consisting of an extracellular DUF26 domain fused with a transmembrane domain and cytosolic kinase domain might have evolved from an ancestral protein with a single DUF26 domain. After the emergence of the ancestral CRK, the DUF26 domain duplicated, resulting in CRKs with two DUF26 domains. CRRSPs and PDLPs most likely originated from CRKs by deletion of both the transmembrane domain and kinase domain (CRRSPs) or just the kinase domain (PDLPs). The first and second DUF26 domains (DUF26-A and DUF26-B) in CRRSPs are phylogenetically distinct [43].

Here, we report OsRMC, a CBM1-interacting protein (CBMIP) of rice (*Oryza sativa*). OsRMC is a member of the CRRSPs containing two DUF26 domains that binds *M*. *oryzae* CBM1-containing CWDEs. OsRMC inhibits cell wall-degrading activity of fungal enzymes and plays an important role in rice defense against *M*. *oryzae* infection. We also show that CBM1-binding CRRSPs are widespread among plant species. Our findings provide insight into the apoplastic molecular interactions between pathogen cell wall-degrading enzymes and plant proteins containing DUF26 domains.

## Results

### Rice OsRMC, a CRRSP, binds CBM1 and inhibits blast pathogen xylanase activity

**Fig 1:**
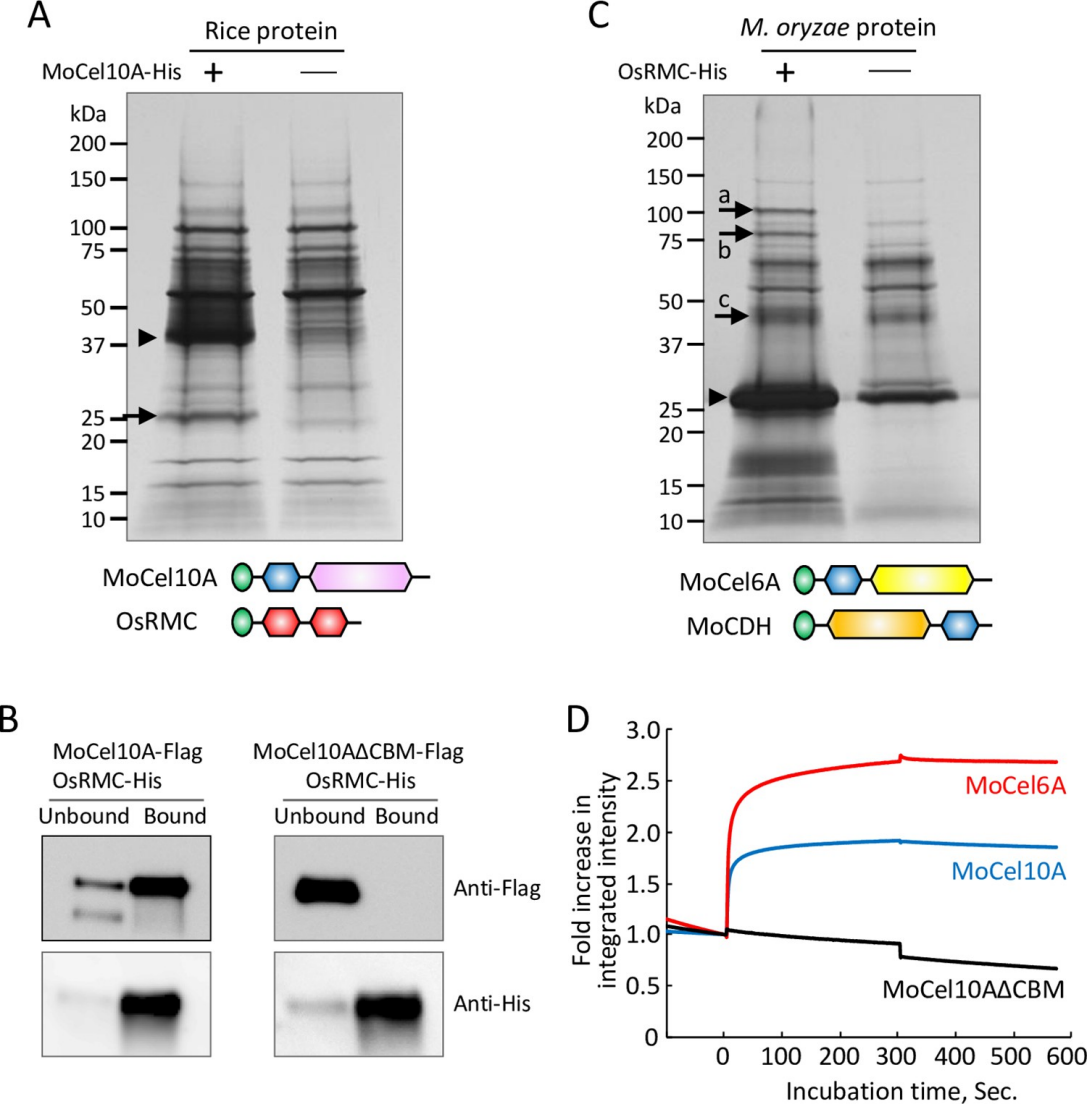
OsRMC binds to CBM1 of fungal enzymes. (A) Protein extract from rice leaves 4 days after inoculation with *M*. *oryzae* (Ken53-33) was incubated with (+) or without (-) MoCel10A-His in the presence of His-resin. Fractions bound to His-resin were subjected to SDS-PAGE followed by silver staining. Arrowhead indicates MoCel10A-His. The protein band indicated by an arrow was identified by LC-MS/MS. Schematic structures of predicted full-length proteins of MoCel10A and OsRMC are shown: green, secretion signal peptide; blue, CBM1 (PF00734); pink, GH10 catalytic core domain (PF00331); red, DUF26 domain (PF01657). (B) OsRMC-His mixed with MoCel10A-Flag or MoCel10AΔCBM-Flag was applied to His-resin. Fractions unbound and bound to His-resin were subjected to SDS-PAGE followed by immunoblot analysis using anti-Flag and anti-His antibodies. (C) Culture filtrate from *M*. *oryzae* hyphae liquid culture was incubated with (+) or without (-) OsRMC-His in the presence of His-resin. Fractions bound to His-resin were subjected to SDS-PAGE followed by silver staining. Arrowhead and arrows indicate OsRMC-His and candidate proteins bound to OsRMC-His, respectively. Schematic structures of MoCel6A and OsCDH are shown: green, secretion signal peptide; blue, CBM1 (PF00734); yellow, GH6 catalytic core domain (PF01341); orange, CDH-cyt domain (PF16010). (D) Integrated intensity representing the binding kinetics of OsRMC to MoCel10A, MoCel6A, and MoCel10AΔCBM. Association of MoCel10A, MoCel6A, and MoCel10AΔCBM (10 μL, 2.0 μM) with OsRMC was measured from time 0 to 300 s. Binding buffer was used to measure protein dissociation from time 300 to 570 s. Fold increase in integrated intensity was calculated by dividing each trajectory by the value at time zero. Each curve is representative of three assays. Binding kinetic values were determined by calculating means ± SD of three independent determinations.

To identify rice proteins that interact with CBM1, we incubated protein extract prepared from rice leaves 4 days after *M*. *oryzae* inoculation with a His-tagged xylanase protein from *M*. *oryzae* (MoCel10A-His), which contains CBM1 and belongs to glycoside hydrolase family 10 [11]. The fraction bound to His-tag affinity resin (His-resin) was analyzed by SDS-PAGE and protein staining. We detected a 25-kDa protein band from the mixture of rice protein extract and MoCel10A-His but not from the rice protein extract alone (Fig 1A). Liquid chromatograph-tandem mass spectrometry (LC-MS/MS) analysis of the 25-kDa band revealed that the protein was a product of *OsRMC* [26,27], encoding a protein with an N-terminal secretion signal peptide and a pair of DUF26 domains (Figs 1A and S1). We generated rice suspension-cultured cells expressing the full-length OsRMC protein fused with a His-tag at its C-terminus driven by the maize ubiquitin promoter. Secreted OsRMC-His protein was mixed with Flag-tagged MoCel10A (MoCel10A-Flag) or CBM1-truncated MoCel10A (MoCel10AΔCBM-Flag), and the protein mixtures were separated into bound and unbound fractions using His-resin. We detected MoCel10A-Flag in the bound fraction and MoCel10AΔCBM-Flag in the unbound fraction (Fig 1B). From this result, we concluded that OsRMC binds to the CBM1 domain of MoCel10A. We also tried to produce MoCel10A in *N*. *benthamiana* in order to do co-immunoprecipitation with OsRMC. However, MoCel10A was not recovered as a soluble protein preparation in this expression system (S2 Fig). Gel-permeation chromatography of the mixture of OsRMC-His and MoCel10A-His confirmed that these proteins form a complex, in which the two proteins were detected at higher molecular weight positions compared with the individual molecules (S3 Fig).

To isolate additional secreted proteins of *M*. *oryzae* that interact with OsRMC, we performed a pull-down experiment by incubating OsRMC-His with *M*. *oryzae* culture filtrate followed by recovery of interacting proteins using His-resin (Fig 1C). Protein bands detected from the mixture of OsRMC-His and *M*. *oryzae* culture filtrate, but not from the *M*. *oryzae* culture filtrate alone, were identified as (a) cellobiose dehydrogenase (MoCDH), (b) cellobiohydrolase (MoCel6A), and (c) xylanase (MoCel10A). In all cases, the predicted full-length proteins consisted of a secretion signal peptide, CBM1, and a catalytic core domain. We confirmed the interaction of OsRMC-His with Flag-tagged MoCDH and MoCel6A by pull-down assay (S4 Fig). Furthermore, when we performed pull-down assays using OsRMC-His and the culture filtrate of *Trichoderma reesei*, an ascomycete fungus, we identified six proteins possessing CBM1 in the OsRMC-binding fraction (S5 Fig). These results indicate that OsRMC binds CBM1 of various fungal enzymes.

We then measured the binding kinetics of OsRMC to MoCel10A and MoCel6A, both harboring an N-terminal CBM1 (Fig 1D). Binding of OsRMC to MoCel10A and MoCel6A occurred rapidly, and the resulting complexes were stable. Binding kinetics values of OsRMC with MoCel10A and MoCel6A were as follows: *K*_a_, 2.98 ± 0.87 and 3.89 ± 0.51 (10^4^ M^-1^ s^-1^); *K*_d_, 1.36 ± 0.42 and 8.45 ± 1.39 (10^−4^ s^-1^); *K*_D_, 4.60 ± 0.90 and 2.19 ± 0.31 (10^−8^ M). Binding of OsRMC to MoCel10AΔCBM was not detected. These results suggest that OsRMC binds CBM1 quickly and the resulting complexes are stable.

**Fig 2:**
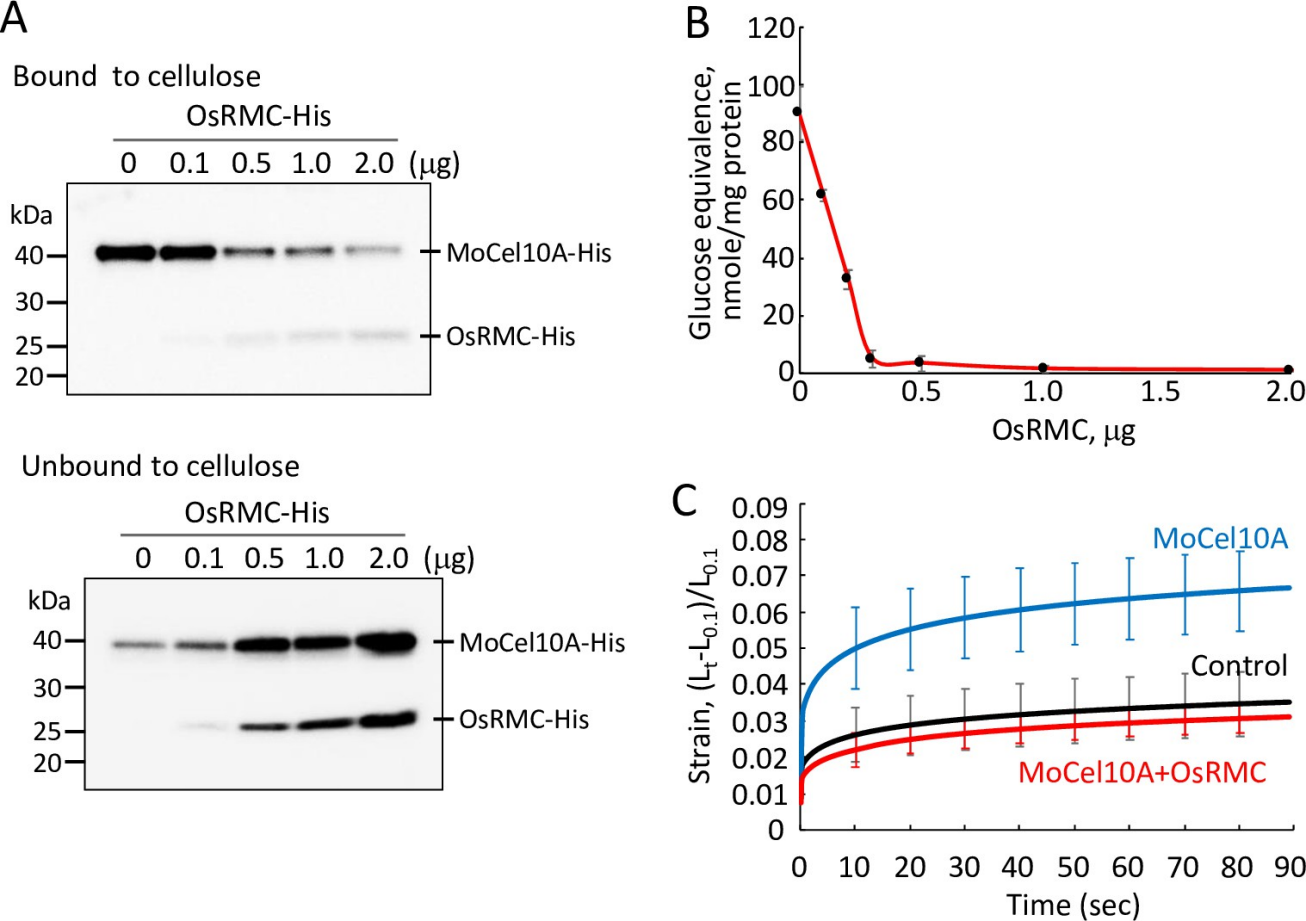
Binding of OsRMC to CBM1 causes to inhibit catalytic activity. (A) MoCel10A-His was incubated with cellulose in the presence of a protein mixture (total 2.0 μg) of OsRMC-His (0–2.0 μg) and BSA (2.0–0 μg) for 1 h at 4°C, and proteins bound and unbound to cellulose were detected by immunoblot analysis using anti-His antibody. (B) MoCel10A-His (2.0 μg) was preincubated with a protein mixture (total 2.0 μg) of OsRMC-His (0–2.0 μg) and BSA (2.0–0 μg) for 30 min 4°C, and the hydrolytic activity towards a wheat cell wall preparation was determined. Data are means ± SD of three independent determinations. (C) Heat-inactivated wheat coleoptile segments were treated with sodium phosphate buffer (100 mM, pH 6.0) containing MoCel10A-His (2.0 μg) with BSA (2.0 μg) or a mixture of MoCel10A-His and OsRMC-His (2.0 μg each). Samples treated with buffer were used as a control. Extension measurement was performed by loading a constant 200 mN to approximately 5-mm segments for 3 min in an extensometer. Strain was calculated as (*L*_*t*_*−L*_0.1_)/*L*_0.1_ (*L*_*t*_, length of coleoptiles at each time point; *L*_0.1_, length of segments at time 0.1). Data are means ± SD of five independent determinations.

CBM1 is known to tightly bind cellulose [13–15]. To investigate the effect of OsRMC on binding of CBM1 to cellulose, we mixed MoCel10A-His with variable amounts of OsRMC-His prior to incubation with cellulose and then detected the resulting cellulose-bound and -unbound MoCel10A-His (40 kDa) as well as OsRMC-His (25 kDa) proteins using an anti-His antibody (Fig 2A). The more OsRMC-His added, the less MoCel10A-His bound to cellulose, indicating that OsRMC interfered with MoCel10A binding to cellulose. We also tested the catalytic activity of MoCel10A-His for xylan hydrolysis in the presence of OsRMC-His using a water-insoluble wheat coleoptile cell wall preparation. The hydrolytic activity of MoCel10A decreased with the increase in OsRMC-His content (Fig 2B). However, MoCel10A hydrolytic activity toward water-soluble xylan was not affected by OsRMC (S6 Fig). These results agree with our previous finding that the presence of CBM1 facilitates enzymatic hydrolysis of water-insoluble polysaccharides [11].

Glucurono-arabinoxylan is a major hemicellulosic polysaccharide of primary cell walls in monocotyledonous plants and is thought to provide cell wall rigidity by forming cross-bridges between cellulose microfibrils. We therefore examined the effects of MoCel10A-His and OsRMC-His on the extensibility of heat-inactivated wheat coleoptile segments (Fig 2C). Treatment with MoCel10A-His enhanced the strain on wheat coleoptile segments, implying a reduction in cell wall strength through the action of MoCel10A. However, addition of OsRMC-His completely blocked the effect of MoCel10A-His. This result indicates that binding of OsRMC to CBM1 inhibits xylan hydrolysis, resulting in suppressed extensibility of coleoptile segments.

### Apoplast-localized OsRMC contributes to rice defense against *M*. *oryzae* infection by CBM1 binding

**Fig 3:**
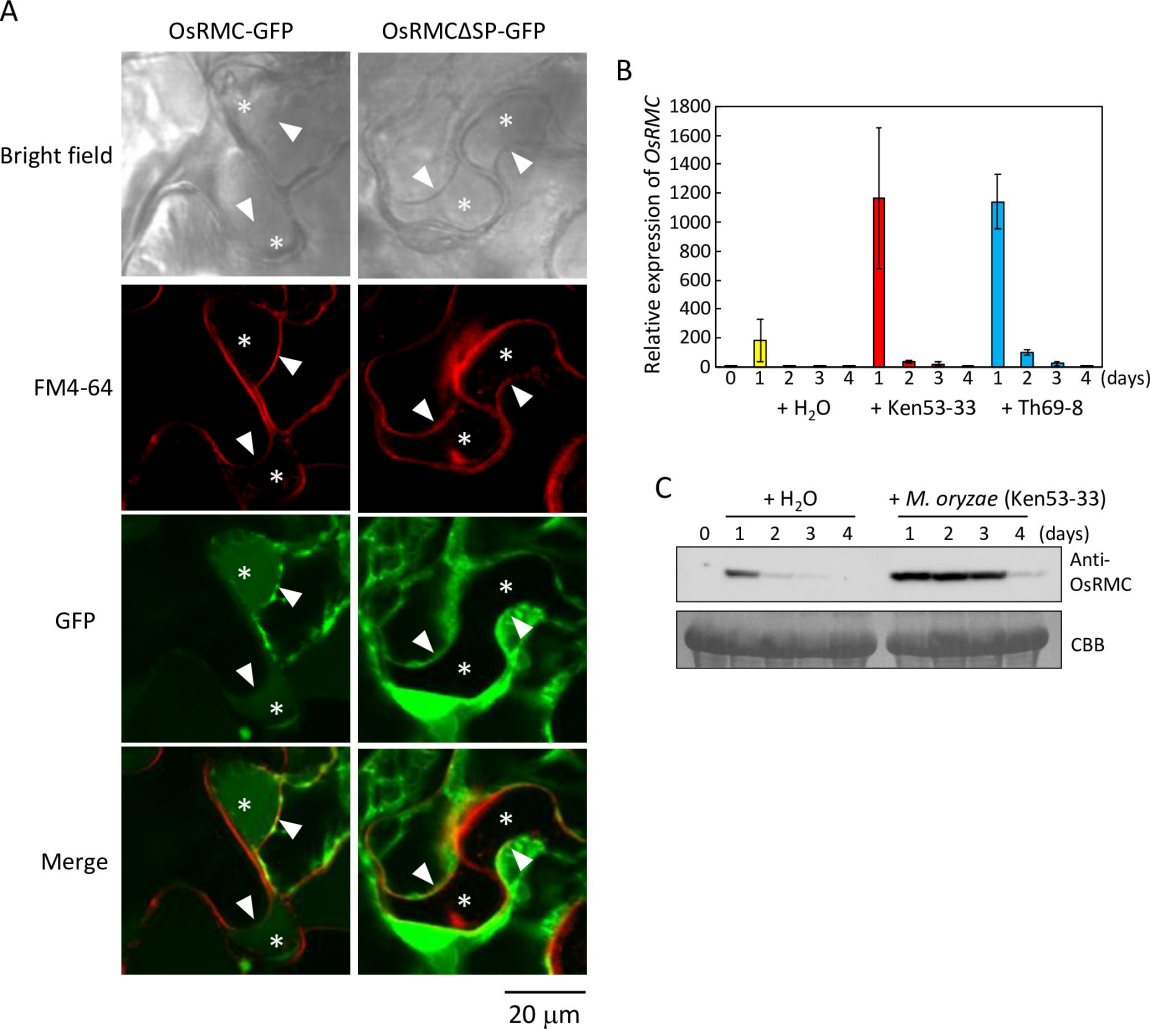
OsRMC is localized to apoplast and the gene expression is induced by *M*. *oryzae* infection. (A) OsRMC is localized to apoplast. Subcellular localization of OsRMC was determined using plasmolyzed leaves of *N*. *benthamiana* overexpressing OsRMC-GFP or OsRMCΔSP-GFP by confocal laser scanning microscopy. The cells were also stained with FM4-64 to label the plasma membrane. Asterisk indicates the generated apoplastic space in the plasmolyzed cells. Arrow head indicates the position of plasma membrane. Bars, 20 μm. (B) *OsRMC* expression induced by *M*. *oryzae* infection in both compatible and incompatible interactions. Expression levels of *OsRMC* transcripts in rice (‘Hitomebore’) leaves after inoculation with *M*. *oryzae* (Ken53-33, compatible strain; TH69-8, incompatible strain, 3.0 X 10^5^ conidia each) were determined by qRT-PCR. *OsRMC* expression level was normalized using that of the rice ubiquitin gene (*LOC_Os03g03920*.*1*). Data are means ± SD of three independent determinations. (C) OsRMC protein expression is induced after *M*. *oryzae* infection. Protein extracts (20 mg) from rice leaves 0–4 days after *M*. *oryzae* (Ken53-33) inoculation were subjected to immunoblot analysis using an anti-OsRMC antibody. Equal protein loading on SDS-PAGE was verified by Coomassie Brilliant Blue (CBB) staining.

To observe the function of OsRMC *in planta*, we transiently expressed GFP-fused OsRMC (OsRMC-GFP) and OsRMC with deletion of the secretion signal peptide sequence (OsRMCΔSP-GFP) in *Nicotiana benthamiana* leaves by agroinfiltration, and observed its subcellular localization in the plasmolyzed cells. Position of cell wall and plasma membrane in the plasmolyzed cells was determined by light field observation and staining with FM4-64. We detected OsRMC-GFP signal in the generated apoplastic space in the plasmolyzed cells, whereas we observed OsRMCΔSP-GFP signal in the cells but not in the apoplastic space (Fig 3A). Protein fractionation revealed OsRMC-GFP in the buffer-soluble fraction but not the membrane fraction of *N*. *benthamiana* leaves (S7 Fig). In addition, OsRMC with C-terminal hemagglutinin epitope tag (HA-tag) overexpressed in rice suspension cells was secreted into the culture medium (S7 Fig). These results indicate that OsRMC is present in the apoplastic space and is not anchored on cellular membranes.

We investigated the expression of *OsRMC* in rice leaves inoculated with *M*. *oryzae* using quantitative reverse-transcription PCR (qRT-PCR) (Fig 3B). The basal level of *OsRMC* expression (at time zero) in leaves was very low. Gene expression was slightly induced 1 day after water treatment as control; by contrast, expression levels were more than fivefold higher than in the control 1 day after inoculation with *M*. *oryzae* isolates Ken53-33 (compatible) and Th69-8 (incompatible). Expression was then rapidly downregulated 2 days after *M*. *oryzae* inoculation. We also evaluated OsRMC protein accumulation in rice leaves by immunoblot analysis using anti-OsRMC antibody (Fig 3C). OsRMC protein was undetectable at time zero and slightly induced at 1 day after water treatment. By contrast, OsRMC accumulated highly at 1–3 days after inoculation with compatible *M*. *oryzae* isolate Ken53-33 and then decreased at 4 days after inoculation. These results suggest that gene expression of *OsRMC* is markedly induced by *M*. *oryzae* inoculation and a high level of OsRMC protein accumulation is maintained for several days.

**Fig 4:**
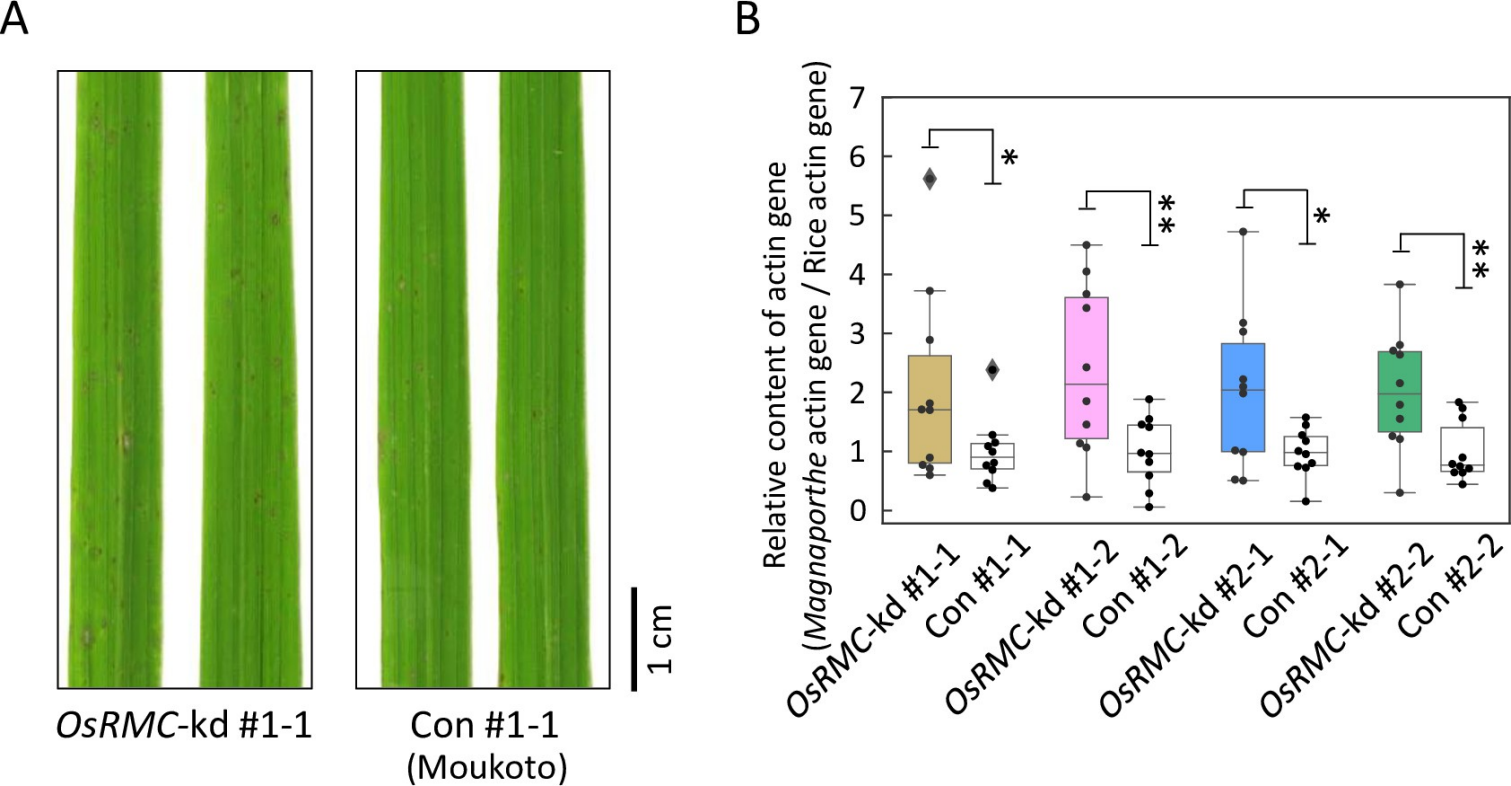
*OsRMC*-knockdown rice plants show reduced resistance to *M*. *oryzae*. (A) Rice leaves of *OsRMC*-knockdown (*OsRMC-*kd) and wild-type (Moukoto) control (Con) lines 4 days after inoculation of *M*. *oryzae* (Ken53-33, 3.0 X 10^5^ conidia). The *M*. *oryzae* infection test was conducted using T_1_ *OsRMC-*kd and wild-type control progenies (*n* = 10) derived from selfing of T_0_ heterozygous *OsRMC*-kd lines. (B) *M*. *oryzae* infection is enhanced in *OsRMC-*kd rice. The amount of *M*. *oryzae* fungal mass in rice leaf was monitored by quantifying the ratio of *M*. *oryzae* genomic DNA to rice genomic DNA, determined by qPCR of the respective *actin* genes. The average ΔΔCt value in wild-type control lines was defined as the unit for the ratio. Data are means ± SD of 10 independent determinations. Single and double asterisks indicate a significant difference at *P* < 0.05 and *P* < 0.01, respectively, according to Student’s *t*-test.

**Fig 5:**
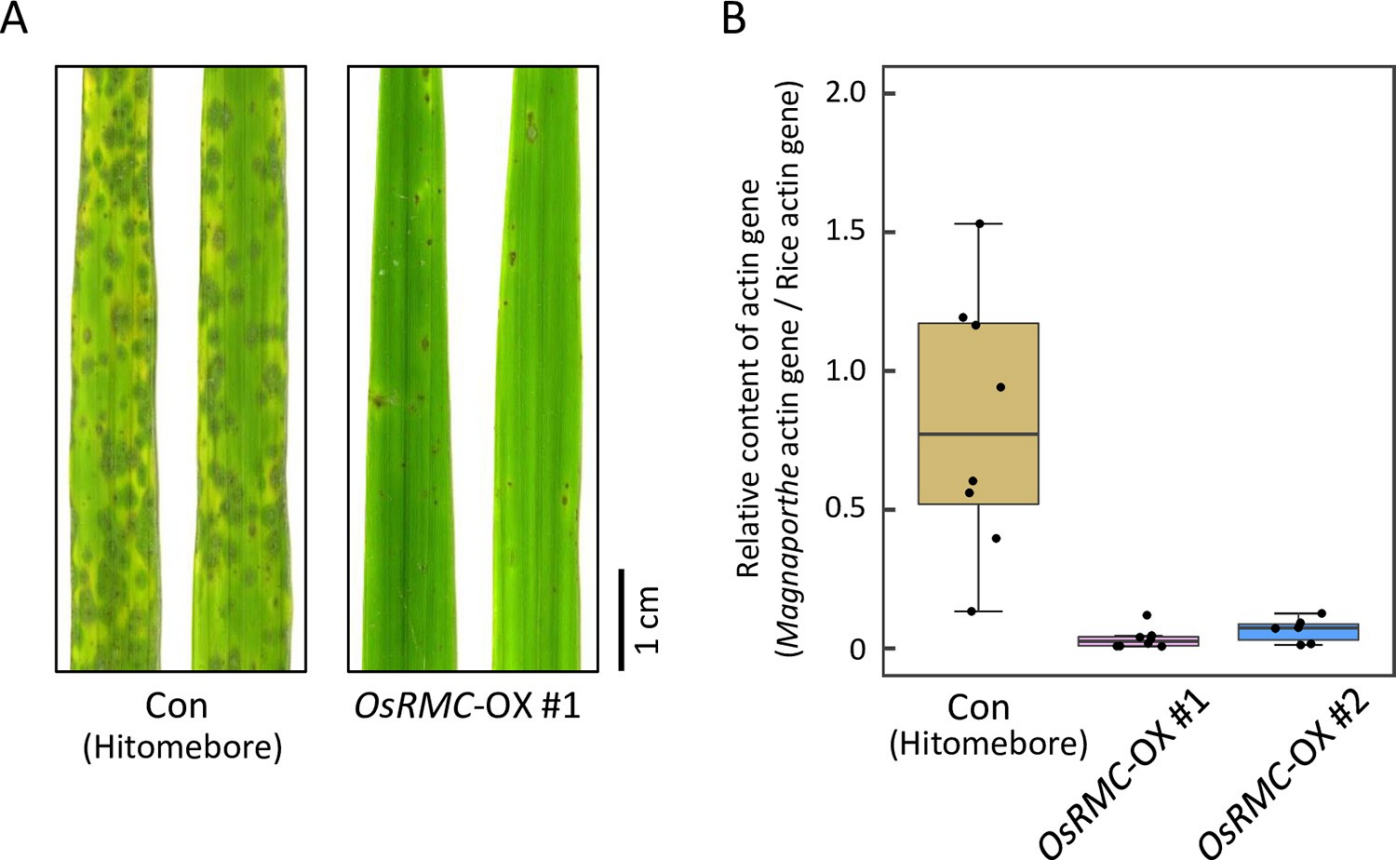
*OsRMC*-overexpressing rice plants show enhanced resistance against *M*. *oryzae*. (A) Rice leaves of wild-type (Hitomebore) control (Con) and *OsRMC*-overexpressing (*OsRMC-*OX) lines 4 days after inoculation of *M*. *oryzae* (Ken53-33, 1.5 X 10^6^ conidia) inoculation. The *M*. *oryzae* infection test was conducted using wild-type control (*n* = 10) and *OsRMC*-OX (#1 [n = 6] and #2 [n = 6]) lines. (B) *M*. *oryzae* infection is suppressed in *OsRMC-*OX lines. The amount of *M*. *oryzae* fungal mass in rice leaf was monitored by quantifying the ratio of *M*. *oryzae* genomic DNA to rice genomic DNA, determined by qPCR of the respective *actin* genes. The average ΔΔCt value in wild-type control lines was defined as the unit for the ratio. Data are means ± SD of independent determinations. Double asterisks indicate a significant difference at *P* < 0.01, respectively, according to Student’s *t*-test.

To investigate the role of OsRMC in rice immunity to *M*. *oryzae*, we performed knockdown of the *OsRMC* gene in rice cultivar ‘Moukoto’ by RNA interference (RNAi)-mediated gene silencing. We generated transgenic lines (#1 and #2) by introducing a gene-silencing vector containing DNA fragments from open reading frame (311 bp) and 3′-untranslated (433 bp) regions of the *OsRMC* transcript, respectively (S8 Fig). Self-propagation of transgenic rice plants of the T_0_ generation produced the T_1_ generation, which segregated for progeny with and without the RNAi construct. We distinguished *OsRMC*-knockdown and *OsRMC*-expressing plants by PCR of the hygromycin gene in the RNAi construct and by immunoblotting of OsRMC (S9 Fig). OsRMC protein accumulation was almost undetectable in *OsRMC*-knockdown lines, but was clearly detected in control lines. These T_1_ progeny were spray-inoculated with *M*. *oryzae* (Ken53-33), which is compatible with the rice cultivar ‘Moukoto’. Four days after *M*. *oryzae* inoculation, disease lesions caused by *M*. *oryzae* infection were visible on the inoculated rice leaves (Figs 4A and S9). More severe disease symptoms were observed in *OsRMC*-knockdown lines #1–1, #1–2, #2–1, and #2–2 than in the corresponding controls. We evaluated *M*. *oryzae* fungal mass in rice leaves by measuring the amount of *M*. *oryzae* genomic DNA normalized to the amount of rice genomic DNA by qPCR of the *ACTIN* gene (Figs 4B and S10). The amount of *M*. *oryzae* fungal mass was significantly higher in leaves of *OsRMC*-knockdown lines #1–1, #1–2, #2–1, and #2–2 than in those of the corresponding controls. We also generated transgenic plants of rice cultivar “Hitomebore” overexpressing *OsRMC* gene driven by maize ubiquitin promoter (*OsRMC*-OX) and studied *M*. *oryzae* infection. Four days after *M*. *oryzae* inoculation, disease lesions caused by *M*. *oryzae* infection were visible on the inoculated wild-type rice leaves (Fig 5A). Less disease symptoms were observed in the two lines of *OsRMC*-OX plants. The levels of *M*. *oryzae ACTIN* DNA normalized to rice *ACTIN* DNA in *OsRMC*-OX lines (both #1 and #2) were markedly reduced as compared to that in the wild-type plants (Fig 5B). These results indicate that OsRMC plays a role in rice defense against *M*. *oryzae* infection.

**Fig 6:**
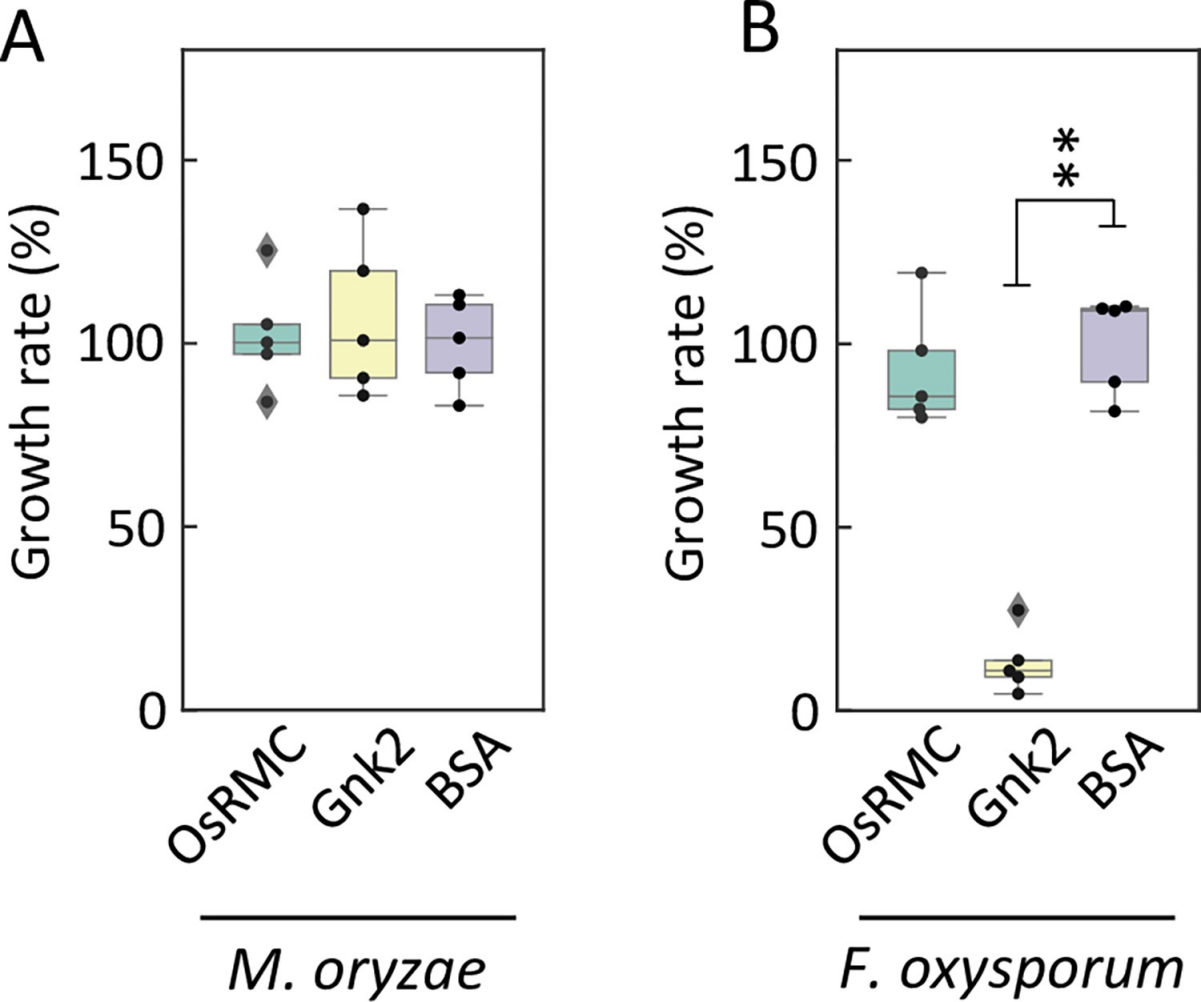
OsRMC does not show antifungal activity against *M*. *oryzae* and *F*. *oxysporum*. (A) Antifungal activity of OsRMC and Gnk2 against *M*. *oryzae* and (B) *F*. *oxysporum*. Cells of *M*. *oryzae* overexpressing luciferase or of *F*. *oxysporum* were cultured in the presence of OsRMC (10 μM, 79 μg/300 μL culture medium) supplemented with 21 μg of BSA, Gnk2 (10 μM, 38 μg/300 μL culture medium) supplemented with 62 μg of BSA, or BSA (100 μg) alone for 36 h at 25°C. Growth of *M*. *oryzae* and *F*. *oxysporum* was evaluated by measuring luciferase activity and optical density at 600 nm, respectively. Data are means ± SD of five independent determinations.

Two CRRSPs, Gnk2 of *G*. *biloba* [23] and AFP1 of maize [28], bind mannose and show antifungal activities. To address whether the rice blast resistance mediated by OsRMC is caused by its antifungal activity, we cultured *M*. *oryzae* and *Fusarium oxysporum* in the presence of OsRMC, Gnk2, or BSA. We observed no difference in the growth of *M*. *oryzae* among the three treatments (Figs 6A and S11). In the case of *F*. *oxysporum*, there was no difference between the OsRMC and BSA treatments at 30 h; however, a significant reduction in growth was observed after the Gnk2 treatment (Fig 6B), as reported previously [25], suggesting that whereas Gnk2 exhibits antifungal activity against *F*. *oxysporum*, OsRMC does not possess antifungal activity against *M*. *oryzae* or *F*. *oxysporum*. This result indicates that the rice defense conferred by *OsRMC* is mediated by OsRMC binding to pathogen CBM1 and inhibition of fungal hydrolases, not by direct antifungal activity of the protein.

### A wide range of plant species have CBMIPs

**Fig 7:**
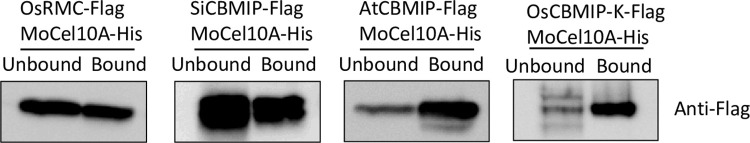
CBM1-binding proteins are widespread in plants. CRRSPs (OsRMC, SiCBMIP, and AtCBMIP) and OsCBMIP-K bind MoCel10A. Flag-tagged CRRSPs and OsCBMIP-K were incubated with MoCel10A-His in the presence of His-resin. Fractions unbound and bound to His-resin were subjected to immunoblot analysis using an anti-Flag antibody.

To investigate whether plant species other than rice have OsRMC-like CBM-binding proteins (CBMIPs), we performed a pull-down assay using MoCel6A-His and protein extract of *Setaria italica* (foxtail millet) leaves inoculated with *M*. *oryzae* (isolate GFSI 1-7-2) (S12 Fig). We detected a protein band in the mixture of *S*. *italica* protein and MoCel6A-His but not in the sample with *S*. *italica* protein alone. This protein was identified as a member of the CRRSPs, named SiCBMIP (XM_004960453.3), which also bound to MoCel10A (Fig 7). We also searched the Arabidopsis gene database for candidate CBMIPs with amino acid sequence similarity to OsRMC and assayed three proteins for their binding to MoCel10A-His (S13 Fig). One of them, encoded by *AT3G22060*.*1*, hereafter named AtCBMIP, showed binding to MoCel10A-His (Figs 7 and S13). These results suggest that a wide range of plant species possess CBM1-binding CRRSPs. CRKs are composed of an extracellular domain with two or more DUF26 domains, a transmembrane domain, and a kinase-like domain. To test whether CRKs bind CBM1, we produced recombinant proteins comprising the extracellular domains from seven rice CRKs in *N*. *benthamiana* leaves and assayed their binding to MoCel10A-His. The extracellular domain (25.1 kDa) and full length (72.0 kDa) encoded by *LOC_Os07g35580*.*1*, hereafter named OsCBMIP-K, bound to MoCel10A (Figs 7 and S14).

**Fig 8:**
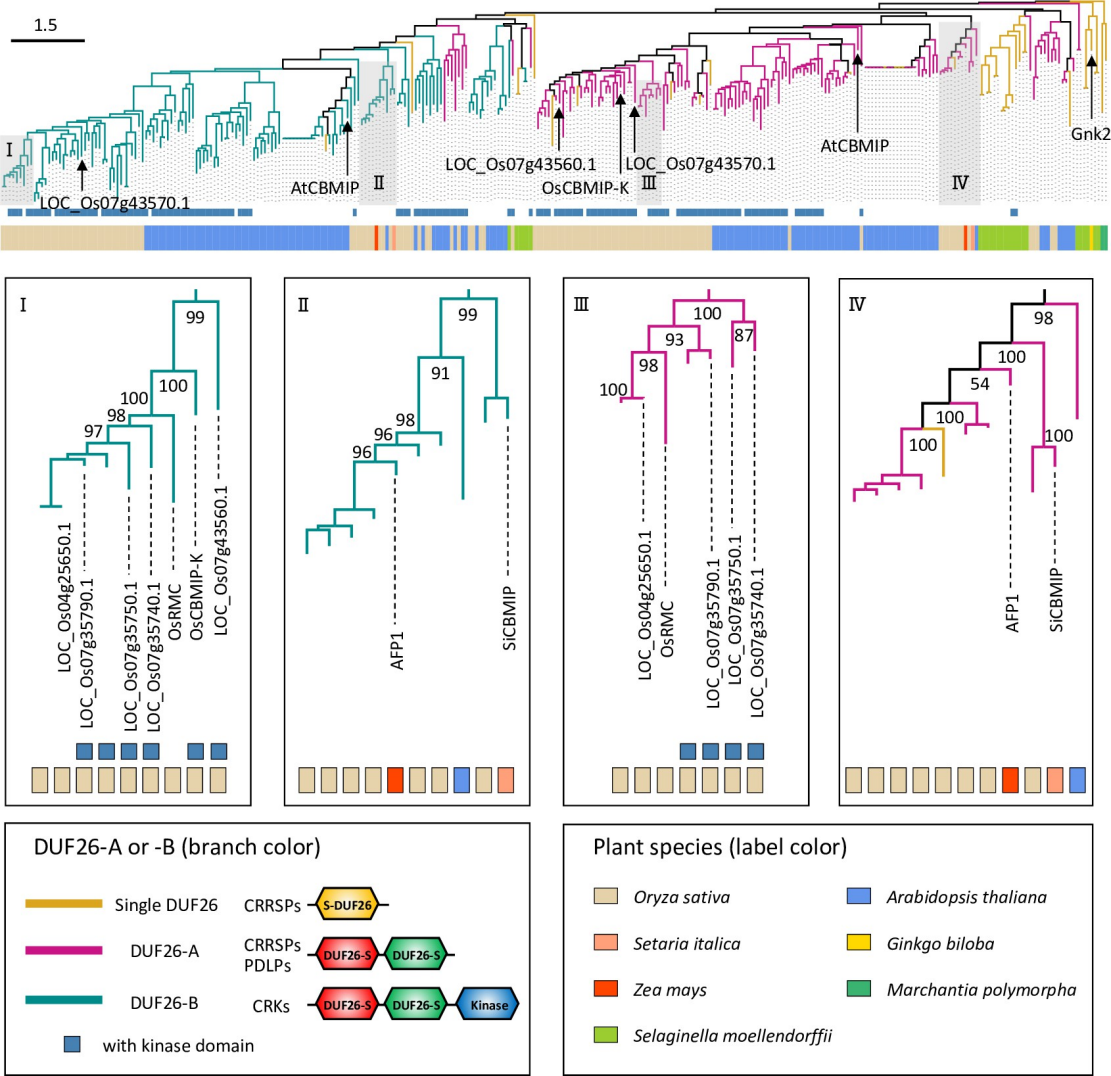
Phylogenetic analysis of CBM1-binding proteins. Phylogenetic tree reconstructed using a single DUF26, DUF26-A and DUF26-B domains of CRRSPs and CRKs from *O*. *sativa*, *A*. *thaliana*, *Selaginella moellendorffii*, and *M*. *polymorpha*, and Gnk2, SiCBMIP, and AFP1. Bar, 1.5 amino acid substitutions per site.

To infer the evolution of CRRSP function, we reconstructed a phylogenetic tree of the DUF26 domain using amino acid sequences of CRRSPs and CRKs from *O*. *sativa* and *A*. *thaliana* together with CRRSPs of *Selaginella moellendorffii* (Lycopodiophyta) and *Marchantia polymorpha* (Bryophyte), Gnk2 of *G*. *biloba*, AFP1 of maize, and SiCBMIP of *S*. *italica* (Fig 8). DUF26 domains in CRRSPs with two such domains were separately treated as DUF26-A (N-terminal) and DUF26-B (C-terminal). In the tree, DUF26-A and DUF26-B form two major clades that diverged from an ancestral singleton DUF26 domain of bryophytes (*M*. *polymorpha*), as suggested by Vaattovaara et al. [43]. Gnk2 of *G*. *biloba*, a mannose-binding single DUF26 domain CRRSP, is located close to the root of the tree. The DUF26-A and DUF26-B domains of maize AFP1, another mannose-binding protein, are positioned close to the roots of their respective clades, which also include the DUF26 domains of SiCBMIP from *S*. *italica*. The DUF26-A and -B domains of each of the three CBM1-binding CRRSPs, OsRMC, AtCBMIP, and SiCBMIP, which share relatively low amino acid identities (27–31%) (S1 Table), are on separate branches in the tree, indicating that CBM1-binding CRRSPs may have evolved independently multiple times in these plant lineages. The DUF26 domains from *O*. *sativa* form large monophyletic groups in the DUF26-A and -B clades, respectively, with CRKs representing the majority of proteins. OsRMC and OsCBMIP-K are phylogenetically close, and both bind CBM1 (Fig 8). These findings indicate that OsRMC most likely originated from OsCBMIP-K by deletion of the transmembrane domain and the kinase domain.

### OsRMC binds mannose as well as CBM1

**Fig 9:**
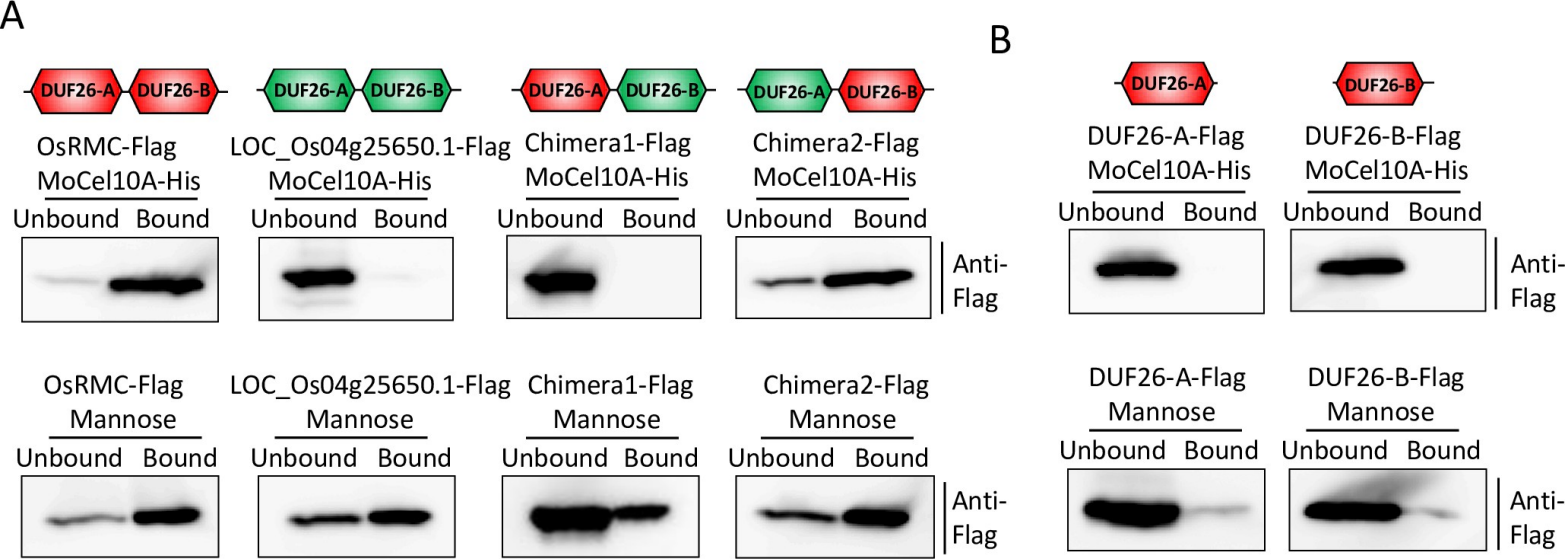
OsRMC DUF26-B determines its binding to CBM1. (A) OsRMC, LOC_Os04g25650.1, and their chimeric proteins were produced in *N*. *benthamiana* and assayed for MoCel10A (CBM1: top) and mannose (bottom) binding. Simplified schemes of OsRMC (235 amino acids; red) and LOC_Os04g25650.1 (251 amino acids; green) as well as two newly generated chimeric proteins, Chimera1 (244 amino acids, comprising 97 aa [24–120] of OsRMC and 147 aa [135–281] of LOC_Os04g25650.1) and Chimera2 (242 amino acids, comprising 104 aa [31–134] of LOC_Os04g25650.1 and 138 aa [121–258] of OsRMC) are shown. Flag-tagged proteins were assayed for binding to MoCel10A-His and to mannose. Unbound and bound fractions were subjected to immunoblot analysis with an anti-Flag antibody. (B) Binding assay of single DUF26 domains (DUF26-A and DUF26-B) of OsRMC to MoCel10A (CBM1: top) and mannose (bottom).

Previous studies showed that the two CRRSPs, Gnk2 of *G*. *biloba* [23,25] and AFP1 of maize [28], bind mannose and exhibit antifungal activities. Therefore, we investigated binding of OsRMC to mannose using a mannose-agarose binding assay (Fig 9A). Incubation of OsRMC with mannose-agarose resulted in recovery of OsRMC in the bound fraction. This result indicates that OsRMC binds mannose as well as CBM1. The DUF26 domains of OsCBMIP-K, the likely progenitor of OsRMC, also bound mannose (S15 Fig). Those of Os07g43560.1, a sister group CRK of OsRMC and OsCBMIP-K, bound mannose but not CBM1, whereas that of Os07g43570.1, distantly related to DUF26 domains in clade I, did not bind mannose or CBM1 (S14 and S15 Figs).

We also tested binding of another rice CRRSP encoded by *LOC_Os04g25650*.*1*, which showed the highest amino acid sequence identity (59%) to OsRMC among rice proteins (S16 Fig). Our phylogenetic analysis indicated that LOC_Os04g25650.1 is likely derived from OsRMC (Fig 8). This protein bound to mannose but not to MoCel10A (Fig 9A).

To infer the roles of the DUF26-A and DUF26-B domains in CBM1 and mannose binding, we generated chimeric proteins by exchanging the DUF26-A and DUF26-B domains of OsRMC and LOC_Os04g25650.1 (Fig 9A) and tested their binding to the two molecules. Both Chimera1 and Chimera2 bound mannose. Chimera2, with DUF26-A from LOC_Os04g25650.1 and DUF26-B from OsRMC, retained CBM1 binding, whereas Chimera1, with DUF26-A from OsRMC and DUF26-B from LOC_Os04g25650.1, lost CBM1 binding, suggesting that the DUF26-B domain determines CBM1-binding capability. A single DUF26 domain of OsRMC was unable to bind mannose or CBM1 (Fig 9B), indicating that both DUF26-A and -B are necessary for binding to these two compounds.

Additionally, we tested binding of other cloned CRRSPs to mannose. AtCBMIP1 and SiCBMIP did not bind mannose (S15 Fig). AFP1 showed binding to mannose at a level lower than that for OsRMC, but did not bind to CBM1 (S17 Fig). Cloned CRRSPs and DUF26 domains of CRKs that did not bind to CBM1 (AT4G20670.1, AT4G23170.1, Os07g35290.1, Os07g35740.1, Os07g35750.1, and Os07g35790.1) did not bind mannose either. These results suggest that the CRRSPs and DUF26 domains of CRKs tested may have other interactors than mannose and CBM1 or that their function may not involve binding to other compounds.

## Discussion

### CBMIP and CBM1: Another front of plant-pathogen protein interactions

**Fig 10:**
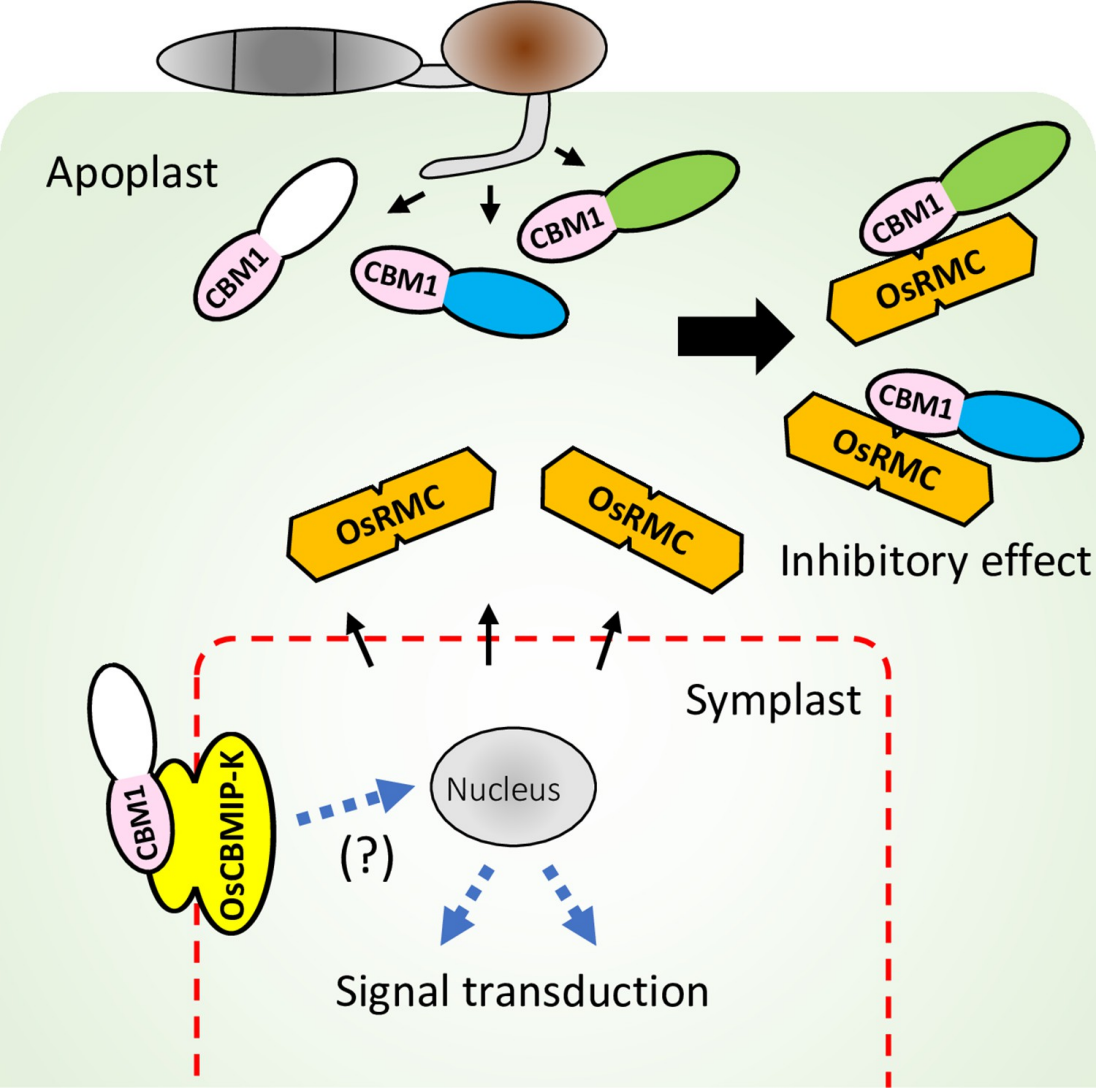
Model of the action of CBM1-binding proteins. Various CBM1-containing CWDEs are secreted from *M*. *oryzae*. OsRMC is secreted to apoplastic space and binds to CBM1 of the enzymes, resulting inhibition of the enzymatic activities. OsCBMIP-K binds to CBM1 of the enzymes and may potentially be involved in signal transduction.

This study identified an apoplastic rice protein with two DUF26 domains that binds CBM1 of blast fungal xylanase MoCel10A. OsRMC binds at least two more blast fungal hydrolases and multiple proteins of *T*. *reesei*, suggesting that CBMIP targets multiple fungal hydrolases. In turn, screening of plant proteins using the CBM1-containing proteins MoCel10 and MoCel6A identified multiple CBMIPs from *S*. *italica* and Arabidopsis. *OsRMC*-knockdown plants displayed reduced resistance against *M*. *oryzae*. *OsRMC*-overexpressing plants showed enhanced resistance. These results indicate that OsRMC plays a key role in rice defense against this pathogen. These results reveal a hitherto undescribed mechanism of plant-pathogen protein interactions mediated by CBMIP and CBM1. We propose that plant apoplastic CBMIP proteins target conserved CBM1 motifs in various hydrolases of fungal pathogens to inhibit the hydrolysis of plant cell wall polysaccharides, resulting in the suppression of pathogen invasion (Fig 10). Roles for DUF26-containing proteins in plant defense have been reported in *G*. *biloba* (Gnk2) and maize (AFP1) [25,28], where the proteins exhibit antifungal activity, presumably mediated by binding to pathogen mannose. OsRMC binds both CBM1 and mannose, but does not show antifungal activity against *M*. *oryzae* or *F*. *oxysporum*, suggesting that its role in defense is related to inhibition of fungal hydrolases mediated by CBM1-binding and is different from the function of Gnk2 and AFP1.

OsRMC was reported to be involved in seed germination and leaf growth under conditions of high NaCl concentration, but its biological function remains unclear [27]. We hypothesize that OsRMC may be a multi-functional protein possibly involved in responses to biotic and abiotic stresses. Future study is needed to elucidate the link between the two processes.

CBMs are widely distributed in fungi, bacteria, and plants (S2 Table). CBM domain 18 (CBM18) is most frequently observed among the *M*. *oryzae* CBM proteins. However, since plants also have CBM18 domains in their own proteins, this domain does not allow plants to discriminate “non-self” from “self.” By contrast, CBM1 is found only in fungal enzymes. We therefore hypothesize that targeting of CBM1 by OsRMC evolved to counteract fungal CWDEs.

### Function of DUF26-containing proteins and the origin of CBMIPs

There are several types of DUF26-containing proteins. The archetypical protein Gnk2 has only a single DUF26 domain, whereas numerous proteins contain two distantly related DUF26 domains, DUF26-A and DUF26-B. CRKs contain an extracellular domain with two DUF26 motifs, a transmembrane domain, and an intracellular kinase domain. PDLPs contain an extracellular domain with two DUF26 motifs and a transmembrane domain. In the majority of cases, binding targets of DUF26 domains are not known. However, Gnk2 and AFP1 are reported to bind mannose. We identified CRRSPs that do not bind either mannose or CBM1 (S13, S14 and S15 Figs, and S3 Table). We hypothesize that the DUF26 domains of the distant progenitor bound an unknown compound, as in the case of Os07g43570.1. After the divergence of clade I DUF26 domains from the rest, some gained the capability to bind mannose, as seen in Os07g43560.1, and subsequently obtained CBM1 binding, as is the case for OsCBMIP-K and OsRMC through amino acid changes in the DUF26-B domain; however, their derivative protein LOC_Os04g25650.1 lost CBM1-binding capability through changes in its DUF26-B domain. Disulfide bridges formed by conserved cysteine residues of the CRR motif of DUF26 are predicted to contribute to structural stabilization [43], and this common fold may have provided CRRSPs with a versatile platform for binding various molecules.

Our phylogenetic analysis indicates that OsRMC is derived from OsCBMIP-K through truncation of the transmembrane domain and kinase domain. Since OsCBMIP-K binds CBM1, there is a possibility of signaling mediated by recognition of CBM1 (Fig 10). Future studies should clarify whether CBM1 recognition by OsCBMIP-K plays a role in defense signaling.

In summary, our study and others suggest that DUF26 is a versatile multi-functional domain deployed by plants to bind various compounds (e.g. mannose, CBM1) and/or sense environmental conditions (e.g. ROS). Accordingly, the genes for DUF26-containing proteins were highly amplified in plant genomes. As shown in this study, a subset of DUF26-domain-containing proteins evolved as apoplastic proteins to counteract pathogens by binding and inactivating CBM1-containing proteins. Future structure analysis would allow engineering of CBMIP for a higher CBM1-binding capability to generate pathogen resistant crops.

## Materials and methods

### Materials

Water-insoluble wheat cell wall material was prepared from wheat coleoptiles by treating sequentially with methanol and amylase.

### Growth conditions of microorganisms and plants

*M*. *oryzae* was grown on oatmeal plates or YG (0.5% yeast extract, 2% glucose, w/v) medium at 25°C. Conidium formation of *M*. *oryzae* was accomplished at 28°C under dark-blue light for 4 days. *Agrobacterium tumefaciens* (GV3101) carrying plasmid vector was grown in YEB (0.5% yeast extract, 1% peptone, 0.5% beef extract, 0.5% sucrose, w/v) medium supplemented with rifampicin and kanamycin. Rice and *N*. *benthamiana* were grown in soil at 30 and 25°C, respectively. Rice suspension cells were cultured at 25°C with rotation at 140 rpm and transferred to fresh medium every 10 days as described previously [44].

### DNA sequencing

DNA was amplified from cDNA by PCR using PrimeStar GXL DNA polymerase (Takara Bio) and verified by DNA sequencing using a 3130 Genetic Analyzer (Applied Biosystems). DNA primers used in this study are shown in S4 Table.

### Recombinant protein preparation

His-tagged or Flag-tagged *M*. *oryzae* proteins—xylanase (MoCel10A), CBM1-truncated xylanase (MoCel10AΔCBM), cellobiohydrolase (MoCel6A), and cellobiose dehydrogenase (MoCDH) were overexpressed in *M*. *oryzae* as described previously [45]. His-tagged and HA-tagged OsRMCs were overexpressed in rice suspension cells as described previously [44]. Flag-tagged CRRSPs and CRKs were expressed in *N*. *benthamiana* leaves as described previously [46]. His-tagged Gnk2 was expressed in *Escherichia coli* (*Origami*) as described previously [25]. His-tagged proteins were purified using His-tag affinity resin (His-resin) (Clontech).

### Pull-down assay with His-tagged proteins

His-tagged MoCel10A and OsRMC were expressed in *M*. *oryzae* and rice suspension cells, respectively. Rice crude protein preparation was extracted from rice leaves 4 days after *M*. *oryzae* inoculation. *M*. *oryzae* and *T*. *reesei* were grown in induction medium (0.5% yeast extract, 0.2% glucose, w/v) containing 0.5% rice leaves and 0.5% cellobiose (w/v), respectively. The culture medium was filtered through two layers of cheesecloth, concentrated by ultrafiltration (Merck Millipore) and used for pull-down assay with MoCel10A and OsRMC. Purified His-tagged MoCel10A or OsRMC protein was incubated with a rice crude protein preparation or culture filtrates in binding buffer (sodium phosphate buffer [50 mM, pH 7.5] containing 150 mM NaCl) for 1 h at 4°C. The mixture was further incubated with His-resin for 1 h at 4°C. Proteins bound to His-resin were eluted using binding buffer containing 200 mM imidazole. Bound proteins were subjected to SDS-PAGE followed by silver staining. For CoIP assay, His-tagged protein and Flag-tagged protein were incubated for 1 h at 4°C. The mixture was further incubated with His-resin for 1 h at 4°C. The supernatant of this mixture, obtained by centrifugation at 2,400 *g*, was used as an unbound fraction. Proteins bound to His-resin were eluted using binding buffer containing 200 mM imidazole. Both fractions were subjected to SDS-PAGE followed by immunoblotting using anti-His and anti-Flag antibodies.

### Peptide sequence identification

Protein bands separated by SDS-PAGE were excised and digested with trypsin as described previously [47]. Peptide identification was performed as described previously [48]. Digested peptides were applied onto a Magic C18 AQ nano column (0.1 × 150 mm, MICHROM Bioresources, Inc.) in an ADVANCE UHPLC system (MICHROM Bioresources, Inc.) equilibrated with 0.1% formic acid (v/v) in acetonitrile and eluted using a linear gradient of 5–45% (v/v) acetonitrile at a flow rate of 500 nL/min. Mass analysis was performed using an LTQ Orbitrap XL mass spectrometer (Thermo Fisher Scientific) with Xcalibur software ver. 2.0.7 (Thermo Fisher Scientific). Peptides were identified using a MASCOT MS/MS ion search (http://www.matrixscience.com/home.html) in error tolerance mode (one amino acid substitution allowed) using the NCBI database. Search parameters were as follows: taxonomy, plants; max missed cleavages, 0; fixed modifications, carbamidomethyl; peptide tolerance, ± 5 ppm; fragment mass tolerance, ± 0.6 Da.

### Gel-permeation chromatography

MoCel10A-His, OsRMC-His, and the mixture of MoCel10A-His and OsRMC-His preincubated at 4°C for 30 min were applied on a Superdex G-75 column (GE Healthcare) equilibrated with sodium phosphate buffer (50 mM, pH 7.5) and 150 mM NaCl. Proteins were detected by immunoblot analysis using anti-His antibody. Fractions contain 3 mL of the eluate. Proteins, albumin (75 kDa), carbonic anhydrase (29 kDa), and aprotinin (6.5 kDa), were used as a standard marker.

### Determination of binding kinetics

Integrated intensity representing the binding kinetics of OsRMC to MoCel10A, MoCel6A, and MoCel10AΔCBM was determined on a BLItz instrument using BLItz Pro software (ForteBio) as described previously [49]. OsRMC-His (10 μL, 2.0 μM) was loaded onto a His-tag biosensor for 10 min. A baseline of binding response was determined by incubating the sensor with binding buffer (50 mM sodium phosphate buffer, pH 7.5, 150 mM NaCl) for 10 min. Association of MoCel10A, MoCel6A, and MoCel10AΔCBM proteins (10 μL, 2.0 μM) was measured for a duration of 5 min. Binding buffer was used to measure protein dissociation for a duration of 4 min. Time zero was defined as the starting point of protein association. Fold increase in integrated intensity was calculated by dividing each trajectory by the value at time zero.

### Binding of MoCel10A to cellulose

MoCel10A-His (2.0 μg) was preincubated in sodium phosphate buffer (100 mM, pH6.0) with a protein mixture (total 2.0 μg) of OsRMC-His (0–2.0 μg) and BSA (2.0–0 μg) for 30 min at 4°C. Cellulose (1 mg) was added, and the mixture was further incubated for 1 h at 4°C with vigorous agitation. The supernatant obtained by centrifugation at 13,000 *g* and proteins eluted from cellulose by boiling in SDS-PAGE sample buffer were used as fractions unbound and bound to cellulose, respectively.

### Effects of OsRMC on xylan hydrolytic activity of MoCel10A

A mixture (20 μL) containing MoCel10A-His (2.0 μg), sodium phosphate buffer (100 mM, pH 6.0), and OsRMC-His (0–2.0 μg) was incubated for 15 min at 4°C and further incubated with wheat coleoptile cell wall preparation for 30 min at 30°C. BSA was added to the mixture to adjust to a final protein content of 2.0 μg instead of OsRMC. Hydrolytic activity was determined by measuring absorbance at 640 nm after staining solubilized sugars from the cell wall preparation with 0.5% (w/v) anthrone in H_2_SO_4_. For hydrolytic activity towards water-soluble xylan, a reaction mixture (100 μL) containing MoCel10A-His (0.2 μg) preincubated with OsRMC (0–1.0 μg), water-soluble oat spelts xylan (1%, w/v), and sodium phosphate buffer (100 mM, pH 6.0) was incubated at 30°C. BSA was added to the mixture to adjust to a final protein content of 1.0 μg instead of OsRMC. The activity was determined by measuring the absorbance at 410 nm after treatment with *p*-hydroxybenzoic hydrazide-HCl. Data are means ± SD of independent three determinations.

### Extension assay of heat-inactivated wheat coleoptiles

Heat-inactivated wheat coleoptile segments were treated with MoCel10A-His (2.0 μg) or a mixture of MoCel10A-His and OsRMC-His (each 2.0 μg) in 200 μL of sodium phosphate buffer (100 mM, pH 6.0). Specimens treated with buffer were used as a control. Specimens were fixed between two clamps approximately 5 mm apart and loaded at a constant 200 mN for 3 min in an extensometer (TMA/SS6000, Seiko Instruments). Extension was automatically recorded by computer from time 0.5 s every 0.1 s for 1 min and every 1 s for the next 2 min. Strain was calculated as (*L*_*t*_-*L*_0.1_)/*L*_0.1_ (*L*_*t*_, length of coleoptiles at each time point; *L*_0.1_, length of segments at time 0.1). Data are means ± SD of five independent determinations.

### Subcellular localization of OsRMC-GFP

*Agrobacterium* carrying *OsRMC-GFP* and *OsRMCΔSP-GFP*, in which secretion signal peptide sequence was deleted, was infiltrated into *N*. *benthamiana*. The cells were plasmolyzed by infiltrating 50 mM sodium phosphate buffer (pH7.5) with 0.7 M mannitol and FM4-64 to *N*. *benthamiana* leaves. The leaves were observed on a Olympus Fluoview FV1000 confocal laser-scanning microscope (Olympus, Japan).

### Generation of transgenic rice plants

*OsRMC* DNA fragments, 311 bp from 456–766 of the ORF region and 433 bp from 784–1216 of the 3′-noncoding region, were cloned into pANDA vector for RNA interference of the *OsRMC* gene as described previously [50]. Gene introduction into rice (‘Moukoto’) was performed as described above. T_1_ *OsRMC*-knockdown and wild-type control progenies segregated from T_0_ heterozygous lines were used for *M*. *oryzae* infection assay. *OsRMC*-overexpressing rice (‘Hitomebore’) were generated by introducing p2K^+^ vector containing maize ubiquitin promoter and a *OsRMC* coding region DNA fused with HA-tag sequence (*OsRMC*-*HA*) at the C-terminus. Transgenic plants were selected by hygromycin resistance and protein accumulation of OsRMC-HA.

### Quantitative PCR (qPCR) and reverse-transcription PCR (qRT-PCR)

Rice leaves (‘Hitomebore’ and ‘Moukoto’) spray-inoculated with *M*. *oryzae* spores (0.3–1.5 X 10^6^; compatible strain Ken53-33 and incompatible strain TH69-8) in 0.01% Tween-20 (v/v) were used to determine levels of *OsRMC* gene expression as well as for quantification of the rice *ACTIN* gene (*Os03g61970*.*1*) and *M*. *oryzae actin* gene (*XM_003719823*.*1*) from genomic DNA. qPCR was conducted using a Quantitect SYBR Green PCR Kit (Qiagen) and specific DNA primers (S4 Table) in a StepOnePlus Real-Time PCR system (Applied Biosystems) using SYBR GreenER qPCR Super Mix (Invitrogen). Data are means ± SD of three independent determinations.

### Antifungal activity assay

Antifungal activity against *M*. *oryzae* was evaluated by measuring luciferase activity in transformed *M*. *oryzae* constitutively overexpressing luciferase under control of the MPG1 promoter [45]. *M*. *oryzae* grown on oatmeal plates was transferred to YG medium and cultured with protein additive for 30 h at 25°C. Cells were then sonicated, and the supernatant obtained after centrifugation at 22,000 *g* was used for analyzing luciferase activity in a Varioskan LUX (Themo Fischer Scientific). Antifungal activity against *F*. *oxysporum* was determined as described previously [25].

### Protein detection

Protein detection was performed by silver staining and immunoblot analysis using antibodies against peptide epitope-tags and anti-OsRMC after SDS-PAGE. Protein concentration was determined using a Bradford protein assay kit (Thermo Fischer Scientific) with bovine serum albumin (Sigma-Aldrich) as the standard. Proteins on a PolyVinylidene DiFluoride (PVDF) membrane were stained using CBB Stain One (Nacalai tesque).

### Phylogenetic tree reconstruction

Amino acid sequences of CRRSPs and CRKs from *O*. *sativa*, *A*. *thaliana*, *M*. *polymorpha*, and *Selaginella moellendorffii* were retrieved from the Rice Genome Annotation Project (http://rice.uga.edu/index.shtml), The Arabidopsis Information Resource (https://www.arabidopsis.org/), EnsemblPlants Marchantia polymorpha (https://plants.ensembl.org/Marchantia_polymorpha/Info/Index), and EnsemblPlants Selaginella moellendorffii (https://plants.ensembl.org/Selaginella_moellendorffii/Info/Index), respectively. Amino acid sequences of SiCBMIP (*S*. *italica*), AFP1 (*Z*. *mays*), and Gnk2 (*G*. *biloba*) were added to these sequences. DUF26-containing proteins were annotated using InterproScan with default options [51]. The amino acid sequences of annotated DUF26 domains were aligned using MAFFT [52] with the following method parameter set:—maxiterate 1000 -globalpair (S5 Table). A maximum-likelihood tree was reconstructed using IQ-TREE [53] with 1,000 bootstrap replicates calculated using UFBoot2 [54]. ModelFinder [55] was used for model selection; “WAG + R5” for S13 Fig and “WAG + I + G4” for Fig 8 were chosen as the best-fit models, according to BIC. Finally, the reconstructed tree was drawn using Iroki [56].

### Experimental design

Each experiment was performed on at least three biological replicates, and all of the experiments were conducted in two or more independent replications.

## Supporting information

S1 Table Amino acid sequence identities among four CBM1-binding proteins. (TIFF)

S2 Table Summary of the number of CBM families. (TIFF)

S3 Table Summary of binding ability of various DUF26-containing proteins to CBM1 and mannose. (TIFF)

S4 Table DNA primers used in this study. (TIFF)

S5 Table A list of amino acid sequences of DUF26 used for constructing phylogenetic tree. (PDF)

S1 Fig Peptide sequences of OsRMC detected by LC-MS/MS. A rice protein band detected by pull-down assay using MoCel10A-His was treated with trypsin, and the resulting peptide fragments were analyzed by LC-MS/MS. The 14 peptides identified are underlined. Methionine residues of peptides 12 and 14 were oxidized. CRR motifs are indicated in red.
(TIFF)

S2 Fig Expression of Flag-tagged MoCel10A and MoCel10AΔCBM in *Nicotiana benthamiana*. MoCel10A-Flag and MoCel10AΔCBM-Flag were expressed in 4 plants of *N*. *benthamiana*. The leaves were extracted with sodium phosphate buffer (50 mM, pH 7.5) containing 150 mM NaCl and used as Buffer-soluble fraction. The pellets were suspended in the same buffer containing 0.1% SDS and boiled. The supernatant was used as a SDS-soluble fraction. Prepared proteins were subjected to SDS-PAGE followed by immunoblot analysis using anti-Flag. MoCel10A-Flag was not recovered in the Buffer-soluble fraction.
(TIFF)

S3 Fig Gel-permeation chromatography of monomers and the complex of OsRMC and MoCel10A. MoCel10A-His, OsRMC-His, and a mixture of MoCel10A-His and OsRMC-His preincubated at 4°C for 30 min were separated on a Superdex G-75 column equilibrated with sodium phosphate buffer (50 mM, pH 7.5) and 150 mM NaCl. Proteins were detected by immunoblot analysis using anti-His antibody. Protein standard markers albumin (75 kDa), carbonic anhydrase (29 kDa), and aprotinin (6.5 kDa) were eluted in fractions 48, 60, and 72, respectively.
(TIFF)

S4 Fig Binding of OsRMC to CBM1-containing proteins MoCel6A and MoCDH. Crude protein preparations (20 μg) containing (A) Flag-tagged MoCel6A (MoCel6A-Flag) or (B) MoCDH (MoCDH-Flag) prepared from *M*. *oryzae* were incubated in sodium phosphate buffer (50 mM, pH 7.5) containing 150 mM NaCl with or without OsRMC-His (2.0 μg) for 1 h at 4°C before being further incubated with His-resin. Fractions unbound and bound to His-resin were subjected to SDS-PAGE followed by immunoblot analysis using anti-Flag antibody.
(TIFF)

S5 Fig Identification of *T*. *reesei* proteins bound to OsRMC. (A) Culture filtrate from *T*. *reesei* culture grown in induction medium with 0.5% (w/v) cellobiose was incubated with (+) or without (-) OsRMC-His and further incubated with His-resin. Fractions bound to His-resin were subjected to SDS-PAGE followed by silver staining. Arrowhead indicates OsRMC-His. Identification of proteins bound to OsRMC, indicated by arrows (a–c), was carried out by LC-MS/MS. Proteins labeled (a) and (b) correspond to GH7 cellobiohydrolase (XM_006969162) and GH6 cellobiohydrolase (XM_006962518), respectively. Proteins labeled (c) with negligible levels at the same position in the control (-) were GH5 endoglucanase (XM_006962521), GH7 cellobiohydrolase (XM_006965612), GH61 endoglucanase (XM_006961505), and GH15 carbohydrate esterase (XM_006969057). (B) Schematic structures of *T*. *reesei* proteins bound to OsRMC. All proteins possess CBM1 at the N- or C-terminal region connected to the catalytic core domain.
(TIFF)

S6 Fig Hydrolytic activity of MoCel10A towards water-soluble xylan. (A) Dependence of the hydrolytic activity of MoCel10A-His towards water-soluble oat spelt xylan on incubation time. (B) Effects of OsRMC on the hydrolytic activity of MoCel10A-His towards water-soluble xylan. MoCel10A-His (0.2 μg) was preincubated in sodium phosphate buffer (100 mM, pH 6.0) with OsRMC-His (0–1.0 μg) at 4°C for 30 min before further incubating with water-soluble oat spelt xylan at 30°C for 10 min. Data are means ± SD of three independent determinations.
(TIFF)

S7 Fig Fractionation of OsRMC proteins expressed in *N*. *benthamiana* leaves and rice suspension cells. (A) OsRMC-GFP expressed in *N*. *benthamiana* leaves was sequentially extracted using sodium phosphate buffer (100 mM, pH 6.0) containing 100 mM NaCl (buffer-soluble), or the same buffer containing 1% (v/v) Triton-X100 (Triton-X100-soluble) or 1% (w/v) SDS (SDS-soluble). Buffer-soluble protein from *N*. *benthamiana* leaves overexpressing GFP was used as a GFP marker. (B) Rice suspension cells overexpressing OsRMC-HA were separated into culture filtrate (extracellular) and cells. Proteins were extracted from cells using sodium phosphate buffer (100 mM, pH 6.0) containing 100 mM NaCl (buffer-soluble) or 1% (w/v) SDS (SDS-soluble). The prepared proteins (1.0 mg) were subjected to SDS-PAGE followed by immunoblot analysis using anti-GFP and anti-HA antibodies.
(TIFF)

S8 Fig Gene structure of *OsRMC* and DNA fragments used for the RNAi experiment. The *OsRMC* gene consists of one exon of 777 bp. DNA fragments of 311 bp in the ORF and 433 bp in the 3′-UTR region were used for RNAi experiments.
(TIFF)

S9 Fig RNAi-mediated knockdown of the *OsRMC* gene in rice. PCR amplification of the hygromycin transgene, OsRMC protein accumulation, and disease symptoms after *M*. *oryzae* inoculation of T_1_ progeny segregating for *OsRMC* knockdown and expression. (A) *OsRMC*-knockdown (kd) #1–1 and *OsRMC*-expressing control #1–1 lines; (B) *OsRMC*-knockdown (kd) #1–2 and *OsRMC*-expressing control #1–2 lines; (C) *OsRMC*-knockdown (kd) #2–1 and *OsRMC*-expressing control #2–1 lines; (D) *OsRMC*-knockdown (kd) #2–2 and *OsRMC*-expressing control #2–2 lines.
(TIFF)

S10 Fig Determination of qPCR conditions for evaluating the level of *M*. *oryzae* infection in rice plants. (A) Variable amounts (0.1–10 ng per 15 mL reaction mixture) of rice genomic DNA were used to amplify the rice *ACTIN* gene by qPCR. Ct values decreased linearly as the amount of rice genomic DNA increased. (B) Variable amounts (0.001–10 ng) of *M*. *oryzae* genomic DNA were mixed with rice genomic DNA to a final amount of 10 ng. The mixture was used for qPCR to amplify the *M*. *oryzae ACTIN* gene. Ct values decreased linearly as the amount of *M*. *oryzae* genomic DNA increased.
(TIFF)

S11 Fig Determination of luciferase assay conditions for evaluating growth of *M*. *oryzae*. Variable amounts (50–400 mg fresh weight) of luciferase-expressing *M*. *oryzae* were examined for luciferase activity. The average value for 50 mg fresh weight of *M*. *oryzae* was defined as the unit for the ratio. Activity increased linearly as the fresh weight increased up to 300 mg.
(TIFF)

S12 Fig Identification of CBM1-binding protein from *Setaria italica*. (A) Protein was extracted from *S*. *italica* leaves 4 days after *M*. *oryzae* inoculation using sodium phosphate buffer (50 mM, pH 7.5) containing 150 mM NaCl. Protein preparation was incubated with (+) or without (-) MoCel6A-His for 1 h at 4°C before being further incubated with His-resin. Fractions bound to His-resin were subjected to SDS-PAGE followed by silver staining. Arrowhead indicates added MoCel6A-His. Arrow indicates a candidate protein that interacts with MoCel6A-His. (B) The candidate protein was identified as a member of the CRRSPs by LC-MS/MS. Peptide sequences obtained are underlined. The methionine residue of peptide number 7 was oxidized. CRR motifs are indicated in red.
(TIFF)

S13 Fig Identification of *A*. *thaliana* CRRSP that interacts with CBM1. (A) Phylogenetic tree of DUF26 domains (DUF26-A and DUF26-B) from *A*. *thaliana* CRRSPs and CRKs along with OsRMC, reconstructed using amino acid sequences. Bar, 0.25 amino acid substitutions per site. AtCRRSPs assayed for binding to MoCel10A-His are indicated in red. (B) Flag-tagged Arabidopsis CRRSPs were expressed in *N*. *benthamiana* leaves and extracted using sodium phosphate buffer (50 mM, pH 7.5) containing 150 mM NaCl. Prepared proteins were incubated with MoCel10A-His for 1 h at 4°C before further incubating with His-resin. Fractions unbound and bound to His-resin were subjected to SDS-PAGE followed by immunoblot analysis using anti-Flag. A protein encoded by *AT3G22060*.*1* bound to CBM1.
(TIFF)

S14 Fig Identification of *O*. *sativa* CRK that interacts with CBM1. (A) DUF26 domains from *O*. *sativa* CRKs were expressed in *N*. *benthamiana* leaves and extracted using sodium phosphate buffer (50 mM, pH 7.5) containing 150 mM NaCl. Prepared proteins were incubated with MoCel10A-His for 1 h at 4°C before being further incubated with His-resin. Fractions unbound and bound to His-resin were subjected to SDS-PAGE followed by immunoblot analysis using anti-Flag. DUF26 domains encoded by *LOC_Os07g35580*.*1* bound CBM1.
(TIFF)

S15 Fig Binding assay of various DUF26-containing proteins to mannose. Flag-tagged DUF26 domains from *O*. *sativa* CRKs, CRRSP encoded by *LOC_Os04g25650*.*1*, SiCBMIP, and Arabidopsis CRRSPs were expressed in *N*. *benthamiana* leaves and assayed for binding to mannose-agarose. Fractions unbound and bound to mannose-agarose were subjected to immunoblot analysis using an anti-Flag antibody.
(TIFF)

S16 Fig Amino acid sequence alignment among OsRMC, LOC_Os04g25650.1, and OsCBMIP-K. (TIFF)

S17 Fig Binding assay of AFP1 to CBM1 and mannose. Flag-tagged AFP1 (AFP1-Flag) and OsRMC (OsRMC-Flag) expressed in *N*. *benthamiana* were assayed for binding to MoCel10A-His (A) and mannose-agarose (B). Fractions unbound and bound to His-resin or mannose-agarose were subjected to immunoblot analysis using an anti-Flag antibody.
(TIFF)
