# Supplementary figures and images for: Rice apoplastic CBM1-interacting protein counters blast pathogen invasion by binding conserved carbohydrate binding module 1 motif of fungal proteins
Source: PLoS Pathog. 2022 Sep 29;18(9):e1010792. doi: 10.1371/journal.ppat.1010792 (PMC9521807; doi:10.1371/journal.ppat.1010792)

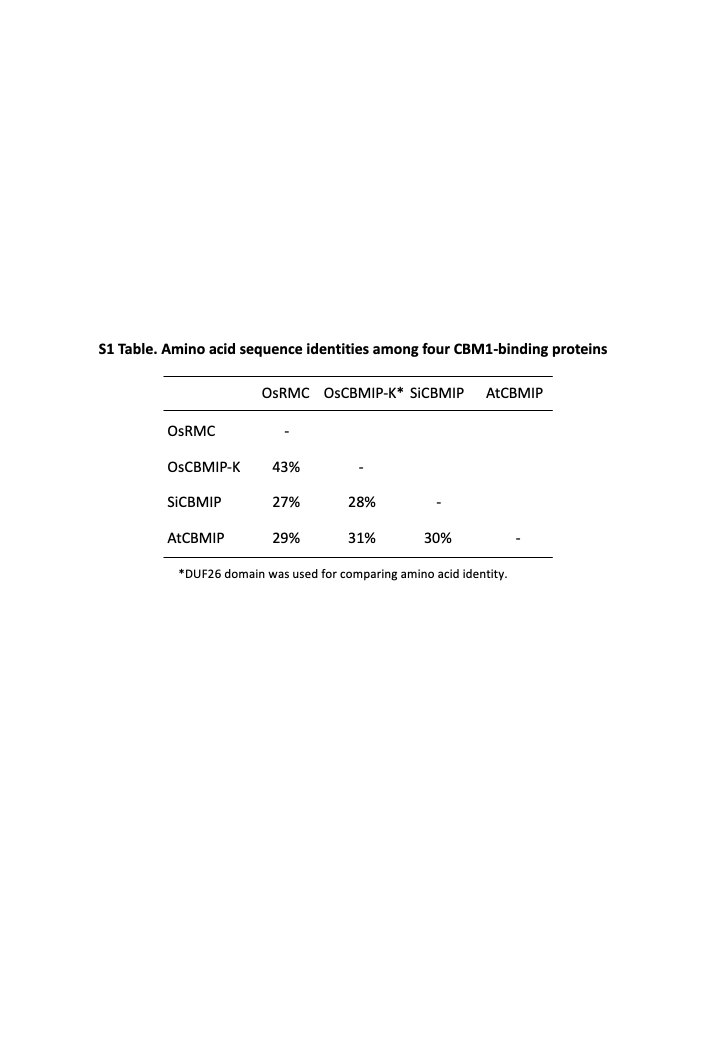

Supplement: S1 Table — (TIFF) [file ppat.1010792.s001.tiff]

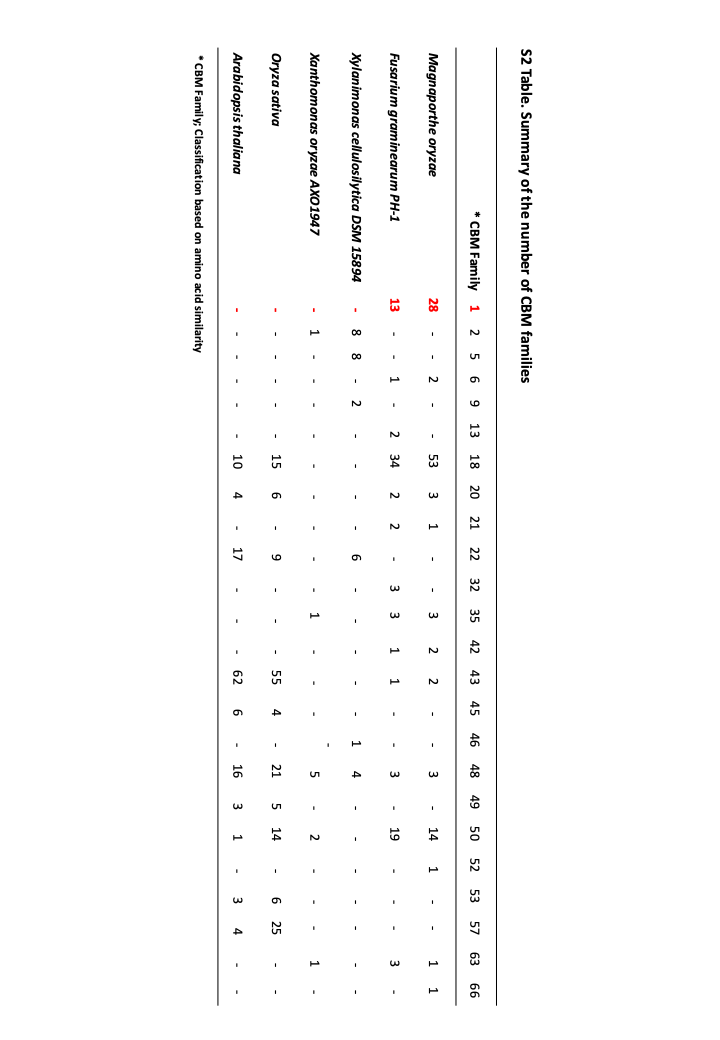

Supplement: S2 Table — (TIFF) [file ppat.1010792.s002.tiff]

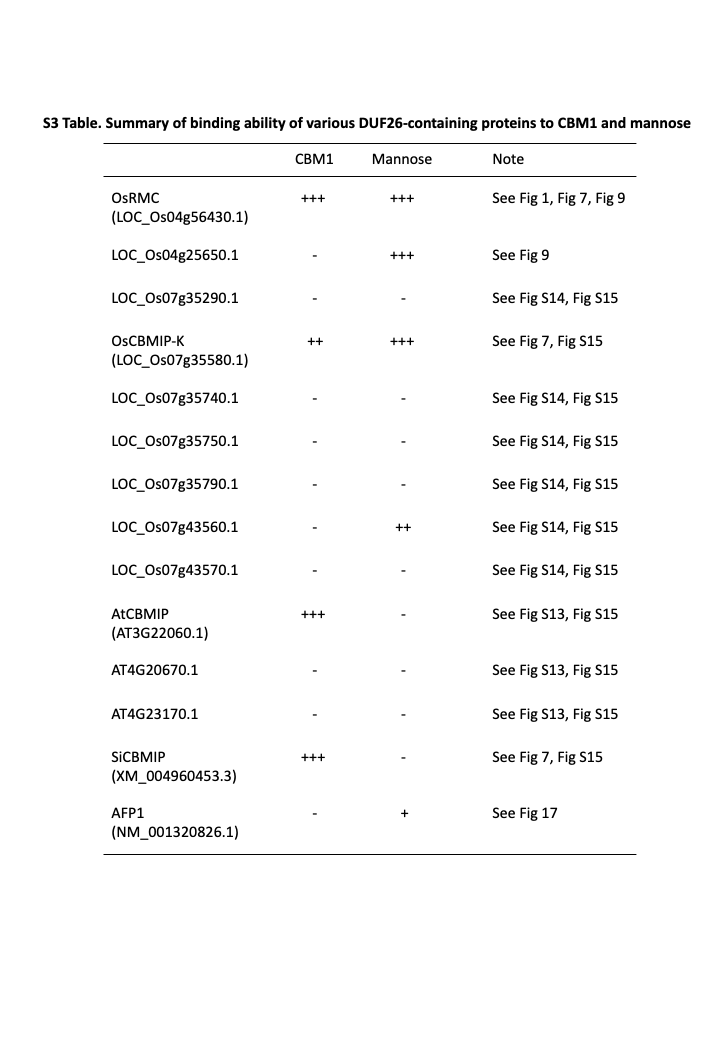

Supplement: S3 Table — (TIFF) [file ppat.1010792.s003.tiff]

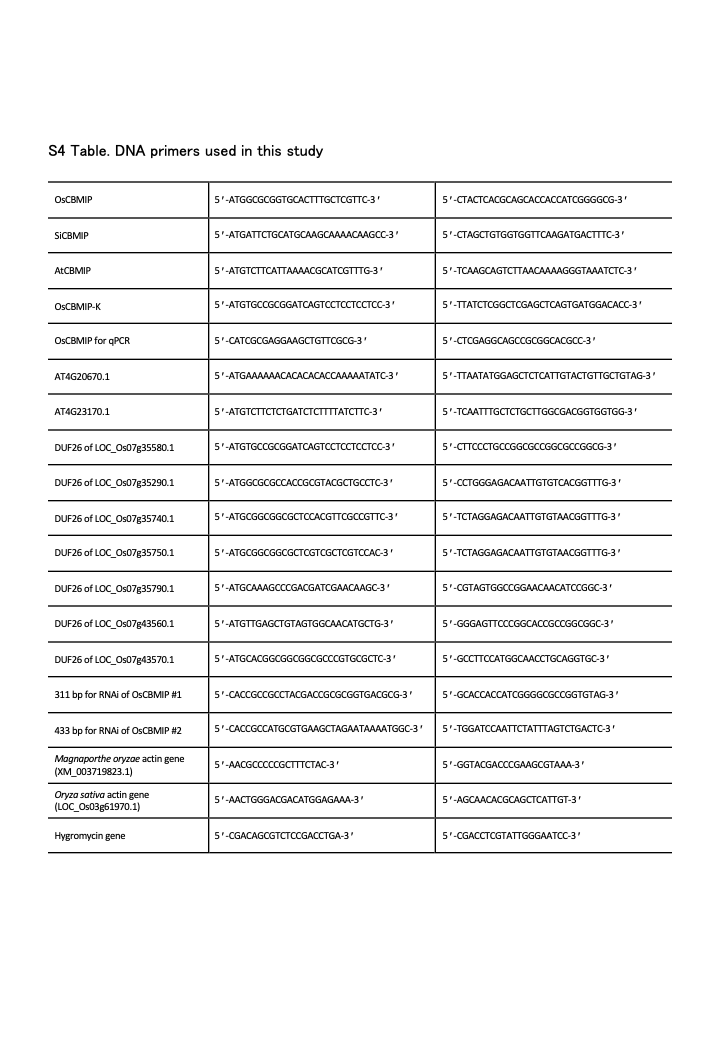

Supplement: S4 Table — (TIFF) [file ppat.1010792.s004.tiff]

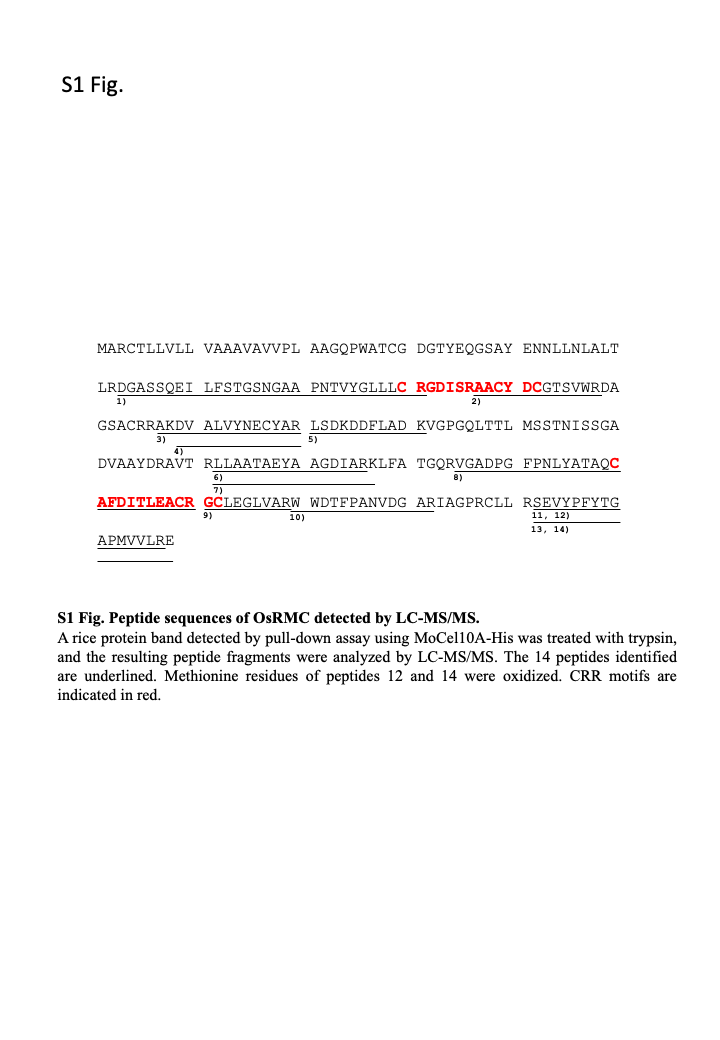

Supplement: S1 Fig — A rice protein band detected by pull-down assay using MoCel10A-His was treated with trypsin, and the resulting peptide fragments were analyzed by LC-MS/MS. The 14 peptides identified are underlined. Methionine residues of peptides 12 and 14 were oxidized. CRR motifs are indicated in red. (TIFF) [file ppat.1010792.s006.tiff]

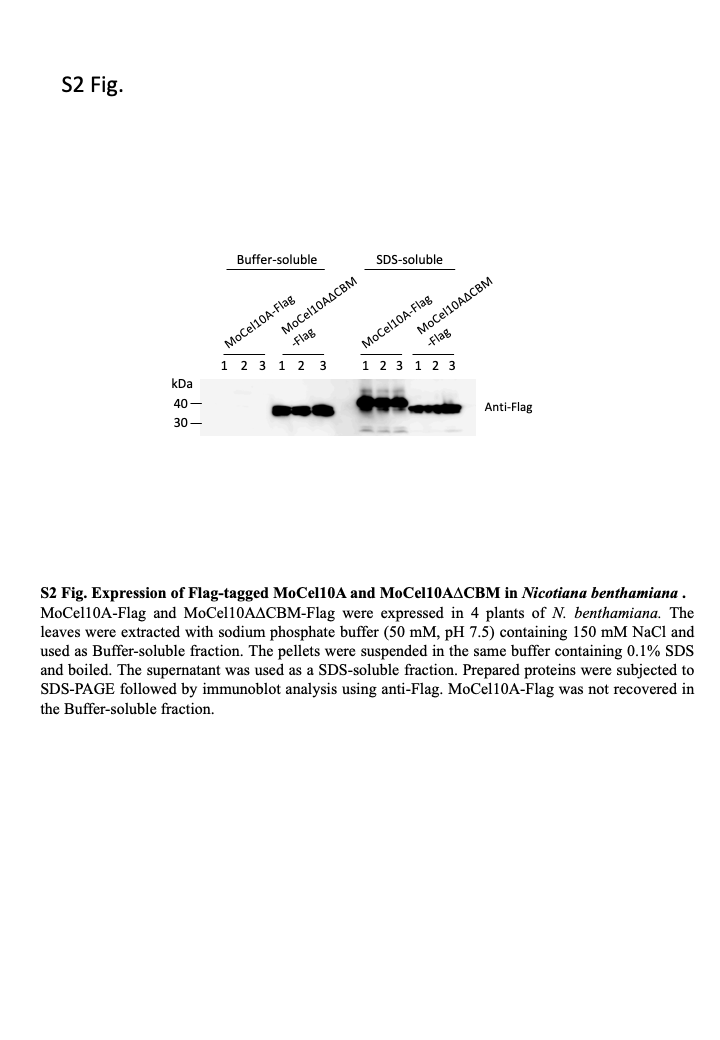

Supplement: S2 Fig — MoCel10A-Flag and MoCel10AΔCBM-Flag were expressed in 4 plants of N. benthamiana. The leaves were extracted with sodium phosphate buffer (50 mM, pH 7.5) containing 150 mM NaCl and used as Buffer-soluble fraction. The pellets were suspended in the same buffer containing 0.1% SDS and boiled. The supernatant was used as a SDS-soluble fraction. Prepared proteins were subjected to SDS-PAGE followed by immunoblot analysis using anti-Flag. MoCel10A-Flag was not recovered in the Buffer-soluble fraction. (TIFF) [file ppat.1010792.s007.tiff]

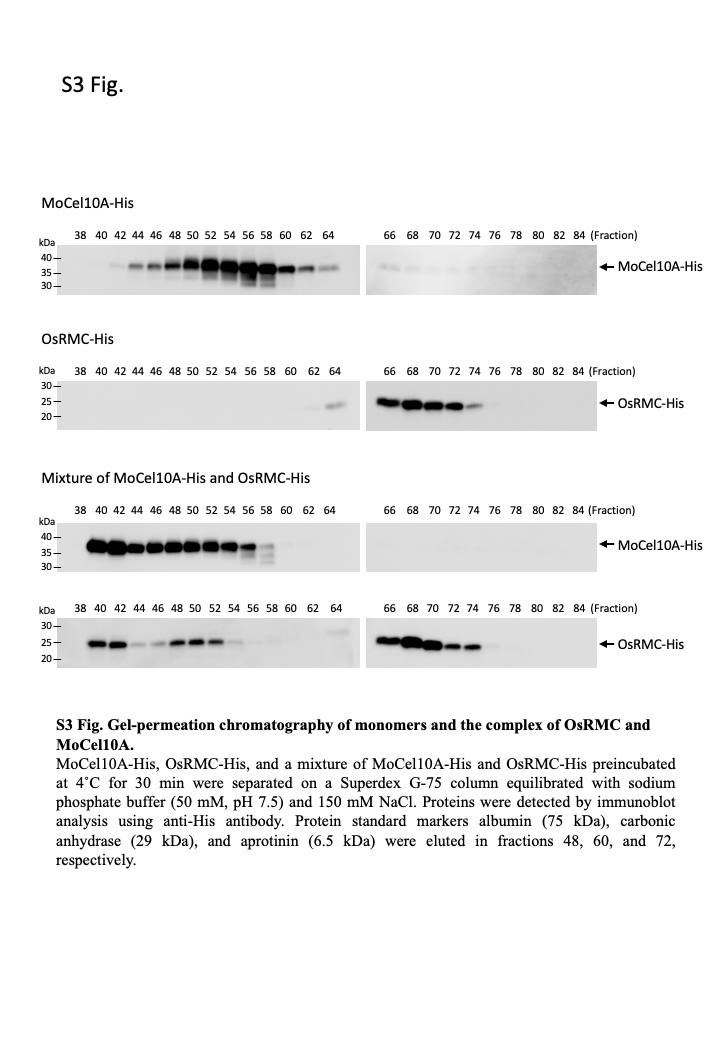

Supplement: S3 Fig — MoCel10A-His, OsRMC-His, and a mixture of MoCel10A-His and OsRMC-His preincubated at 4°C for 30 min were separated on a Superdex G-75 column equilibrated with sodium phosphate buffer (50 mM, pH 7.5) and 150 mM NaCl. Proteins were detected by immunoblot analysis using anti-His antibody. Protein standard markers albumin (75 kDa), carbonic anhydrase (29 kDa), and aprotinin (6.5 kDa) were eluted in fractions 48, 60, and 72, respectively. (TIFF) [file ppat.1010792.s008.tiff]

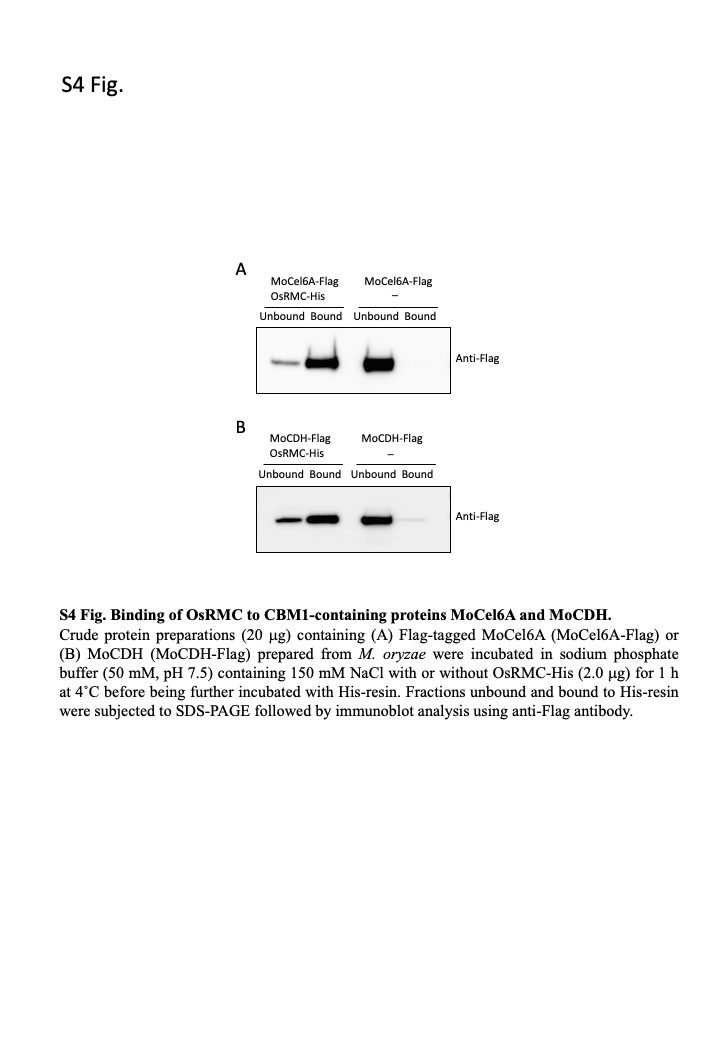

Supplement: S4 Fig — Crude protein preparations (20 μg) containing (A) Flag-tagged MoCel6A (MoCel6A-Flag) or (B) MoCDH (MoCDH-Flag) prepared from M. oryzae were incubated in sodium phosphate buffer (50 mM, pH 7.5) containing 150 mM NaCl with or without OsRMC-His (2.0 μg) for 1 h at 4°C before being further incubated with His-resin. Fractions unbound and bound to His-resin were subjected to SDS-PAGE followed by immunoblot analysis using anti-Flag antibody. (TIFF) [file ppat.1010792.s009.tiff]

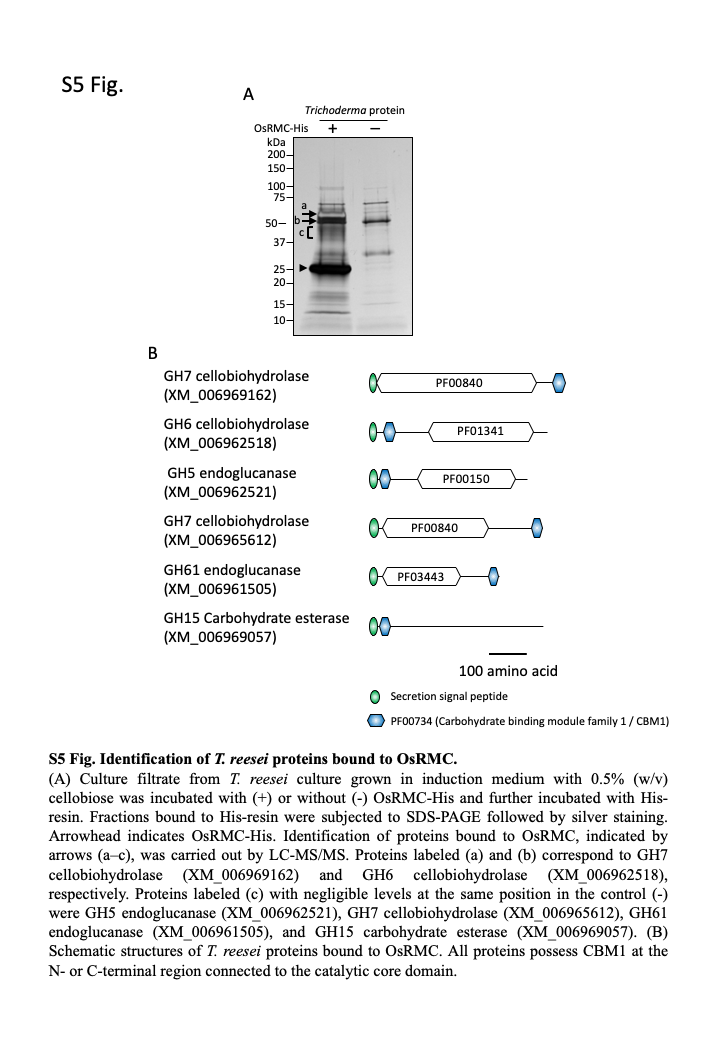

Supplement: S5 Fig — (A) Culture filtrate from T. reesei culture grown in induction medium with 0.5% (w/v) cellobiose was incubated with (+) or without (-) OsRMC-His and further incubated with His-resin. Fractions bound to His-resin were subjected to SDS-PAGE followed by silver staining. Arrowhead indicates OsRMC-His. Identification of proteins bound to OsRMC, indicated by arrows (a–c), was carried out by LC-MS/MS. Proteins labeled (a) and (b) correspond to GH7 cellobiohydrolase (XM_006969162) and GH6 cellobiohydrolase (XM_006962518), respectively. Proteins labeled (c) with negligible levels at the same position in the control (-) were GH5 endoglucanase (XM_006962521), GH7 cellobiohydrolase (XM_006965612), GH61 endoglucanase (XM_006961505), and GH15 carbohydrate esterase (XM_006969057). (B) Schematic structures of T. reesei proteins bound to OsRMC. All proteins possess CBM1 at the N- or C-terminal region connected to the catalytic core domain. (TIFF) [file ppat.1010792.s010.tiff]

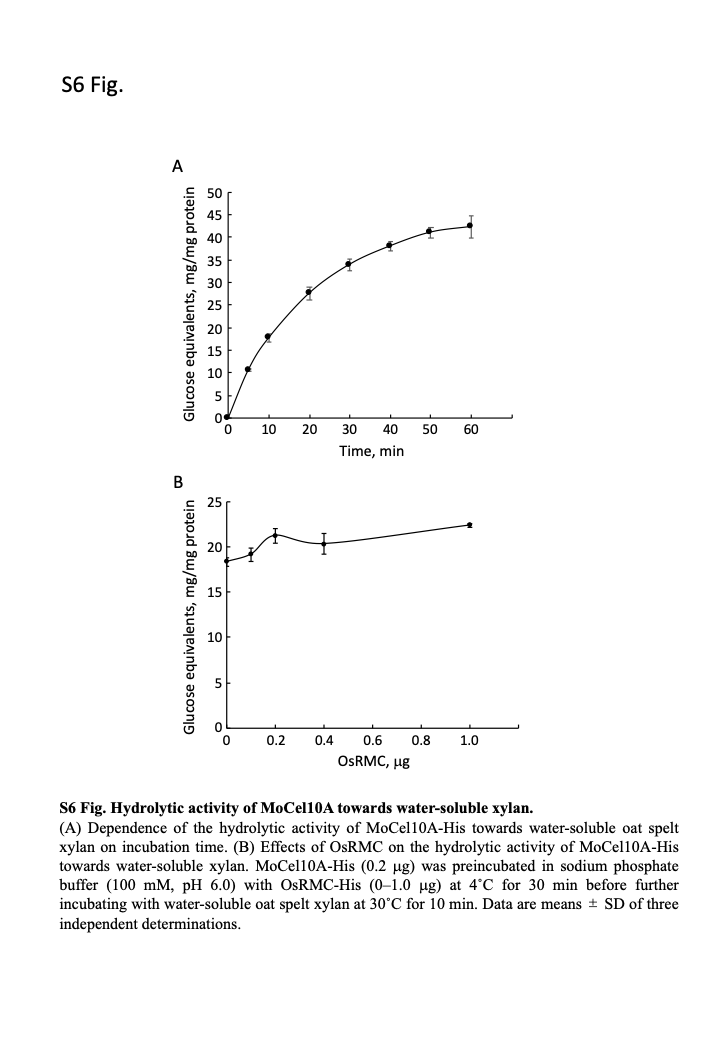

Supplement: S6 Fig — (A) Dependence of the hydrolytic activity of MoCel10A-His towards water-soluble oat spelt xylan on incubation time. (B) Effects of OsRMC on the hydrolytic activity of MoCel10A-His towards water-soluble xylan. MoCel10A-His (0.2 μg) was preincubated in sodium phosphate buffer (100 mM, pH 6.0) with OsRMC-His (0–1.0 μg) at 4°C for 30 min before further incubating with water-soluble oat spelt xylan at 30°C for 10 min. Data are means ± SD of three independent determinations. (TIFF) [file ppat.1010792.s011.tiff]

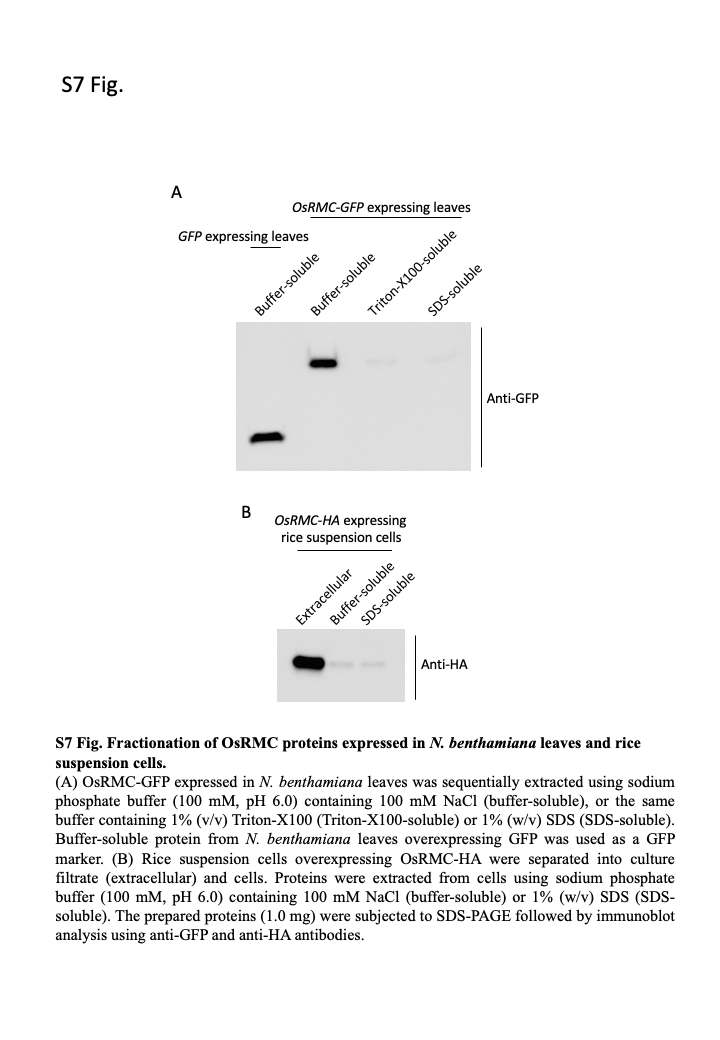

Supplement: S7 Fig — (A) OsRMC-GFP expressed in N. benthamiana leaves was sequentially extracted using sodium phosphate buffer (100 mM, pH 6.0) containing 100 mM NaCl (buffer-soluble), or the same buffer containing 1% (v/v) Triton-X100 (Triton-X100-soluble) or 1% (w/v) SDS (SDS-soluble). Buffer-soluble protein from N. benthamiana leaves overexpressing GFP was used as a GFP marker. (B) Rice suspension cells overexpressing OsRMC-HA were separated into culture filtrate (extracellular) and cells. Proteins were extracted from cells using sodium phosphate buffer (100 mM, pH 6.0) containing 100 mM NaCl (buffer-soluble) or 1% (w/v) SDS (SDS-soluble). The prepared proteins (1.0 mg) were subjected to SDS-PAGE followed by immunoblot analysis using anti-GFP and anti-HA antibodies. (TIFF) [file ppat.1010792.s012.tiff]

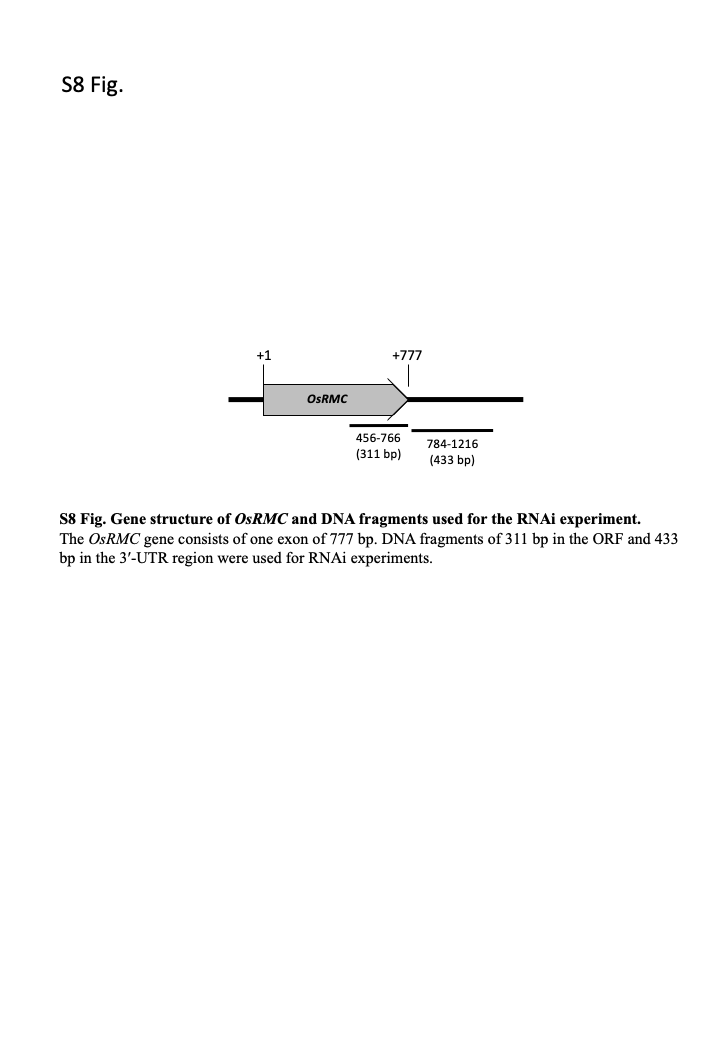

Supplement: S8 Fig — The OsRMC gene consists of one exon of 777 bp. DNA fragments of 311 bp in the ORF and 433 bp in the 3′-UTR region were used for RNAi experiments. (TIFF) [file ppat.1010792.s013.tiff]

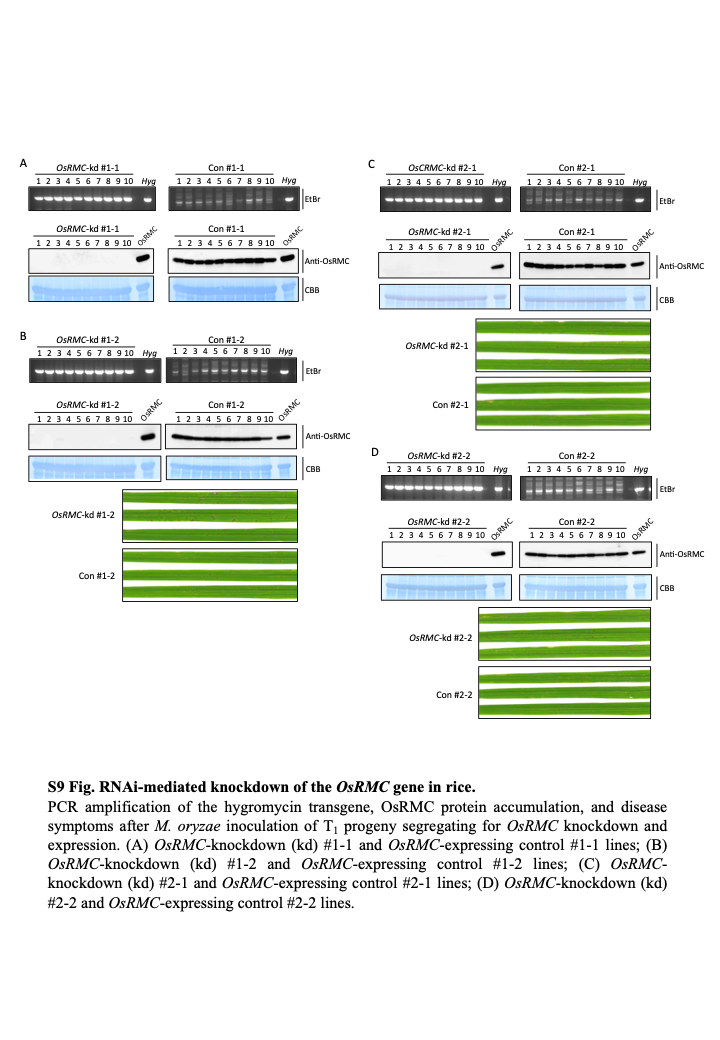

Supplement: S9 Fig — PCR amplification of the hygromycin transgene, OsRMC protein accumulation, and disease symptoms after M. oryzae inoculation of T1 progeny segregating for OsRMC knockdown and expression. (A) OsRMC-knockdown (kd) #1–1 and OsRMC-expressing control #1–1 lines; (B) OsRMC-knockdown (kd) #1–2 and OsRMC-expressing control #1–2 lines; (C) OsRMC-knockdown (kd) #2–1 and OsRMC-expressing control #2–1 lines; (D) OsRMC-knockdown (kd) #2–2 and OsRMC-expressing control #2–2 lines. (TIFF) [file ppat.1010792.s014.tiff]

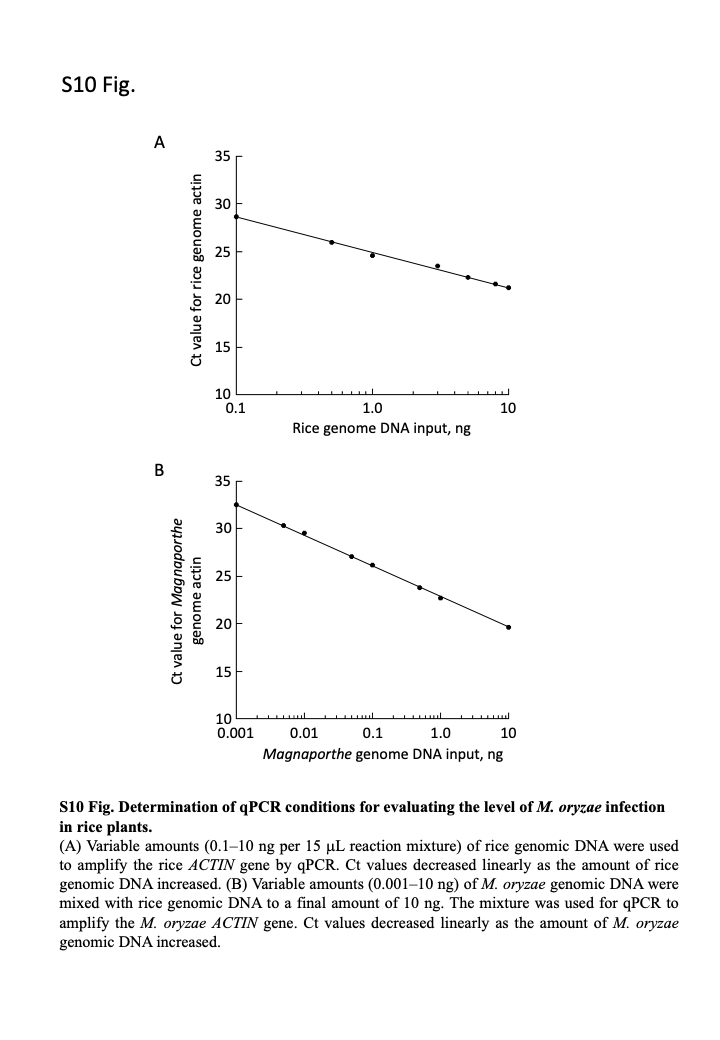

Supplement: S10 Fig — (A) Variable amounts (0.1–10 ng per 15 mL reaction mixture) of rice genomic DNA were used to amplify the rice ACTIN gene by qPCR. Ct values decreased linearly as the amount of rice genomic DNA increased. (B) Variable amounts (0.001–10 ng) of M. oryzae genomic DNA were mixed with rice genomic DNA to a final amount of 10 ng. The mixture was used for qPCR to amplify the M. oryzae ACTIN gene. Ct values decreased linearly as the amount of M. oryzae genomic DNA increased. (TIFF) [file ppat.1010792.s015.tiff]

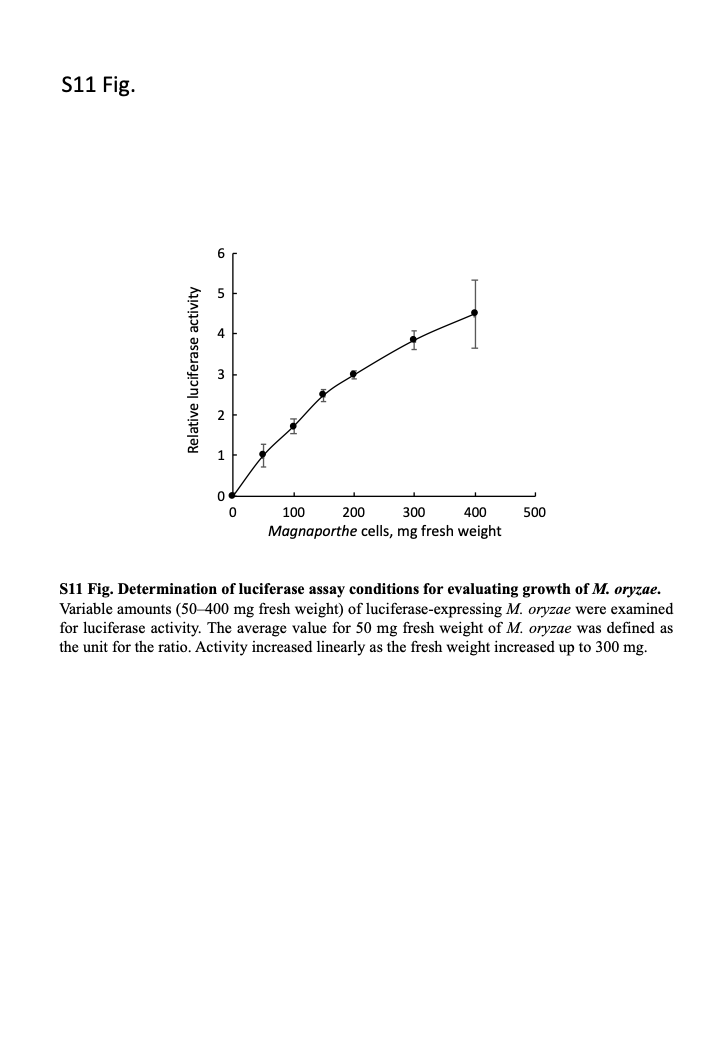

Supplement: S11 Fig — Variable amounts (50–400 mg fresh weight) of luciferase-expressing M. oryzae were examined for luciferase activity. The average value for 50 mg fresh weight of M. oryzae was defined as the unit for the ratio. Activity increased linearly as the fresh weight increased up to 300 mg. (TIFF) [file ppat.1010792.s016.tiff]

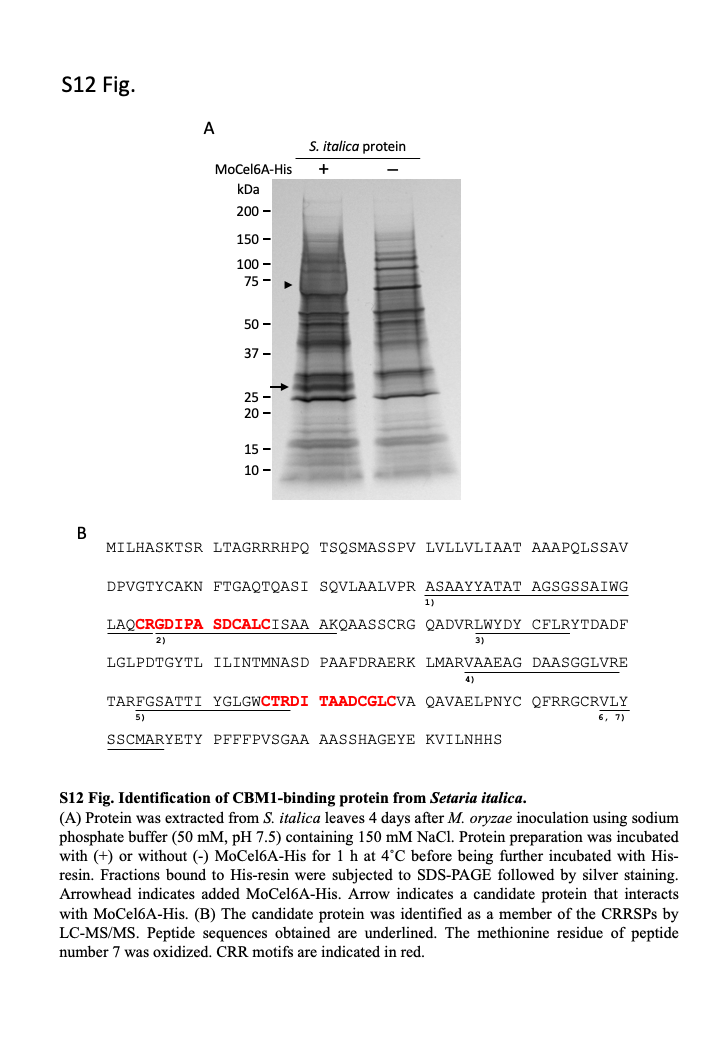

Supplement: S12 Fig — (A) Protein was extracted from S. italica leaves 4 days after M. oryzae inoculation using sodium phosphate buffer (50 mM, pH 7.5) containing 150 mM NaCl. Protein preparation was incubated with (+) or without (-) MoCel6A-His for 1 h at 4°C before being further incubated with His-resin. Fractions bound to His-resin were subjected to SDS-PAGE followed by silver staining. Arrowhead indicates added MoCel6A-His. Arrow indicates a candidate protein that interacts with MoCel6A-His. (B) The candidate protein was identified as a member of the CRRSPs by LC-MS/MS. Peptide sequences obtained are underlined. The methionine residue of peptide number 7 was oxidized. CRR motifs are indicated in red. (TIFF) [file ppat.1010792.s017.tiff]

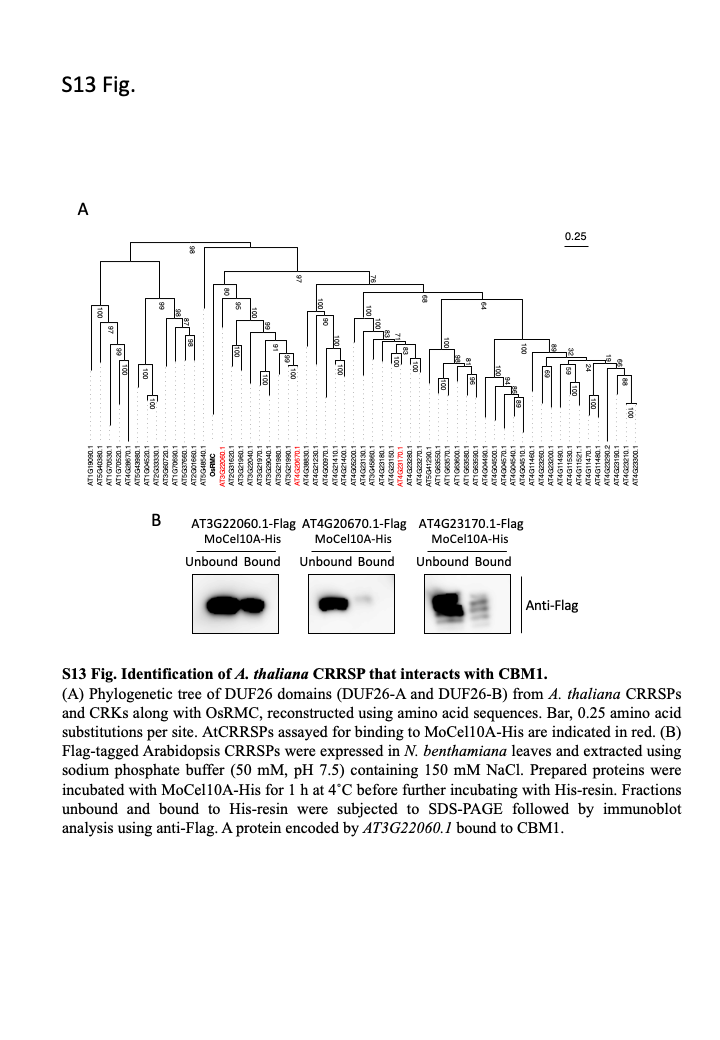

Supplement: S13 Fig — (A) Phylogenetic tree of DUF26 domains (DUF26-A and DUF26-B) from A. thaliana CRRSPs and CRKs along with OsRMC, reconstructed using amino acid sequences. Bar, 0.25 amino acid substitutions per site. AtCRRSPs assayed for binding to MoCel10A-His are indicated in red. (B) Flag-tagged Arabidopsis CRRSPs were expressed in N. benthamiana leaves and extracted using sodium phosphate buffer (50 mM, pH 7.5) containing 150 mM NaCl. Prepared proteins were incubated with MoCel10A-His for 1 h at 4°C before further incubating with His-resin. Fractions unbound and bound to His-resin were subjected to SDS-PAGE followed by immunoblot analysis using anti-Flag. A protein encoded by AT3G22060.1 bound to CBM1. (TIFF) [file ppat.1010792.s018.tiff]

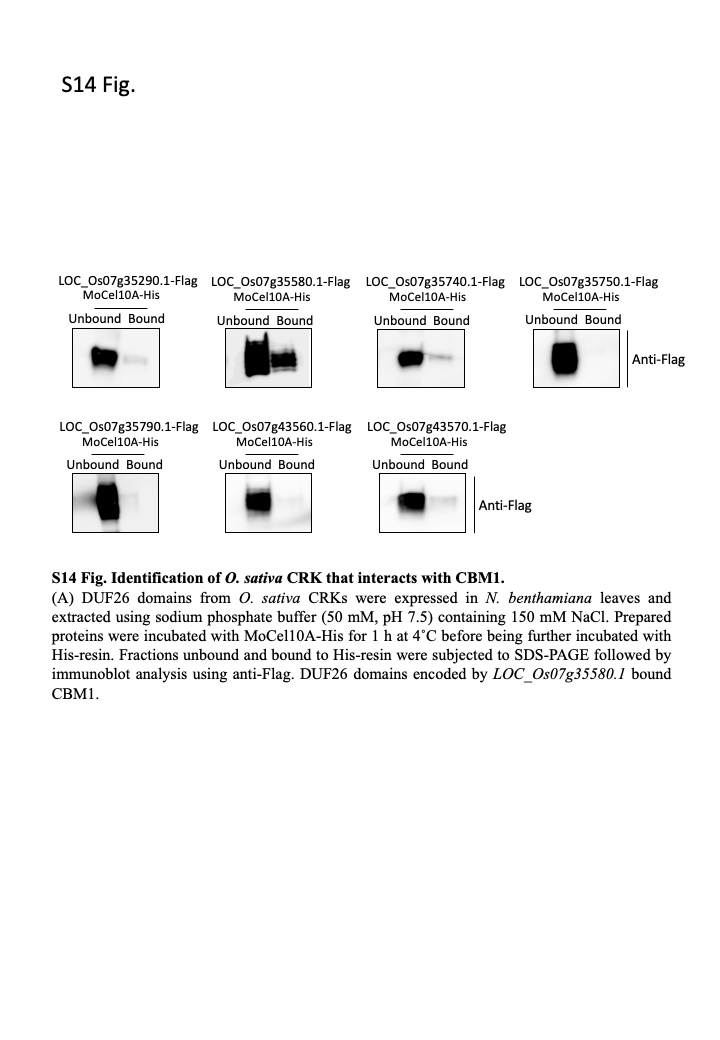

Supplement: S14 Fig — (A) DUF26 domains from O. sativa CRKs were expressed in N. benthamiana leaves and extracted using sodium phosphate buffer (50 mM, pH 7.5) containing 150 mM NaCl. Prepared proteins were incubated with MoCel10A-His for 1 h at 4°C before being further incubated with His-resin. Fractions unbound and bound to His-resin were subjected to SDS-PAGE followed by immunoblot analysis using anti-Flag. DUF26 domains encoded by LOC_Os07g35580.1 bound CBM1. (TIFF) [file ppat.1010792.s019.tiff]

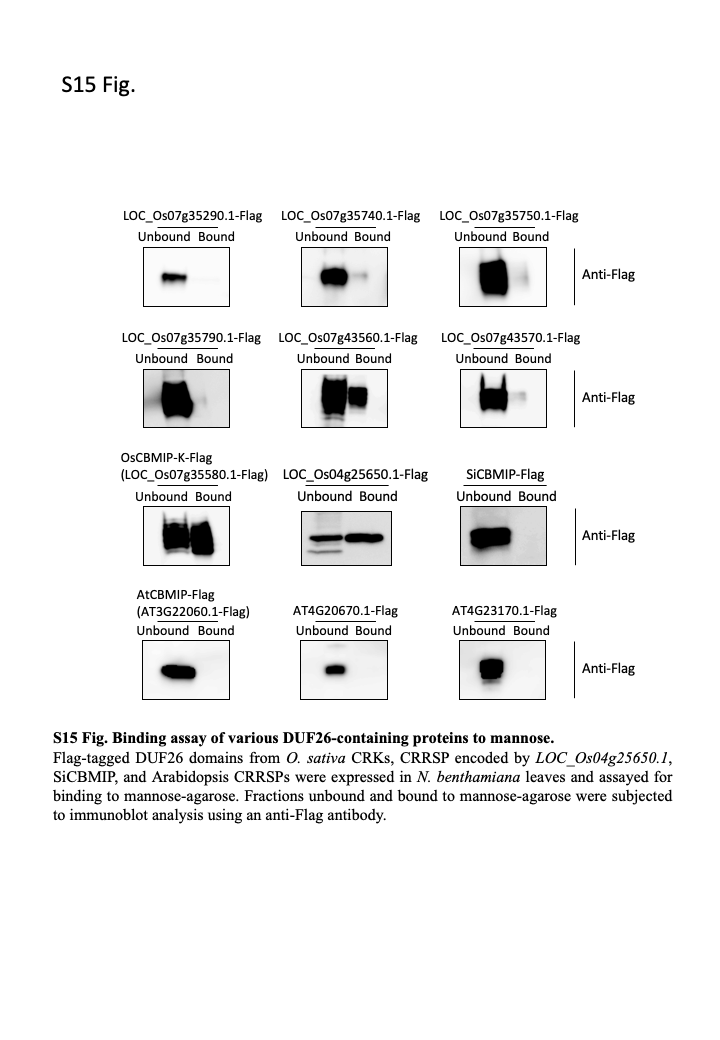

Supplement: S15 Fig — Flag-tagged DUF26 domains from O. sativa CRKs, CRRSP encoded by LOC_Os04g25650.1, SiCBMIP, and Arabidopsis CRRSPs were expressed in N. benthamiana leaves and assayed for binding to mannose-agarose. Fractions unbound and bound to mannose-agarose were subjected to immunoblot analysis using an anti-Flag antibody. (TIFF) [file ppat.1010792.s020.tiff]

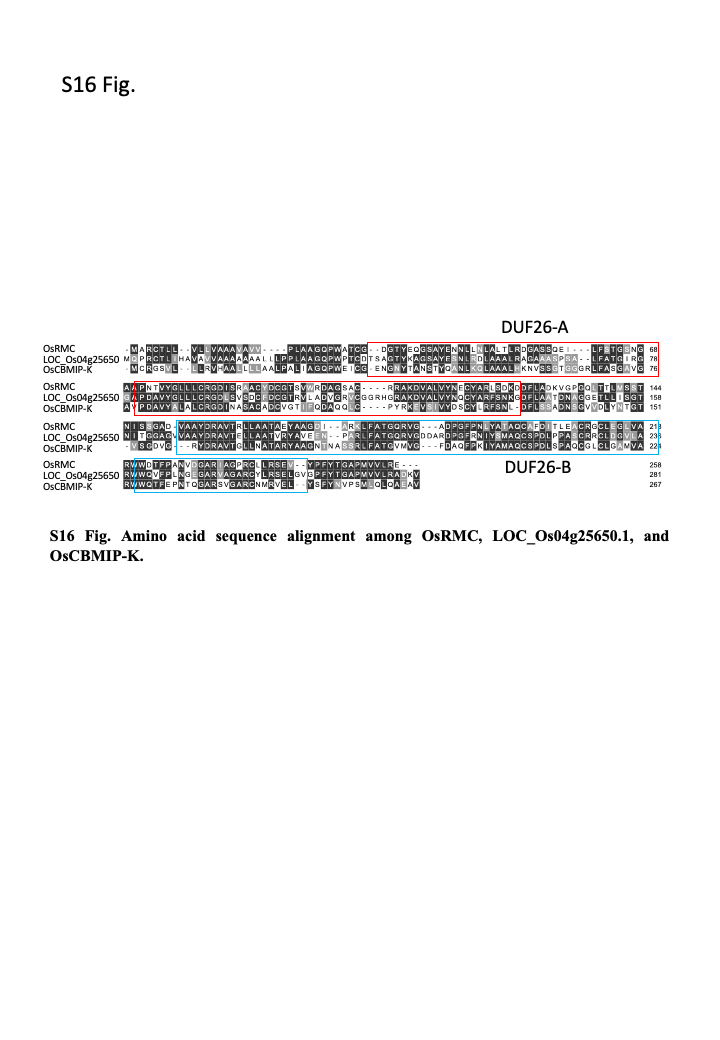

Supplement: S16 Fig — (TIFF) [file ppat.1010792.s021.tiff]

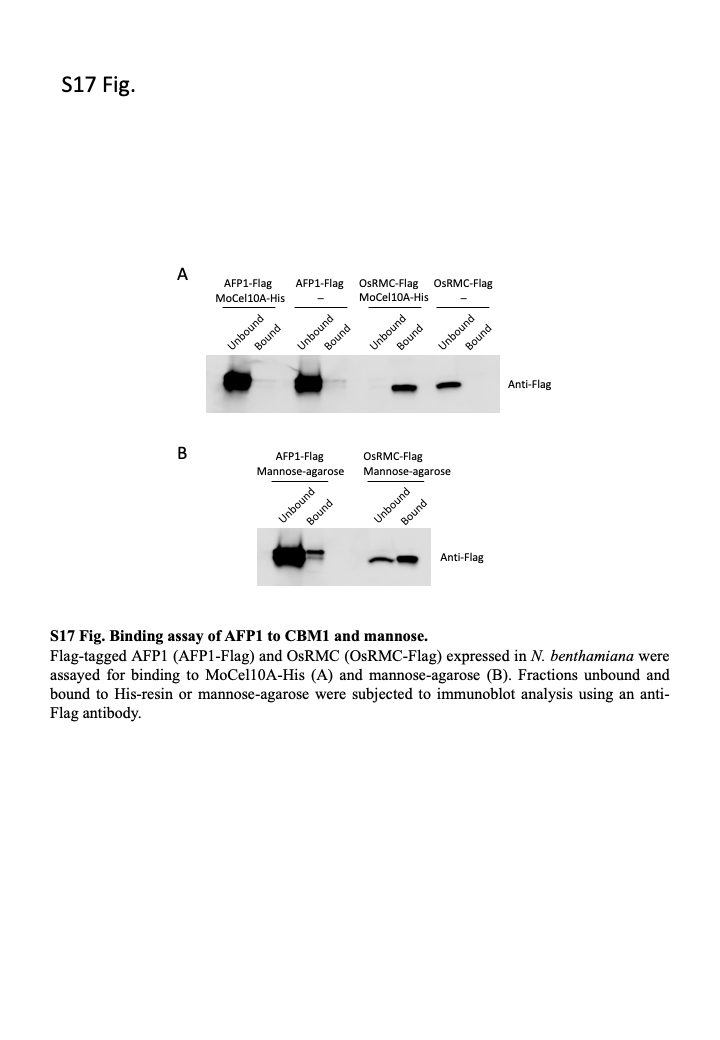

Supplement: S17 Fig — Flag-tagged AFP1 (AFP1-Flag) and OsRMC (OsRMC-Flag) expressed in N. benthamiana were assayed for binding to MoCel10A-His (A) and mannose-agarose (B). Fractions unbound and bound to His-resin or mannose-agarose were subjected to immunoblot analysis using an anti-Flag antibody. (TIFF) [file ppat.1010792.s022.tiff]
