# Supplementary material for: Rice apoplastic CBM1-interacting protein counters blast pathogen invasion by binding conserved carbohydrate binding module 1 motif of fungal proteins
Source: PLoS Pathog. 2022 Sep 29;18(9):e1010792. doi: 10.1371/journal.ppat.1010792 (PMC9521807; doi:10.1371/journal.ppat.1010792)
Supplement: S5 Table — (PDF) [file ppat.1010792.s005.pdf]

S5 Table. A list of amino acid sequences of DUF26 used for constructing phylogenetic tree

Single DUF26 sequences

| | |
|---|---|
| AT3G04370.1 | NYSNQNLFLRAQALSSFLRKLESESSRSKFLKTLVGNEKHAVSGWFQCREDYPSEICHKCVGDLREISSRSCGNATSARIHLRGCHLIYK |
| AT3G22030.1 | GNVNSFNKKTTEFLYKLIGKADRLDVDGINFLYYAAGEMRLGKQTLFAMVQCAKDILSCKDCLEWSIKELSKCCDGKQGARVVGTICNLRYELY |
| AT4G20530.1 | KCSNTQGKYKQGSAFEKNLNLVLSTITSIGNFRDGFRYTEEGEDPNNVFVMFQCRGDSYWSKCPPCISTAVSGLRRRCPRNKGAIIWYDQCLLKIS |
| AT4G20540.1 | KCSNTQGKYKQGSAFEKNLNLVLSTITSIGNFRDGFRYTEEGEDPNNVFVMFQCRGDSYWSKCPPCISTAVSGLRRRCPRNKGAIIWYDQCLLKIS |
| AT4G20600.1 | NNMSDRGLFNKETSALLEKLAYKASDRNNLDGKQLVLYAAGEKRIGTKKVYAMVQCTKDLIFTKCFECLEGILRKFPQCCDGKRGGRVFGTSCNFR |
| AT4G20630.1 | KCSNTQGKYKQGSAFEKNLNLVLSTITSIGNFRDGFRYTEEGEDPNNVFVMFQCRGDSYWSKCPPCISTAVSGLRRRCPRNKGAIIWYDQCLLKIS |
| AT4G20650.1 | KCSNTQGKYKQGSAFEKNLNLVLSTITSIGNFRDGFRYTEEGEDPNNVFVMFQCRGDSYWSKCPPCISTAVSGLRRRCPRNKGAIIWYDQCLLKIS |
| AT5G41280.1 | CNDSSGNFTRNTTYNTNLNTLLSTLSNQSSFANYYNLTTGLGSDTVHGMFLCIGDVNRTTCNACVKNATIEIAKNCTNHREAIIYYFSCMVRYSD |
| EFJ04200 | NVNSAMVVLSSHGSSTTVTSGDGKSRVFGLRECRRDMSAEQCNTCMAVATKNLHRETCYARFDTFL |
| EFJ10327 | SFSPGSAFERNLDAALQSVISSSRSSPSAALGASPDVAYARGECYNNLSPQDCVLCLGMANQTIRGQAPRTIGARLFSNSSDYSCYLRYENY |
| EFJ12758 | NTSLGSPFQQNWLDVYQVLLDHAPQAVSSEFSHGQSPNTVSGFALCIKGSNCKGCLQTIKANLDRAAPLSKGARLCMKTSTDVCYMRYEAY |
| EFJ12964 | ESSEDSPFEKNLASAYESLLSPSQESPRQAQAGDDPDTVYGYAACFEGNCEDCLATCKNYFTQVAPHAVGARLCLKSGSDACYLRYENY |
| EFJ13641 | RTYARGSVYGSNLNLLFDRLITTTGYDHVGSGSNRVYGFSECYGSPNCRSCLLAAVNSIRSAAPRAIGARICKALCYLRYENY |
| EFJ13924 | NTSLGSPFQQNWLDVYQVLLDHAPQAVSSEFSHGQSPNTVSGFALCIKGSNCKGCLQTIKANLDRAAPLSKGARLCMKTSTDVCYMRYEAY |
| EFJ15639 | STPYATNQNRLLDILKDTGSDSFIYTSVGDAPYIAYGRAECYGNCGNCLVEARNTIKNAVPQAVGARLCTDDCYLRFENY |
| EFJ15855 | NTTEGSPFQKNVETVLNFFYEHNPPNGFATCLGYGDFPNTVYGHTGCYNMSDQPSCSECIKKAYQGIQQNAPYTLGGTYWINETEIRCRLRYENY |
| EFJ16456 | NVNSAMVVLSSHGSSTTVTSGDGKSRVFGLRECRRDMSAEQCNTCMAVATKNLHRETCYARFDTFL |
| EFJ18708 | KTTEGSPFQKNVETVLKFFYEHNPPNGFATCLGYGDFPNTVYGHTGCYNMSDQPSCSECIKRAYQGIQQYALYALGGTYWINETESRCRLRYENY |
| EFJ22561 | ESSEDSPFEKNLASAYESLLSPSQESPRQAQVGDDPDTVYGYATCFEGNCEDCLATCKNYFTQVAPHAVGARLCLKSGSDACYLRYENY |
| EFJ22969 | CSDESIPTGSTYWTSLNQTLTDLIQNTAYANNMTYSVKNGGDDVSAYGQAQCFNMVGNPGMCKSCVEDLVRRAWRECGDAIAGMLMLKDKCTIRYQD |
| EFJ28936 | RTYARGSVYGSNLNLLFDRLITTTGYDHVGSGSNRVYGFSECYGSSNCRSCLLAAVNSIRSAAPRAIGARICKALCYLRYENY |
| EFJ30128 | RKTTQGSQFQKNVETVIQFLYEHNPPNGFASCLGYGDFPDTAYGHTGCYNMSDQTSCASCIQQTYSEFQQLAPLAVGGVLWTNDTSNRCRIRFENY |
| EFJ30567 | CSDESIPTGSTYWTSLNQTLTDLIQNTAYANNMTYSVKNGGDDVSAYGQAQCFNMVGNPGMCKSCVEDLVRRAWRECGDAIAAMLMLKDKCTIRYQD |
| EFJ32980 | NTTEGSPFQKNVETVLNFFYEHNPPNGFATCLGYGYFPNTVYGHTGCYNMSDQPSCSECIKKAYQGIQQNAPYTLGGTYWINETEIRCRLRYENY |
| EFJ33692 | RNTQPGSAFQRNLSNVYRRIINGATFRQGSISYNAGVDPDRVYGFSACFGGFTAPGDCVQCLVDIRANFDSVAPLAVGARMCSRNGERTCYLRYENY |

| | |
|---|---|
| EFJ33923 | LYSFGDPFGKNLGLVLDDLLGTTPGRDFKDYTAISPRPSRFVYGHAKCRHALSAGECGVCLKGVAAALADQCGNAVGGRAMFVDCFMRYEAY |
| EFJ37042 | RNTQPGSAFQRNLSNVYRRIINGATFRQGSISYNAGVDPDRVYGFSACFGGFTAPGDCVQCLVDIRANFDSVAPLAVGARMCSKNGERTCYMRYENY |
| EFJ37434 | LYSFGDPFGKNLGLVLDDLLGTTPRRDFKDYTAISPRPFRFVYGHAKCRHALSAGECGVCLKGVAAALADQCGNAVGGRAMFVDCFMRYEAY |
| Gnk2 | CNTQKIPSGSPFNRNLRAMLADLKQNTAFSGYDYKTSRAGSGGAPTAYGRATCKQSISQSDCTACLSNLVNRIFSICNNAIGARVQLVDCFIQYEQ |
| LOC_Os01g36500.1 | CGASNYTADSMYRLNLDGMSASLFPEGAGGSGGGIFVRGSSGADPDKVYAVALCRGDVDDAPACSSCFDAAFRRAMQLCPRSKDATIYYDECLLRFSD |
| LOC_Os01g38910.1 | CSLSGGRYGQNTTYEDNLKALAARLVGVARVSNFASHTVGSAPDAAYGIALCRGDYTGDECANGLRKAFENAVENRLFCDRFRDATIYYDQYMLRFS |
| LOC_Os01g56720.1 | GRVVAQCAAGVAPADCVQCLEGAAREMPRCFREARREEQGEGVGIVVSDDCVLRFD |
| LOC_Os01g56840.1 | TGSNGTAFRANLLTLLASLPDQAAPTGFASMQAGAGGRAPGGDDDDRAFARGSCLGDSTPSQCRDCLAAAVIDVAEGCGADTRRAGAWLSGCYLAYAD |
| LOC_Os03g19840.1 | NGKTRRRSINSVVSDLVAKAASNGGFATSSAGKGNNVFYGLAQCRGDVSASDCKACLVEAANYTLSFCHYASDSRMWYDYCFMRYKN |
| LOC_Os07g35190.1 | YAANSTYEANLRYLAATLPAKVMNGSSSSSVDVLAGERPNLIAASASCNSSSSEYHDCGACVAEAFRCARRLCPYSRHAVVHLGGGACSVRYY |
| LOC_Os10g10540.1 | CTSTAAGNYTQDGAYAANLGRLLAMLPNETVSKNGGFFNGTVGNGTATVYGLAMCAADFSRADCMDCLVAAGISAGGVVKRCPGSTTVSAMFDQCLLRYSD |
| LOC_Os12g41510.1 | CSTTGNYTVGNQFEKNLDQLLSTLATAATDDGWFNTSSVGTGTAYQVFGLIMCHADYNATECKKCLAGAPAGIKQVCPGSRTVKANYDACLLRYSD |
| PTQ30062 | RNGTAYQHNVRSVLSKLMAKVQSRPLFDVWDEASDAYDNSETAYGVAGCNTVTPEECRYCLQYIVTNLQQWCPKDPLSAKIVLKDCYLHYDRY |
| PTQ32071 | AVSACIDSLTEEPMDKKTASAEVKTPDKVVYCSMDCKQNLTLQECQACLQTAKLLRENYCPRAVSASSVIGICSLMHET |

## DUF26−A sequences

| | |
|---|---|
| AFP1 | CSGSSYAGSSKAVANINSVLADLVASASSTGGYATSTAGKGNNSIIYGLAQCRGDVSASDCASCLADAAKQLPSTCSYSSDARIWYDYCFMRYEN |
| AT1G04520.1 | QQFSDPSGLYSQALSAMFGSLVSQSTKTRFYKTTTGTSTTTITGLFQCRGDLSNHDCYNCVSRLPVLSDKLCGKTIASRVQLSGCYLLYE |
| AT1G19090.1 | SSITDVSPPIYVFLQCREDLSVSDCRHCFNESRLELERKCSGSGGRIHSDRCFLRFDD |
| AT1G61750.1 | CYTSGNYTPNSSYKSNLDTLISVLDSQSSNKGFYSYASGSSPTTTVYGSYLCRGDISSSTCETCISRASKNVFIWCPVQKEAIIWYEECFLRYSS |
| AT1G63550.1 | CNPTNNFTQTSSYETNRDTLLASLRESSSLGHYSNATEGLSPDTVHGMFLCRGDITTASCVDCVQTATTEIASNCTLNKRAVIYYDECMVRYSN |
| AT1G63570.1 | CSVDSFTQTSSYETNRNILLTTLSLTSSLVHYLNATIGLSPDTVYGMFLCRGDINTTSCSDCVQTAAIEIATNCTLNKRAFIYYDECMVRYSN |
| AT1G63580.1 | PDTYQSNRNTVLSTLRNHSSLGSYYFNATAGLSPNTVYGMFLCIGNISKTSCSNCVHSATLEMDKSCESHDTSFMFSDECMVRYSD |
| AT1G63590.1 | CTQFDNVTRTSSYLSNRDTVLSTLRNRSSIGSYSNATAGLSPNTIYGMFLCRGDLNRTSCSDCVNATTLEIYKSCFYRKSALVISNECIVRYSN |
| AT1G63600.1 | CNQFSDNFTQTSTYETNRETVLSSLRLRSSLGSYSNATAGISPDTVRGMFLCRGDISETSCSDCVQTATLEISRNCTYQKEAFIFYEECMVRYSD |
| AT1G70520.1 | ETAYVPNFVATMEKISTQVQTSGFGVALTGTGPDANYGLAQCYGDLPLNDCVLCYAEARTMLPQCYPQNGGRIFLDGCFMRAENY |

| | |
|---|---|
| AT1G70530.1 | CNNRTTTPQQRSLFVTNFLAAMDAVSPLVEAKGYGQVVNGTGNLTVYAYGECIKDLDKKDCDLCFAQIKAKVPRCLPFQKGTRGGQVFSDGCYIRYDDY |
| AT1G70690.1 | CSPAKFSPSSGYETNLNSLLSSFVTSTAQTRYANFTVPTGKPEPTVTVYGIYQCRGDLDPTACSTCVSSAVAQVGALCSNSYSGFLQMENCLIRYDN |
| AT2G01660.1 | CSQEKYFPGSPYESNVNSLLTSFVSSASLYTYNNFTTNGISGDSSSVYGLYQCRGDLSSGSGDCARCVARAVSRLGSLCAMASGGALQLEGCFVKYDN |
| AT2G31620.1 | CINSEGKYKAKNSYESRLKDHLDSMSNILDYGFIHGVGGADSSTYYIKAQCRGDASESKCRSCLFTAFSGILRRCPNNRGRIIWYDNCFLYIS |
| AT2G33330.1 | PSGLYSQALSAMYGLLVTQSTKTRFYKTTTGTTSQTSVTGLFQCRGDLSNNDCYNCVSRLPVLSGKLCGKTIAARVQLSGCYLLYE |
| AT3G21960.1 | CINGEGTFKSGSPYEKEIKQLIDFLSSFIKDYSFVHGVSGIGPDDINVKFQCRGDTLQAKCRSCLATAFSEIRSKCPNNKGRIIWYDNCHLDL |
| AT3G21970.1 | CNNTQGRYTHGSTFEKNLNQVLHNISNLDLRYGYAYVSNVVAYKVSKDPNIVFVLLQCRGDSFGSKCHSCLSTAVSGLRERCPGNRGATIWYDQCLLEIS |
| AT3G21980.1 | KCNNTEGKYSHGSAFEKYINLALRAIDSDNYLNGFAYIERGEDPNKVFVMYQCRGDSYGSKCKSCISAAIS |
| AT3G21990.1 | KCSNTQGKYKQGSAFEKNLNLVLSTITSIGNFRDGFRYTEEGEDPNNVFVMFQCRGDSYWSKCPPCISTAVSGLRRRCPRNKGAIIWYDQCLLKIS |
| AT3G22040.1 | CRVGQGKYHPGSRYEKDFDSLTSFVAANKFIDGFVHSSNSDGPNSTTIIFQCRGDSYKSNCRTCYDTALAGFRKRCPRNKGGIIWYDQCFLDVS |
| AT3G22060.1 | CSDIEGSFTSKSLYESNLNNLFSQLSYKVPSTGFAASSTGNTPNNVNGLALCRGDASSSDCRSCLETAIPELRQRCPNNKAGIVWYDNCLVKYSS |
| AT3G29040.1 | CNNTQGTYTRGSTFEKNLNQVIRNISHLHLRYGYTYNSNVEAYEVSKDPNIVFVLLQCRGDSYGSKCHSCLHTAFSGLRERCRGNKGAIIWYDQCVLEIS |
| AT3G45860.1 | SSILTYSRNSTYFTNLKTLLSSLSSRNASYSTGFQTATAGQAPDRVTGLFLCRGDVSQEVCRNCVAFSVKETLYWCPYNKEVVLYYDECMLRYSH |
| AT3G60720.1 | CSPEKYTPNTPFESNRDTFLSSVVTSSSDASFNSFAVGNDSSSSSSSSAVFGLYQCRDDLRSSDCSKCIQTSVDQITLICPYSYGASLQLEGCFLRYET |
| AT4G00970.1 | CLSQQSNFAKSSQFSKNLDSLVSSIPSLKSNTYNFYSLSVGSISDQERVEAIGICNRVVNRVDCLNCIAQAAVNLTTMYCPQHRGAYVRATKCMFRYSD |
| AT4G04490.1 | EDFSPNTSYVENLESLLPSLASNVIRERGFYNVSLDGVYALALCRKHYEVQACRRCVDRASRTLLTQCRGKTEAYHWDSENDANVSCLVRYSN |
| AT4G04500.1 | NYGVSRTYLFSSLPSNVVSNGGFYNASFGRDSKNNRVHVVALCRRGYEKQACKTCLEHVIEDTKSKCPRQKESFSWVTDEFDDVSCSLRYTN |
| AT4G04510.1 | SFPTNSSYQKNRDSLFSTLSDKVTTNGGFYNASLDGVHVVGLCRRDYDRQGCINCVEESIRQIKTSCSNRVQSFHCNSDDRERVSCLVRT |
| AT4G04540.1 | SFFNGNSSYAQNRRDLFSTLPNKVVTNGGFYNSSLGKSPNIVHAVALCGRGYEQQACIRCVDSAIQGILTTTSCLNRVDSFTWDKDEEDNVSCLVSTS |
| AT4G04570.1 | FNGNSSTFAQNRQKLFPTLADKVIINDGFYNASLGQDPDKVYALVSCARGYDQDACYNCVQSLTQNTLTDCRSRRDSFIWGGNDDVTCLVRSSN |
| AT4G05200.1 | CPNTTTYSRNSSYLTNLRTVLSSLSSPNAAYASLFDNAAAGEENDSNRVYGVFLCRGDVSAEICRDCVAFAANETLQRCPREKVAVIWYDECMVRYSNQ |
| AT4G11460.1 | CSEKFGTFTPGGTFDKNRRIILSSLPSEVTAQDGFYNASIGTDPDQLYAMGMCIPGAKQKLCRDCIMDVTRQLIQTCPNQTAAIHWSGGGKTVCMARYYN |
| AT4G11470.1 | VFFRPNGNYDTNRRLVLSTLASNVSSQNNRFYNVSVGEGAGRIYALGLCIPGSDPRVCSDCIQLASQGLLQTCPNQTDSFYWTGDNADKTLCFVRYSN |
| AT4G11480.1 | FFRPNGTYDTNRHLILSNLASNVSSRDGYYNGSVGEGPDRIYALGLCIPGTDPKVCDDCMQIASTGILQNCPNQTDSYDWRSQKTLCFVRYSN |
| AT4G11490.1 | CNETGYFEPWKTYDTNRRQILTSLASKVVDHYGFYNSSIGKVPDEVHVMGMCIDGTEPTVCSDCLKVAADQLQENCPNQTEAYTWTPHKTLCFARYSN |

| | |
|---|---|
| AT4G11521.1 | YFKPNGTYDLNRRRILSSLASKVTAHNGFYSSTIGQNPNQMFIISMCIPGTKPERCSDCIKGSTDGLLRSCPNQTVGYVWPDCCMVRYSN |
| AT4G11530.1 | FFKPNSTYDLNRRQILSTLSSNVTSHNGFFNSKFGQAPNRVFINGMCIPGTKPETCSDCIKGASDKISESCPNKTDAYTWPDCCMVRYSN |
| AT4G20550.1 | KCSNTQGKYKQGSAFEKNLNLVLSTITSIGNFRDGFRYTEEGEDPNNVFVMFQCRGDSYWSKCPPCISTAVSGLRRRCPRNKGAIIWYDQCLLKIS |
| AT4G20560.1 | KCSNTQGKYKQGSAFEKNLNLVLSTITSIGNFRDGFRYTEEGEDPNNVFVMFQCRGDSYWSKCPPCISTAVSGLRRRCPRNKGAIIWYDQCLLKIS |
| AT4G20570.1 | KCSNTQGKYKQGSAFEKNLNLVLSTITSIGNFRDGFRYTEEGEDPNNVFVMFQCRGDSYWSKCPPCISTAVSGLRRRCPRNKGAIIWYDQCLLKIS |
| AT4G20580.1 | KCSNTQGKYKQGSAFEKNLNLVLSTITSIGNFRDGFRYTEEGEDPNNVFVMFQCRGDSYWSKCPPCISTAVSGLRRRCPRNKGAIIWYDQCLLKIS |
| AT4G20590.1 | KCSNTQGKYKQGSAFEKNLNLVLSTITSIGNFRDGFRYTEEGEDPNNVFVMFQCRGDSYWSKCPPCISTAVSGLRRRCPRNKGAIIWYDQCLLKIS |
| AT4G20600.1 | KCSNTQGKYKQGSAFEKNLNLVLSTITSIGNFRDGFRYTEEGEDPNNVFVMFQCRGDSYWSKCPPCISTAVSGLRRRCPRNKGAIIWYDQCLLKIS |
| AT4G20610.1 | KCSNTQGKYKQGSAFEKNLNLVLSTITSIGNFRDGFRYTEEGEDPNNVFVMFQCRGDSYWSKCPPCISTAVSGLRRRCPRNKGAIIWYDQCLLKIS |
| AT4G20620.1 | KCSNTQGKYKQGSAFEKNLNLVLSTITSIGNFRDGFRYTEEGEDPNNVFVMFQCRGDSYWSKCPPCISTAVSGLRRRCPRNKGAIIWYDQCLLKIS |
| AT4G20640.1 | KCSNTQGKYKQGSAFEKNLNLVLSTITSIGNFRDGFRYTEEGEDPNNVFVMFQCRGDSYWSKCPPCISTAVSGLRRRCPRNKGAIIWYDQCLLKIS |
| AT4G20670.1 | KCSNTQGKYKQGSAFEKNLNLVLSTITSIGNFRDGFRYTEEGEDPNNVFVMFQCRGDSYWSKCPPCISTAVSGLRRRCPRNKGAIIWYDQCLLKIS |
| AT4G21230.1 | GNFTSNTSYSLNLNRLISSLPDLTPTINGFYNISINGEVNAIALCRGDVKPNQDCISCITTAAKQLVESCPNIIEANIWLEKCMFRYT |
| AT4G21400.1 | CVASGGNFTANSSFAGNLNGLVSSLSSLTSKPYGFYNLSSGDSSGERAYAIGLCRREVKRDDCLSCIQIAARNLIEQCPLTNQAVVWYTHCMFRYSN |
| AT4G21410.1 | CVDRGNFTANSTFAGNLNRLVSSLSSLKSQAYGFYNLSSGDSSGERAYAIGLCRREVKRDDCVSCIQTAARNLTKQCPLTKQAVVWYTHCMFRYSN |
| AT4G23130.1 | CTNRISRNSIYFSNLQTLLTSLSSNNAYFSLGSHSLTKGQNSDMVFGLYLCKGDLSPESCRECVIFAAKDTRSRCPGGKEFLIQYDECMLGYSD |
| AT4G23140.1 | CPNTTTYSSNSTYSTNLRTLLSSLSSRNASYSTGFQNATAGKAPDRVTGLFLCRGDVSPEVCRNCVAFSVNQTLNLCPKVREAVFYYEQCILRYSH |
| AT4G23150.1 | CPNATTYSSNSTYLTNLKTLLSSLSSRNASYSTGFQNATVGQALDRVTGLFLCRGDVSPEVCRNCVTFAVNNTFSRCPNQREAVFYYEECILRYSH |
| AT4G23170.1 | CPNTTTYSRFSTYSTNLRTLLSSFASRNASYSTGFQNVTVGQTPDLVTGLFLCRGDLSPEVCSNCVAFSVDEALTRCPSQREAVFYYEECILRYSD |
| AT4G23180.1 | CQNTANYTSNSTYNNNLKTLLASLSSRNASYSTGFQNATVGQAPDRVTGLFNCRGDVSTEVCRRCVSFAVNDTLTRCPNQKEATLYYDECVLRYSNQ |
| AT4G23190.1 | CTTDKGTFRPNGTYDVNRRLILSSLPSNVTDQDGLYYNGSIGQQPNRVYAIGMCIPGSTSEDCSDCIKKESEFFLKNCPNQTEAYSWPGEPTLCYVRYSN |
| AT4G23200.1 | TYFIPNSTYDTNRRVILSLLPSNVTSHFGFFNGSIGQAPNRVYAVGMCLPGTEEESCIGCLLSASNTLLETCLTEENALIWIANRTICMIRYSD |
| AT4G23210.1 | CIENRKYFTPNGTYDSNRRLILSSLPNNTASRDGFYYGSIGEEQDRVYALGMCIPKSTPSDCSNCIKGAAGWLIQDCVNQTDAYYWALDPTLCLVRYSN |
| AT4G23260.1 | CDNTTGTFIPNSPYDKNRRLILSTLASNVTAQEGYFIGSIGIAPDQVFATGMCAPGSERDVCSLCIRSTSESLLQSCLDQADAFFWSGEETLCLVRYAN |
| AT4G23270.1 | CSVTTTFSSNSTYSTNLKTLLSSLSSLNASSYSTGFQTATAGQAPDRVTGLFLCRVDVSSEVCRSCVTFAVNETLTRCPKDKEGVFYYEQCLLRYSN |
| AT4G23280.1 | CSITTTYSSNSTYSTNLKTLLSSLSSRNASYSTGFQNATAGQAPDMVTGLFLCRGNVSPEVCRSCIALSVNESLSRCPNEREAVFYYEQCMLRYSN |

| | |
|---|---|
| AT4G23300.1 | CIENRKYFTPNGTYDSNRRLILSSLPNNTASQDGFYYGSIGEEQDRVYALGMCIPRSTPSDCFNCIKGAAG WLIQDCVNQTDAYYWALDPTLCLVRYSN |
| AT4G28670.1 | CNNGTVSNEEAYRRSYQINLDAIRGDMRHVKFGTHEHGDPPERMYVLSQCVSDLSSDECSLCWSRATD LLSQCFPATGGWFHLDGCFVRADNY |
| AT4G38830.1 | CSNVTGNFTVNTPYAVNLDRLISSLSSLRRNVNGFYNISVGDSDEKVNSISQCRGDVKLEVCINCIAMAGK RLVTLCPVQKEAIIWYDKCTFRYSN |
| AT5G37660.1 | CSQQKFSPASAYESNLNSLLTSLVNSATYSSYNNFTIMGSSSSDTARGLFQCRGDLSMPDCATCVARAVS QVGPLCPFTCGGALQLAGCYIKYDN |
| AT5G40380.1 | TFVEDMHSLSLKLTTRRFATESLNSTTSIYALIQCHDDLSPSDCQLCYAIARTRIPRCLPSSSARIFLDGCFLR YETY |
| AT5G41290.1 | CMKSSRNTTSNTTYNKNLNTLLSTLSNQSSFANYYNLTTGLASDTVHGMFLCTGDVNRTTCNACVKNAT IEIAKNCTNHREAIIYNVDCMVRYSD |
| AT5G41300.1 | CIDSSRNTTGNTTYNKNLNTMLSTFRNQSSIVNNYNLTTGLASDTVYGMFLCTGDVNITTCNNCVKNATI EIVKNCTNHREAIIYYIDCMVRYSD |
| AT5G43980.1 | CANQKSPDPTGVFSQNLKNLFTSLVSQSSQSSFASVTSGTDNTTAVIGVFQCRGDLQNAQCYDCVSKIPK LVSKLCGGGRDDGNVVAARVHLAGCYIRYES |
| AT5G48540.1 | CNTNSNISASSQVSKNIDSLLATLVSKTPSKGFKTTTSSSYNNKEKVYGLAQCRGDISNTDCSTCIQDAAKK IREVCQNQSDSRILYDFCFLRYSQ |
| EFJ11427 | AQMGRNLLTVLATLQDQGPAAGGYKSTSFGSMSDTVYGFIQCRGDISAQECSSCARSAVQILNSTCVRN STKGGRIHPGNCFLRYED |
| EFJ16458 | CGEEKFTNGSSYSTALDNLLATLITNAPVTGFFQSSVQSAELTLYGLLQCRQDMSRDSCDQCARDIGQAV KSRCSSSLGAKIQLYGCFLRYEN |
| EFJ17937 | CGEEKFTNGSSYSTALDNLLATLITNAPVTGFFQSSVQSAELTLYGLLQCRQDMSRDSCDQCARDIGQAV KSRCSSSLGAKIQLYGCFLRYEN |
| LOC_Os01g23970.1 | CNATAGNHTAVGSAYLSNLRALGGALSRRALATGFASGSYGAAPDEVHGLVLCRGDFTGGNCTDGLAS AFRDAAAQFCPGAADATVYYDQYMIRYTN |
| LOC_Os01g26390.1 | CQDSRGKYTSNSTYQANIQSLSSTLPAKAAAPSTGLFATRVAGNAPDTVYALAFCRGDITNASACAGCVA SGFQDAQQLCPFNKAASLYYDLCLLRFAD |
| LOC_Os01g36790.1 | MDDLNSNVSANGFGTSAVGTTAGLNPNAVFGLGQCYRDLSPVDCKLCFAEVRSLLPKCYPSAGGRLYLD GCFGRYANY |
| LOC_Os01g38850.1 | CSLSGGKYEPNSTYEANLRALASLLLAEARATAFASDSFGAAPDAVYGIALCRGDYAGDACAGGLRKAFR DAIDHGVFCAGFRDVTVYYDEHMFRFS |
| LOC_Os02g43000.1 | CSPSKYEPNTAFQSNLNSLLSSIASTASSGAAYNSFTAGGGAGPDPAAGTAAYGLYQCRGDLSPGDCVAC VRQTVARLGAVCANAYAASLQVDGCYVRYD |
| LOC_Os02g50200.1 | CSQGRYASGTQYASDVDSVLTSVANSAPYSPYANFTSPTSNSVVGVYQCRSDLPASVCTGCVRSAISRLSS LCAWATGGAVQLRACFVRYGN |
| LOC_Os03g16950.1 | AGNSKAVASINSVLTDLVAKGSTGGGFATSSAGKANNVIYGLAQCRGDVSTSDCQACLASAANQILTSC NYQSDSRIWYDYCFMRFEN |
| LOC_Os03g16960.1 | SKAVASINSVLTDLVTKGSTGVGFATSTAGKGNNVIYGLVQCRGDVSTSDCQACLASAANQILTSCNYQS DSRIWYDYCFMRFEN |
| LOC_Os03g36650.1 | CSATDSFAADSSFAGNLGRLVSLLEAKAPAIGFDIATVGVGGDGEDQRVHGLALCRGDVARATCAECIRA AGALARRVCPSKKDAVVWLDACMLRYS |
| LOC_Os04g09780.1 | CSPQNYTAGSAYGTSLRGVLKDVVDAAVSGGGYAVANDAGGAAHGLAICYADAPPEVCRLCLAMAAG NLSLACPRAVGGAMLYNNCLLRYA |
| LOC_Os04g25060.1 | CDTSAGTYKAGSAYESNLRDLAAALRADAAASPSALFATGNRGGAPDAVYGLLLCRGDLSVSDCFDCGT RVLADVGRVCGGRHGRAKDVALVYNQCYARFSN |
| LOC_Os04g25650.1 | CDTSAGTYKAGSAYESNLRDLAAALRAGAAASPSALFATGIRGGAPDAVYGLLLCRGDLSVSDCFDCGTR VLADVGRVCGGRHGRAKDVALVYNQCYARFSN |

| | |
|---|---|
| LOC_Os04g45460.1 | CSPSKYQPGTPFEGNLNSLLASIANAAPNGGYNSFTAGSNGTGDGAAAYGLYQCRGDLGNADCAACVRDAVGQLNEVCAAAYAASLQLEGCYVRYDS |
| LOC_Os04g56430.1 | CGDGTYEQGSAYENNLLNLALTLRDGASSQEILFSTGSNGAAPNTVYGLLLCRGDISRAACYDCGTSVWRDAGSACRRAKDVALVYNECYARLSD |
| LOC_Os05g02200.1 | QASVAQVLSELVPRASAGYYATATAGRGGDGSAIWGLAQCRGDIPAPDCALCASAAARQLAGACRGRADARVWYDYCFARYDD |
| LOC_Os05g03920.1 | ADQEGFDVSFVNTLELIYQNVTRSGFGAAASGEGADTVYGLGQCMGYLSPTDCQLCYAQSRVKLPHCLPATGGRIYLDGCFLRYG |
| LOC_Os05g41370.1 | CPTTSSTNSSHVDDGAFGANLRALLSSLSAAAAASSSGFAENATGAAPDTAYGLAQCRGGIVGGGNGTSCRSCLDDSVRDAAKACPGEKSAVIISDYCLVRYSN |
| LOC_Os06g14280.1 | CSQARYDAGTQYAADVDTALSALTNSAGYTAYANYTSPSAASGTGLVGVYQCRSDLPAAICGGCVRSAATKLASLCNSAAGAGVQLRACFVRYGN |
| LOC_Os07g30410.1 | CGSSGNYTAGSKYQANLQALAATLPSTASSSSPALFAKDAAGGGDAEPDRVFALTLCRGDTASANASSSSCADCASRAFRDAQSVCPYSKEVAVYYDPCLLYFS |
| LOC_Os07g30510.1 | CGSSGNYTAGSKYQANLQALAATLPSTASSSSPALFAKDAAGGGDAEPDRVFALTLCRGDTASANASSSSCADCASRAFRDAQSVCPYSKEVAVYYDPCLLYFS |
| LOC_Os07g34980.1 | CSNANTITHMPEGTYKTNLLQLAKNLITNVNQTQLHSANGTAGAAGPDTVYGAVLCRGDSSAESCATRLQRVLDTAS |
| LOC_Os07g35004.1 | CNTTAARRTYLPNSTFEANLNGLFAVLSRNASASGYAAGAFGAAPDTAYGLLLCRGDFTGNDCSAARLASSFQQAASSCLYSKDVAVYYDQYQLRYSDQ |
| LOC_Os07g35140.1 | CNGTRGNFTEGSAFGLNLELLAAELPANASSSRSLFASAAVGAAAAPEDRVFGLALCRGDMRDAAACAGCVSGAFQRLRALCGRDRDATYYHDLCVVRYS |
| LOC_Os07g35280.1 | SGDVFAPRSTYQSNLALLSAGLAKNASASPALFAAGGVGDPPDTVYGLALCRGDTTNATACGACVAAAFQDGQQLCAYAREATVFYDPCYLRFS |
| LOC_Os07g35290.1 | CGQSGNFSANSAYQSNLRQLSATLPKNASAALFAAGSLGTVPDIVYALALCRGDANASACESCVDNAFQGGQQLCPYNKDVFIVYDLCYLRFTN |
| LOC_Os07g35300.1 | CGQGGNYSANGTYQSNLAGLSATLPKNASASRTLFAKDSLGAVPDIVYALALCRGDVANATACESCVATAFQDAQQLCPYDKDAFIVYDLCYLAFSN |
| LOC_Os07g35310.1 | CGTTGNFTANSTYQANLDAVAAALPRNISSSPDLFATAMVGAVPEQVSALALCRGDANATECSGCLATAFQDVQNMCAYDKDAAIYYDPCILYYSN |
| LOC_Os07g35330.1 | CASGAYAANSTYEANLAVLAAALPGNASTAAAAGYATATVGAVPDQVSALALCRGDANATACRACVAASFRVARRDCPSSKDATTYQDGCIVRFSD |
| LOC_Os07g35340.1 | NTYVGNSTFEANLNHLAAELPGNVSTAHTGGFAVATVGADPDQVFALALCRGDVNATACRACVAAAFVDGKNACPGINGVTVYEDACVVRFS |
| LOC_Os07g35380.1 | CDEGVGNTYVANSTFEANLNVLAAALSPNVSVAPAGFAVATVGADPDKVFAMALCRGDVNASACSACVAAAFVDGKKDCPGNSGVAMYEDACVARFSRY |
| LOC_Os07g35390.1 | CNDTAGEFPARRSSYLASINLIAATLPGNASASPDLFATAEGVGAPPDQVSALALCRGDANASTCLACLTQAFLDLPNACAYHKVAAIFYDSCLLAYSN |
| LOC_Os07g35410.1 | CDMGVATYAANSTFEANLDRLGAELPANVSAARATGGYAVATVGAAPDLVYALALCRGDVNASACGACVAAAFADGKRSCPGIKGATVSGPGDGCVLRYS |
| LOC_Os07g35540.1 | TNVTGNSAFDRNLGLLAAALAANASAAGAPGFAVRTAGAAPDQVYALALCRGDVNASACRACVAAAFVDAKGVCPGGISLYEDACLIRFT |
| LOC_Os07g35580.1 | CGENGNYTANSTYQANLKQLAAALHKNVSSGTGGGRLFASGAVGAVPDAVYALALCRGDINASACADCVGTIFQDAQQLCPYRKEVSIVYDSCYLRFSN |
| LOC_Os07g35640.1 | CNATAGNYTEGSAYQANVRALASALPANASSSRALFAEGAAGTAPDKVYAIALCRGDTNASSCAACLAAAFDTAQQLCAFNRRATLFNDPCILRYSDQ |

| | |
|---|---|
| LOC_Os07g35650.1 | CDPYSASGRYSENSTFQANVNRLSATLPRNTSSSPAMYATGAAGDVPDKVYGYALCRGDVADAHACERCVAAALRDAPRVCPLVKDALVFHDLCQLRYSN |
| LOC_Os07g35660.1 | CDTAGGNYTEGSTYQANVRALASALPVNASSSRALFAKGAAGAAPDVVYAIALCRGDTNASSCAACVATAFQDAQQLCAFNRRATMFDDPCILRYSDQ |
| LOC_Os07g35690.1 | GNYSKNGTYQVNLDLLSTTLPKNTSSSPAMYATGTVGDVPDKVYGLALCRGDANASACERCVAAALRDAPRRCPLVKDVLVFYDLCQLRYSN |
| LOC_Os07g35700.1 | CGDSGNYTEHGTYHANIQYLATSLPSYASSSPSLFASGSSGTVPDAIYALALCRGDTNSSSCATCVAAAIQSAQELCPLVKTVIVYDDTCILRFAN |
| LOC_Os07g35740.1 | GTYTANSTYDTNLQSLIAALQQNASTSPTLFAAGALGAAPDAVYGLILCRGDVSSSDCYDCGTRAGQDVAPACNRTRDAILVYNQCYTRFS |
| LOC_Os07g35750.1 | CGTGGTYAANSTYETNLLDLISALQGNASSSPTLYASGAVGSGGRDAVYGVMLCRGDLSTSDCNDCGTRAGQDVGRVCNRTRDAALVYNQCYVRVSD |
| LOC_Os07g35790.1 | CGTSGGNYTAGSTYESNLLRLASTLRANASASPTLFASGVRGAGPDAVYGLLLCRGDMNPSDCFDCGTRVGDDVAQACNRTKDAILVYNQCYAQFSD |
| LOC_Os07g35810.1 | CGTSGGNYTAGSTYESNLLRLASTLRANASASPTLFASGVRGVGPDAVYGLLLCRGDMNPSDCFDCGTNVWRDAGPTCNRTKDAILVYNQCYAQFSD |
| LOC_Os07g43560.1 | CNGSSNYTANSAFQRNLGVLAAALPGNASTSPDLLANATVGGAPDTVYALAFCPPIDNQNASGCRACVASAFADARSLCPNNRGAHIIYDGCVLTFS |
| LOC_Os07g43570.1 | CSGRRYAANSSFDASLQQVARTLPGNASSSPLLFATLAVAGEAYALALCQGGTSAGSCNYCVAQTMRDGEHACAGDADVAMYDDICTVRFSD |
| LOC_Os07g47230.1 | CPSTADGTYAPNSTYQSNLAALAAELIENSTEYGSAAGSFGAAPDAVYGVALCRGDSKGPLCAGYLREAFDAAMNRTTSSRPLCELRRNVTLFYDRFQLRFA |
| LOC_Os08g04210.1 | INFVVSDLVAKARTGGGFATSKAGRGYDAFYGLAQCRGDVSGGDCDACLAQAAKQMVSYCNYTSDSRLWYEYCFMRYDNY |
| LOC_Os08g04230.1 | RSINSVVSDLAAKARAGGGFATSSAGRGIDAFYGLAQCRGDVSGGDCDACLAQAAKQMVTNCNYTLDSRIWYEYCFMRY |
| LOC_Os08g04240.1 | RSVNFVVSDLVAKARTGGGFATSRAGRGYDAFYGLAQCRGDVSGGDCDACLAQAAKQIVSNCNYTSDSRIWYEYCFMRYYNY |
| LOC_Os08g04250.1 | RAVNFVVSDLVAKARTGGGFATSKAGRGSEVFYGLAQCRGDVSGGDCDACLAQAAKQMVSNCNYTSDSRIWYEYCFMRYY |
| LOC_Os10g04720.1 | CGNGGNYTANGTYQSNLARLAAALPSNASSSPDHFATATAGQAPDAAYALALCRGDVANATACGDCVAASFQDARRTCPSDKSATIYYDDCLLRFA |
| LOC_Os10g04730.1 | GVFCDNLKFVSATLPNKTSSSPHHYATAAAGQAPDVVYVLALCRGDLNDTACGESVAYTFARLINESCVANYTAGAYYGDCTGVYS |
| LOC_Os10g17950.1 | CNNGSAYAANTTYDTNVHSILATLTARTPNTTTGFATATTGRGTDTEAWGLALCRGDTDRVGCASCLAAVPAVAFNECRGDMDVTVFYDRCLARFS |
| LOC_Os10g17960.1 | CNNGSSYAANTTYHSNVRAVLTALSAITPNSTARFATASAGRGGADAVWGLALCRGDTDRAGCASCLAAVPAVAFGECRGDRDVAVFYDRCLARFS |
| LOC_Os11g28104.1 | CSQYNATPAAAFLAALNATFAVLRANLSSAGGGGGFATAAEPRAAAPAFAMAQCRPYVAGAGSCAACFDAAASRLRARCGAANGGRAILDGCVVRYES |
| LOC_Os11g38850.1 | CGATNYTARSAYESNLERLIAGLAKNASTPSLFGKGAAGAAPDTVYGVALCRGDLPNASACGDCVAGASRVARRACPLAEDVVVADDAGCQLRFS |
| LOC_Os11g45540.1 | CGSSKYTANSIYQSNLDSLLSSSFLVVSGDSSSGALFAKGSRGAAPDTVYAVALCRGDANASACSGCVDAAYAAATARLCPLSKDAAVFYDECALRFSD |
| LOC_Os12g41270.1 | CSSWNNFSVGSGYQVNLFKLLGNLAAGGAAAGSGGFYSGSYGALSDMVFGVAMCYVDRHWTKCRRCLDAATSGAAAFCPYSRRVDVMYDECVLRYSD |
| LOC_Os12g41410.1 | CSTTGNYSGDSQYKKNLDQLLSTLATAATDDGWFNTSSVGTGGDDQVFGLIMCYADRNPTQCKECLAGAPAGITQVCPGSRTVNANYDACLLRYSD |

| | |
|---|---|
| LOC_Os12g41490.1 | CSTAGNYTGDSQYKKNLDQLFTTLSAGAIAGDWFNTSSVGTGADQVFGLIMCYADRNSTQCQECLAGAPAGIVQVCPGSRTADANYDACLLRYSD |
| LOC_Os12g41530.1 | CSTTGNYTGDSQYKKNLDQLFTSLSGGAIAGDWFNTSSVGTGADQVFGLIMCYADSNATECQKCLAMAPAVVQHPCRGSRSVNANYDACLLRYSD |
| SiCBMIP | GAQTQASISQVLAALVPRASAAYYATATAGSGSSAIWGLAQCRGDIPASDCALCISAAAKQAASSCRGQADVRLWYDYCFLRYTD |

## DUF26–B sequences

| | |
|---|---|
| AFP1_DUF26-B | DNPKAFEKAVGKVMGKATAQASAAGSAGLGRDKEQYTPFVSIYGLAQCTRDLAPLTCAQCLSTALSRFGDYCGAQQGCQINYSSCRVRYEI |
| AT1G04520.1 | AGTGFEERRDTAFGVMQNGVVSGHGFYATTYESVYVLGQCEGDVGDTDCSGCVKNALEKAQVECGSSISGQIYLHKCFIAYSY |
| AT1G19090.1 | GEFWRFLDEALVNVTLKAVKNGGFGAASVIKTEAVYALAQCWQTLDENTCRECLVNARSSLRACDGHEARAFFTGCYLKYST |
| AT1G61750.1 | FINTVEYRMDRLIQEAYSSSSYFAEETYHVSYLGEVYDLNGLVQCTPDLNQYDCYRCLKSAYNETKDCCYGKRFALVYSSNCMLTYK |
| AT1G63550.1 | NPTRFNQTLTEKFSELIFNVSSSSLVPYFVEDQERVTQSEGSYDLDTMVQCSPDLDIFNCTVCLRVAFFRISTCCGLPSYAKIFTPKCLLRFQT |
| AT1G63570.1 | NSNRFNQTLSNKLDQLIPNVSPSTLIPYFVEDQERVTQLEGSYDLVSMIQCSPDLDPSNCTICLRFAYATVSTCCGVPSSALIFTPKCILRYR |
| AT1G63580.1 | YNQTLPGKLDELILKAPSSFSSPVPYFVEDKEHVTQVEGSYDLEAMAQCSPDLDPSSCTVCLGLVVEKFSECCSQSRWARIHFPKCLLRYD |
| AT1G63590.1 | FSQTLLEKLDALILRASLSSSLPVPYFVDDQQHVTQLEGSYDLHAMVQCSPDLDPRNCTVCLRLAVQRLSGCCSHAQFARIFYTKCLITYE |
| AT1G63600.1 | PNLDRFPQTLSDKMDELIINATSSPSLSSTPYFVEDQERVKQFEGSFDIDSMAQCSPDLDPRNCTTCLKLAVQEMLECCNQSRWAQIFTPKCLLRYEA |
| AT1G70520.1 | CGNTTRKNKTFGDAVRQGLRNAVTEASGTGGYARASAKAGESESESAFVLANCWRTLSPDSCKQCLENASASVVKGCLPWSEGRALHTGCFLRYSDQ |
| AT1G70530.1 | NRTVFRDNAAELVKNMSVEAVRNGGFYAGFVDRHNVTVHGLAQCWETLNRSGCVECLSKASVRIGSCLVNEEGRVLSAGCYMRFST |
| AT1G70690.1 | RTGGNGNVQGVAQCSGDLSTSQCQDCLSDAIGRLKSDCGMAQGGYVYLSKCYARFS |
| AT2G01660.1 | YRVGVSGELQGVAQCTGDLSATECQDCLMEAIGRLRTDCGGAAWGDVYLAKCYARYSA |
| AT2G31620.1 | NKKLFNKNTKALLDKLKEKAIRKEQEPYTRDYMYAAGEESLGTTKLYGMMQCTQDLSVKNCSVCLDSIIAKLPRCCNGKQGGRVLNPSCTFRYELY |
| AT2G33330.1 | NVAGTGFEQRRDTAFGVMQNGVVQGHGFYATTYESVYVLGQCEGDIGDSDCSGCIKNALQRAQVECGSSISGQIYLHKCFVGYSF |
| AT3G21960.1 | DKKSFYKNMKAFLHKLKAKASSKENKPYVKDYMYAAGRESLGTVKLYAMVQCTQDLSLKNCTVCLDWIMTKLPECCNGKQGGRVLSPSCNF |
| AT3G21970.1 | NVTNDPKRFEDKRRDLLHKLMLEATKDSKENGAKGLLYAVGEMRIGRNKMYAMVQCTQDLWQTGCHVCLEWITQMKYGEFFYRKPGGRVCGRSCSFRYELY |
| AT3G21980.1 | NDKELFNKETSALLEELTNKATDKNNMIGNKFVLYAAGDKRIGTKNVYAMVQCTKDLVTTTSAACFEWIFKMFSKCCEGKQGGRVLGTSCNFRYELY |
| AT3G21990.1 | KNMSDRELFNKETSALLEKLANKASDRNNLDGKQLVLYAAGEKRIGTKKVSAMVQCTKDLIFTKCFECLEGILRKFPECCDGKQGGRVLGTSCNFRYELY |
| AT3G22040.1 | GDTKMFSKKTNDFLQQLIVKADKPDMDGVELLYYAAGEMRIGREKLHAMVQCAKDLADCKSCLEWSFKELSKCCDGKRGARFLGTSCNLRYELY |

| | |
|---|---|
| AT3G22060.1 | NVSDPSTFNSQTKALLTELTKKATTRDNQKLFATGEKNIGKNKLYGLVQCTRDLKSITCKACLNGIIGELPNCCDGKEGGRVVGGSCNFRYEI |
| AT3G29040.1 | KNVSSNAEQFKNKRKDLFHKLLLGATKDVSDSNDAYAVGETRIGRNKMYAMMQCALDLTTNGCYVCLEWIIGRYDSFYFDRRQGTRVLSRSCSLRYELY |
| AT3G45860.1 | NQNQVDEFRDLVSSTLNLAAVEAANSSKKFYTRKVITPQPLYLLVQCTPDLTRQDCLRCLQKSIKGMSLYRIGGRFFYPSCNSRYENY |
| AT3G60720.1 | CSSKSVENDYDFFKRRDDVLSDLESTQLGYKVSRSGLVEGYAQCVGDLSPSDCTACLAESVGKLKNLCGSAVAAEVYLAQCYARY |
| AT4G00970.1 | DRNEFIRLQSELLNRLRSMAASGGSKRKYAQGTDPGSPPYTTFFGAVQCTPDLSEKDCNDCLSYGFSNATKGRVGIRWFCPSCNFQIE |
| AT4G04490.1 | QEFAARANRTVEVASTADESSVLKYYGVSSAEFTDTPEVNMLMQCTPDLSSSDCNHCLRENVRYNQEHNWDRVGGTVARPSCYFRWDDY |
| AT4G04500.1 | NMAMFSQEWIAMVNRTLEAASTAENSSVLKYYSATRTEFTQISDVYALMQCVPDLSPGNCKRCLRECVNDFQKQFWGRQGGGVSRPSCYFRWDL |
| AT4G04510.1 | TLFRQEWEAMVDRTLEAVTIDNSTTVLKYYGALKSEFSEFPNVYMMMQCTPDINSGACKRCLQASVTYFRDQNWGRQGGGICRPSCVFRWEF |
| AT4G04540.1 | KNMTLFEQEWNAMANRTVESATEAETSSVLKYYSAEKAEFTEFPNVYMLMQCTPDITSQDCKTCLGECVTLFKEQVWGRQGGEVYRPSCFFRWDL |
| AT4G04570.1 | TLFKQQWEEMVNRTLEAATKAEGSSVLKYYKAEKAGFTEFPDVYMLMQCTPDLSSRDCKQCLGDCVMYFRKDYMGRKGGMASLPSCYFRWDL |
| AT4G05200.1 | NITENQVSRFNESLPALLIDVAVKAALSSRKFATEKANFTVFQTIYSLVQCTPDLTNQDCESCLRQVINYLPRCCDRSVGGRVIAPSCSFRYELY |
| AT4G11460.1 | NLTDFDRLWERLIAHMVTKASSASIKYLSFDNSRFYAADETNLTNSQMVYALMQCTPDVSPSNCNTCLKQSVDDYVGCCHGKQGGYVYRPSCIFRWDL |
| AT4G11470.1 | AYTRTWDAFMNFMFTRVGQTRYLADISPRINQEPLSPDLIYALMQCIPGISSEDCETCLGKCVDDYQSCCNGFIGGVVNKPVCYFRWD |
| AT4G11480.1 | AYTRTWEEFMNSMITRVGRTRYLADISPRIGSARIYALMQCIRGISSMECETCIRDNVRMYQSCCNGFIGGTIRKPVCFFRWD |
| AT4G11490.1 | IWEALTDRLMSDASSDYNASLSSRRYYAANVTNLTNFQNIYALMLCTPDLEKGACHNCLEKAVSEYGNLRMQRGIVAWPSCCFRWDL |
| AT4G11521.1 | FDRIWDELMSRTITTASRTHGSLSFGHKYYAADVASLTTFQTIYTMVQCTPDVSSGDCEFCLKRTVLDYKKCCRGHIGGAFVRPFCFIRWDL |
| AT4G11530.1 | TVFDRIWEELMLRTITAASLSSSNGSSFGQKYFAAEVASLTTFQTMYAMMQCTPDVSSKDCEFCLKTSVGDYESCCRGKQGGAVIRPSCFVRWDL |
| AT4G20550.1 | NNMSDRGLFNKETSALLEKLAYKASDRNNLDGKQLVLYAAGEKRIGTKKVYAMVQCTKDLIFTKCFECLEGILRKFPQCCDGKRGGRVFGTSCNFR |
| AT4G20560.1 | NNMSDRGLFNKETSALLEKLAYKASDRNNLDGKQLVLYAAGEKRIGTKKVYAMVQCTKDLIFTKCFECLEGILRKFPQCCDGKRGGRVFGTSCNFR |
| AT4G20570.1 | NNMSDRGLFNKETSALLEKLAYKASDRNNLDGKQLVLYAAGEKRIGTKKVYAMVQCTKDLIFTKCFECLEGILRKFPQCCDGKRGGRVFGTSCNFR |
| AT4G20580.1 | NNMSDRGLFNKETSALLEKLAYKASDRNNLDGKQLVLYAAGEKRIGTKKVYAMVQCTKDLIFTKCFECLEGILRKFPQCCDGKRGGRVFGTSCNFR |
| AT4G20590.1 | NNMSDRGLFNKETSALLEKLAYKASDRNNLDGKQLVLYAAGEKRIGTKKVYAMVQCTKDLIFTKCFECLEGILRKFPQCCDGKRGGRVFGTSCNFR |
| AT4G20600.1 | NNMSDRGLFNKETSALLEKLAYKASDRNNLDGKQLVLYAAGEKRIGTKKVYAMVQCTKDLIFTKCFECLEGILRKFPQCCDGKRGGRVFGTSCNFR |
| AT4G20610.1 | NNMSDRGLFNKETSALLEKLAYKASDRNNLDGKQLVLYAAGEKRIGTKKVYAMVQCTKDLIFTKCFECLEGILRKFPQCCDGKRGGRVFGTSCNFR |
| AT4G20620.1 | NNMSDRGLFNKETSALLEKLAYKASDRNNLDGKQLVLYAAGEKRIGTKKVYAMVQCTKDLIFTKCFECLEGILRKFPQCCDGKRGGRVFGTSCNFR |
| AT4G20640.1 | NNMSDRGLFNKETSALLEKLAYKASDRNNLDGKQLVLYAAGEKRIGTKKVYAMVQCTKDLIFTKCFECLEGILRKFPQCCDGKRGGRVFGTSCNFR |

| | |
|---|---|
| AT4G20670.1 | NNMSDRGLFNKETSALLEKLAYKASDRNNLDGKQLVLYAAGEKRIGTKKVYAMVQCTKDLIFTKCFECLEGILRKFPQCCDGKRGGRVFGTSCNFR |
| AT4G21230.1 | SVTDKEGFSKGLGDLLDSLGAKIDAANETKEVKFAAGVKGTIYALAQCTPDLSESDCRICLAQIFAGVPTCCDGKTGGWWTNPSCYFRFEV |
| AT4G21400.1 | ISANRDEFDRLQIELLDRLKGIAAAGGPNRKYAQGSGSGVAGYPQFYGSAHCTPDLSEQDCNDCLVFGFEKIPGCCAGQVGLRWFFPSCSYRFET |
| AT4G21410.1 | NRDDFERLQRGLLDRLKGIAAAGGPNRKYAQGNGSASAGYRRFYGTVQCTPDLSEQDCNDCLVFGFENIPSCCDAEIGLRWFSPSCNFRFET |
| AT4G23130.1 | TADQSDRFNDAVLSLMKKSAEEAANSTSKKFAVKKSDFSSSQSLYASVQCIPDLTSEDCVMCLQQSIKELYFNKVGGRFLVPSCNSRYEV |
| AT4G23140.1 | KQIDGFTSFVSSTMSEAAGKAANSSRKLYTVNTELTAYQNLYGLLQCTPDLTRADCLSCLQSSINGMALSRIGARLYWPSCTARYELY |
| AT4G23150.1 | QFTNLVLSNMNQIAIEAADNPRKFSTIKTELTALQTFYGLVQCTPDLSRQNCMNCLTSSINRMPFSRIGARQFWPSCNSRYELY |
| AT4G23170.1 | SQINQFIVLVQSNMNQAAMEAANSSRKFSTIKTELTELQTLYGLVQCTPDLTSQDCLRCLTRSINRMPLSRIGARQFWPSCNSRYELY |
| AT4G23180.1 | DLLSDLVLPTLNQAATVALNSSKKFGTRKNNFTALQSFYGLVQCTPDLTRQDCSRCLQLVINQIPTDRIGARIINPSCTSRYEI |
| AT4G23190.1 | TEFTKIWEGLMGRMISAASTAKSTPSSSDNHYSADSAVLTPLLNIYALMQCTPDLSSGDCENCLRQSAIDYQSCCSQKRGGVVMRPSCFLRWDL |
| AT4G23200.1 | YKTNETEFNTVWSRLTQRMVQEASSSTDATWSGAKYYTADVAALPDSQTLYAMMQCTPDLSPAECNLCLTESVVNYQSCCLGRQGGSIVRLSCAFRAEL |
| AT4G23210.1 | LTEFKTIWEDLTSRTITAASAARSTPSSSDNHYRVDFANLTKFQNIYALMQCTPDISSDECNNCLQRGVLEYQSCCGNNTGGYVMRPICFFRWQ |
| AT4G23260.1 | NTNQTVFDIEWNNLTSSMIAGITSSSSGGNNSSKYYSDDIALVPDFKNISALMQCTPDVSSEDCNTCLRQNVVDYDNCCRGHQGGVMSRPNCFFRWEV |
| AT4G23270.1 | IDLNASQSLYGMVRCTPDLREQDCLDCLKIGINQVTYDKIGGRILLPSCASRYDNY |
| AT4G23280.1 | KRFAVTKFDLNALQSLYGMVQCTPDLTEQDCLDCLQQSINQVTYDKIGGRTFLPSCTSRYDNY |
| AT4G23300.1 | TDFKNIWEDLTSRTITAASAARSTPSSSDNHYRVDFANLTKFQNIYALMQCTPDISSDECNNCLQRGVLEYQSCCGNNTGGYVMRPICFFRWQ |
| AT4G28670.1 | KEKSAEFKGLVKEVTKSIVEAAPYSRGFSVAKMGIRDLTVYGLGVCWRTLNDELCKLCLADGALSVTSCLPSKEGFALNAGCYLRYSNY |
| AT4G38830.1 | NFTGDRDSWEKSLRGLLEGLKNRASVIGRSKKNFVVGETSGPSFQTLFGLVQCTPDISEEDCSYCLSQGIAKIPSCCDMKMGSYVMSPSCMLAY |
| AT5G37660.1 | YFRAGGSGDVQGMGQCVGDLTVSECQDCLGTAIGRLKNDCGTAVFGDMFLAKCYARYST |
| AT5G40380.1 | FGVAGENGVHALAQCWESLGKEDCRVCLEKAVKEVKRCVSRREGRAMNTGCYLRYSD |
| AT5G41290.1 | LESIVQCTPDLDPRNCTTCLKLALQELTECCGNQVWAFIYTPNCMVSFDTY |
| AT5G41300.1 | SYDLDSLVQCSPHLNPENCTICLEYALQEIIDCCSDKFWAMIFTPNCFVNY |
| AT5G43980.1 | QYESVYVLGQCEGSLGNSDCGECVKDGFEKAKSECGESNSGQVYLQKCFVSYSY |
| AT5G48540.1 | PKKFDNELGALFDKIRSEAVLPKNKGLGKGKTKLTPFVTLNGLVQCTRDLSELDCAQCFATAVGSFMTTCHNKKGCRVLYSSCYVRYEF |
| EFJ11427 | LAIFQSNSRTALEFLKAKVPRNARKFVAAVAGIGSAVPAYALGQCIPDLSAADCASCLAAAENAIASTCRGGGGGVCYYSSCTLFFQ |
| EFJ16458 | IASSPGYDDNLKSALATLSRRGSSFTATVGSGKSQVFVLGECRGDLSSQQCDSCVRVAMRNMTDRCIGNTSGSILLNSCYTRF |

| | |
|---|---|
| EFJ17937 | IASSPGYDDNLKSALATLSRRGSSFTATVGSGKSQVFVLGECRGDLSSQQCDSCVRVAMRNMTDRCIGNTSGSILLNSCYTRF |
| LOC_Os01g23970.1 | AAARFMAKATELMNRTADLAAFGSSSSPSRYATGETWFDEQGVSVVYGLVQCTPDLTGEQCRSCLAGIIAQMPKLFGDASSRPVGGRILGVRCNLRYEK |
| LOC_Os01g26390.1 | LLLFTLLNATAESAASSSRRFTTSRLDVSSLPTLYCLMQCTPDLTAGECAACFEDFPRLTLQYLDGARGGRILATRCTMRYEI |
| LOC_Os01g36790.1 | NYTGANPRGFADAVRAALANVTGVAASAAVPGGGDGYAVGSASAGGATAFALAQCWGSLNATACGQCLRAAAAAAARCAPAAAEGRALYTGCYLRYST |
| LOC_Os01g38850.1 | FGDRVMELINTTAEFAAWNSSKRGYATGEAGFGELDVGATRLGLVEQQCRSSPDLVIFALVQCTPDLSPAGCLSCLSGIASQMPRWFTGAADYRLGGRILGVRCNLRYE |
| LOC_Os02g43000.1 | CSSSTSRDGAFLSSRDGVLGELQAAAGYKLSTSGTVQGVAQCLGDVPANDCTACLAEAVGQLKGACGTALAADVYLAQCYVRY |
| LOC_Os02g50200.1 | GSGGVQAMSQCVGDLGAKACSDCVSAAAGQLKAGCGYATAGEVYLGKCYARF |
| LOC_Os03g16950.1 | DNGKAFQKAVGKVMGKATSQASQAGSGGLGRTKDQYTPFINIYGLAQCTQDLSPLACAQCLSTAVSRFGQYCGAQQGCQINYSSCRVRYEI |
| LOC_Os03g16960.1 | DNAKAFQKAVGKVMSKATAQVSQAGSGGLGRVKDQYTPFINIYGFAQCTRDLSPLTCAQCLSTAVSRFDQYCGAQQGCRILYSSCMVRYEI |
| LOC_Os03g36650.1 | REVSRLMKRLTRTAYLSPLLFAAGEAVAVGGAQRLHGMAQCTKDLSGGDCKMCLESAIDQLLARGCAKEGGKVLGGSCSLRYDF |
| LOC_Os04g09780.1 | GDAAQFGAALSRLMDRLALAAASSSSSSRGRRFAFGQTNITGDGGDSLYAFVQCVDDLSPDDCRRCLQSIAASLPMTRGGRAYSLTCYTRFE |
| LOC_Os04g25060.1 | AAYDRAVTELLAATVRYAVEENPARLFATGQRVGDDARDPGFRNIYSMAQCSPDLPPASCRRCLDGVLARWWQVFPLNGEGARVAGARCYLRSE |
| LOC_Os04g25650.1 | AAYDRAVTELLAATVRYAVEENPARLFATGQRVGDDARDPGFRNIYSMAQCSPDLPPASCRRCLDGVLARWWQVFPLNGEGARVAGARCYLRSE |
| LOC_Os04g45460.1 | CSTSTSGDGDFLKNRDAVLAALQGGLANGYKVSSSGNVQGVSQCLGDLAAGDCTTCLAQAVGQLKGTCGTSLAADVYLAQCYVRY |
| LOC_Os04g56430.1 | ADVAAYDRAVTRLLAATAEYAAGDIARKLFATGQRVGADPGFPNLYATAQCAFDITLEACRGCLEGLVARWWDTFPANVDGARIAGPRCLLRSE |
| LOC_Os05g02200.1 | QNATDPEAFEKAQRKVMARVAADAGDAGGGGLARETARFKDGVTIYGLGWCTRDITAADCGLCVAQAVAEMPNYCRFRRGCRVLYSSCMARYETY |
| LOC_Os05g03920.1 | CSNATVSSPASFAATSAALLRNVTAAAPGARDYYYYSASSSSASALPSVSPRVYAAAQCWRSLNATACAACVATARDRVVGRCLPRAAEGYGLNAGCVVRYSTQ |
| LOC_Os05g41370.1 | DNATQPERFKSLLGTLMGNLTDAAARASSPLMFAAGETDLPPFTKIYGMAQCTRDLAAGDCYRCLVGAVNNIPKCCDGKQGGQVITRSCSIRFEV |
| LOC_Os06g14280.1 | GAAGYVQAMSQCVGDLGAKACTDCVSAASSQLKAGCGYASAGEVYLGKCYARF |
| LOC_Os07g30410.1 | YTTVRMDVVTPPLFSLMQCTPDMSGGDCRQCLQDLVGNTTFNGSVSGVRNIGARCGYRYDTY |
| LOC_Os07g30510.1 | YTTVRMDVVTPPLFSLMQCTPDMSGGDCRQCLQDLVGNTTFNGSVSGVRNIGARCGYRYDTY |
| LOC_Os07g34980.1 | RAQFSQLFSELMEKIAAAVVSRRPVNYLTGRGWFDLKSQTVYALAQCTDGMPPENCRSCLDGIIDEGKKMVGGGLTGGAVLGMRCSLWYQT |
| LOC_Os07g35004.1 | VAAFDALVAELVNAVADRASNATRRYAAGKAGFAPEAMTVYAIAQCTPDLSPPQCRGCLAGIIDQMPKWFSGRVGGRILGVRCDFRYEK |
| LOC_Os07g35140.1 | TTRSFFLSLVGTLFGEMAMYGSYNSSARRYASAVMYVNPQLPTVYGLAQCTPDLSPAQCWHCFQGLQEQNRQWYDGRQGGRILGVRCNFRYESY |
| LOC_Os07g35280.1 | APAEVFDAAVVALLNATADHAAASSPRRFATGVEAFRGWGVRDIYALVQCTPDMSPAGCRSCLAGIISWVNDPDYFSGSPTGRVLGVRCNYWYD |

| | |
|---|---|
| LOC_Os07g35290.1 | NASATAEVFDAAAATLLNATSGYAAANSSRRFATGEEAFDAADPTIYGLSQCTPDMSPDDCRSCLGGIIALIPQYFGRKRGARVIGTRCNYRYEV |
| LOC_Os07g35300.1 | NASAPAEVFDAAVATLLNATSSYAAENSSRRFATGEEAFDAAAATPTIYGLSQCTPDMSPDDCRSCLGRIIALIPRYLSRRKGGRAIGMRCNFRYE |
| LOC_Os07g35310.1 | SDPGRFNGMVAALVNATADYAAHNSTRRYASGEAVLDRESEFPKVYSWAQCTPDLTPAQCGDCLAAIIAKLPRLFTNRIGGRVLGVRCSYRYE |
| LOC_Os07g35330.1 | FNAAVVALMNATVDTAVAAGSGSNNTKKYFATAVEDFDPKHYPKIYGMAQCAPVMTAAQCRSCLGGFVSSIPWFLNGKPGGRVLGIWCNLRYS |
| LOC_Os07g35340.1 | VGWFNAAIAKILAALVDHAVATATGNNSTTKKYFATGEEDFDPNIYGFAQCVPDLTQEQCKECLNTFLFQAKQVYFGKSLSWVGMNSVWCRLMYS |
| LOC_Os07g35380.1 | FNAAVTKILAAMVDHAVTSTTGNSTTKKYFVTGEEEFDPNIYGFAQCVPDLTPAQCNDCLKDLLFYAKQAYLGKSLSWVRVNSVWCRLMYS |
| LOC_Os07g35390.1 | ATTEQARFNRLVAALVNATADYAARNSTRRRYASGEADFNAEFPKVYSWAQCTPDLTPASCRSCLAQIIGTYIGFFENRVGGFVRAVWCSFQYST |
| LOC_Os07g35410.1 | WFNAAVAKILAALVEHTWATTTSNATAKKYFSTGEEEFNPKIYGFVQCVPDLSPEQCKECVRTLHDQAKIHYMGNSLPWASTYSVWCSLMYS |
| LOC_Os07g35540.1 | WFNAAVAKILAALVEHAWATTTTTTTGNNSTTTIKYFATGEESFNPKIYGFAQCVPVLTPEQCKECLRSLHDNAKTVYMGNSLRWVGIYSVWCRLMYS |
| LOC_Os07g35580.1 | GRYDRAVTGLLNATARYAAGNTNASSRLFATGVMVGFDAQFPKIYAMAQCSPDLSPAQCGLCLGAMVARWWQTFEPNTQGARSVGARCNMRVEL |
| LOC_Os07g35640.1 | DYASAVYDAFSGMLVNATADYAAKDSVRRFGTGEMGFNVFDSPYHNIFSLAQCTPDMSEADCRSCLGDIIRRMMPKYFVGKPGGRVFGVRCNFRFEAY |
| LOC_Os07g35650.1 | AAFDAAVAMLANATAEYAAAANTSRRYGTAEEEGVDGDGDSGRPRMYALAQCTPDKAADVCRACLTTLTTVQLPKLYSGGRTGGGVFGVWCNLRYE |
| LOC_Os07g35660.1 | AAAFDAASGRLVNATAGYAAADPVRRFGTGEVGFDDATYPRIFSLAQCTPDLSEADCRSCLGRIIRWVPQYFAGKPGGRVFGVRCNFRFESY |
| LOC_Os07g35690.1 | AAAAFDAAVAVLVNATADYAAADSSRRYGTGEEEGVDGDSDRPKIYALAQCTPDKTPEVCRTCLSTVIGQLPKEFSGRTGGGMFGVWCNFRYE |
| LOC_Os07g35700.1 | PAFEAAVVRLINTTADYAATDSVRRFGTGEEAFDETTFPKIYSLAQCTPDMAATACRSCLEDIVGRMVSGNLIGRMGGRVLGVRCNLWFEV |
| LOC_Os07g35740.1 | DVAGYDRAVTELLSATLMYAVVNTTRLFATGQRVGADPGFPNIYSAAQCTPDLSPALCRSCLEDLVARWWKTFPRTTVGARIVGTRCSLRSE |
| LOC_Os07g35750.1 | ADVRAYDAAVVSLLNATVRYAVENSTRMFATGQRVGSDPGFSDIYSMAQCSPALSRPLCRSCLDGLVGQWWDTFPVNVEGARIAGTRCNLRSE |
| LOC_Os07g35790.1 | VAGYDRAVTELLNATVRYAVENSTRLFATGQRVGADPGFRNIYSMAQCSPDLSPAQCRSCLDGLVGQWWTGFLFPRNGEGARVAGPRCYLRSE |
| LOC_Os07g35810.1 | TDVAGYDRAVTELLNATVRYAVENSTKLFATGQRVGNDTGFSNIYSMAQCSPDLSPAQCRSCLDGLVGQWWKTFPLNGKGARVAGPRCYLRSE |
| LOC_Os07g43560.1 | SDVGEFNGAIYEVLNATADYTAAAARRFGTGEISFDPTYPVIYSMAWCTPDMAPGRCRACLADTIAQMHAYFNPNAQGARLVGVRCAARYEI |
| LOC_Os07g43570.1 | AAGRFYRLVGELLDATADYAVANSTARFATGDVGVGGYFDGEPFSKIYALAQCTPDLTPAQCRACLASAMEEMTRQVFAASSPGGKVIGERCGLRFE |
| LOC_Os07g47230.1 | AGRFREHVAALLNATARDAAAQPDRYGTGDSWFQEGGSMVYALVQCTRDMDPGRCGACLQRIISEMPRMLDASQIGGRVLGVRCLLRYE |
| LOC_Os08g04210.1 | KAFQKAAGKAMGKATAQAVAVGSSGLGRAKEQYTPFVSVYALAQCTRDLSPPSCAQCLSAAVSKFDKACGSGPGCQIDYSSCWARYEI |
| LOC_Os08g04230.1 | FVNVYALAQCTRDLAPPLCAQCLSTTVSKFAEACGSGQGCQIDYSSCWVRYEI |
| LOC_Os08g04240.1 | DNPKAFQKAAGKAMGKATAQAVAVGRSGLGRAKEQYTPFVSVYALAQCTRDLAPPACARCLSEIVSKFDKTCNSAQGCQIDYSSCWARYEI |
| LOC_Os08g04250.1 | KAFQKVVGKAMVKATTQAVSVGGNGLGRAKEQYTPFVSVYALAQCTRDLAPPACAQCLSSTVSKFDKACGAAQGCQIDYSSCWARYEI |

| | |
|---|---|
| LOC_Os10g04720.1 | ANVRELLTVTARTAAAAARRFATGFMDGSSESKQTLYSLAQCTPDLAAGDCLACLQRLIAMVNSTTSVRLGGRVLLLRCNLRFE |
| LOC_Os10g04730.1 | QQLLSETVERAAGAAGRFATGVVDTGRTFPLVYSLAQCTPDLSAGDCLACLRRLTGMINSTMAVRMGAQIHVTRCYFRYEAY |
| LOC_Os10g17950.1 | AGHFDALVADLAGALADWAAYNSTLRYAAGVMTSGDGFMSTTEDMVHNIYGVVQCTPDQAAAACRACLEALRVDMPKVFAGKMGGRFNAVWCNLRYET |
| LOC_Os10g17960.1 | AGRFDALVARLAGALADWAAYNSTRRYAAGLMASGDGFTSTTEDMVHNIHGVVQCTPDQAAAACRACLETLRVDMPKVFAGRIGGRFDAVWCNLRYET |
| LOC_Os11g28104.1 | ADGSFAGAARGLVGDLAAAAPRAPGLAAAAARGGVYAAAQCVETVGEGGCAQCLAVPARNIDGCPPDSDGRAVDAGCFMRYSD |
| LOC_Os11g38850.1 | LVQETARTAAYNSSPPPPATTTYYATGRMDVSATFPTLYSMAQCTPDLRPGGCWRCLQSINDMTTRYFAGRRGGRILGLWCNFRYETY |
| LOC_Os11g45540.1 | FTQFFIKTMNYIVAQALSTTKHYAAIRVDMDDADASNTVTLPRRLFCLAQCAPDLVEDICYNCLQNFSDLATANFAGRQGGRILALRCNLRYDT |
| LOC_Os12g41270.1 | YTDSRGESLTVYGMVQCGRGLLPEECSKCLRHQLGELTTGLPNNTAGIIRGYSCYSRYD |
| LOC_Os12g41410.1 | ETRWQLMSQLAETAGQTKLRLDTGSTRLGSTSMMYGLAQCTRDLAVSECSTCLSDYIVQLSKIFPNNSWAAIKGYSCYLRYD |
| LOC_Os12g41490.1 | TMNDTRRRLMSQLAERAGDTKLRLDNGSLPYADSKLGTSALYGLAQCTRDLAASECRRCLSGYVDDLSNTFPNNSGGAIKGYSCYLRYH |
| LOC_Os12g41530.1 | RWQLMSQLAERAGDTKLRLDNGSLPYVDSKLGTSALYGLAQCTRDLAASECRRCLSGYVNDLSNTFPNNSGGAIKGYSCYLRYQ |
| SiCBMIP | NASDPAAFDRAERKLMARVAAEAGDAASGGLVRETARFGSATTIYGLGWCTRDITAADCGLCVAQAVAELPNYCQFRRGCRVLYSSCMARYETY |
